# Supplementary material for: Inflammation in liver fibrosis and atrial fibrillation: A prospective population-based proteomic study
Source: JHEP Rep. 2024 Jul 18;6(10):101171. doi: 10.1016/j.jhepr.2024.101171 (PMC11460462; doi:10.1016/j.jhepr.2024.101171)
Supplement: Multimedia component 1 [file mmc1.pdf]

Supplementary material

Inflammation in liver fibrosis and atrial fibrillation: a prospective population-based proteomic study

Joost Boeckmans, Maurice Michel, Alexander Gieswinkel, Oliver Tüscher, Stavros V. Konstantinides, Jochem König, Thomas Münzel, Karl J. Lackner, Jasmin Ghaemi Kerahrodi, Alexander K. Schuster, Philipp S. Wild, Peter R. Galle, Jörn M. Schattenberg.

Table of contents:

1. Abbreviations…………………………………………… p. 2
2. Definitions of diseases and parameters…………………p. 3
3. Supplementary figures……………………………………p. 5
4. Supplementary tables……………………………………..p. 14

1. Abbreviations

AFib: atrial fibrillation

APRI: AST to platelet index

AUC: area under the curve

AXIN1: Axis inhibition protein 1

BMI: body mass index

CCL20: C-C motif chemokine ligand 20

CI: confidence interval

CXCL10: C-X-C motif chemokine ligand 10

DNER: Delta and Notch-like epidermal growth factor-related receptor

eGFR: estimated glomerular filtration rate

FIB-4: fibrosis-4

Flt3L: Fms related receptor tyrosine kinase 3 ligand

HDL: high-density lipoprotein

L: lower

LDL: low-density lipoprotein

NFS: NAFLD fibrosis score

SD: standard deviation

U: upper

y: year

2. Definitions of diseases and parameters

(self-reporting through questionnaires)

Smoking

- Self-report for “active smoking”

Arterial hypertension

- Self-report or
- Systolic blood pressure > 140 mm Hg or
- Diastolic blood pressure > 90 mmHg

Diabetes mellitus

- Self-report or
- Intake of A10 medication or
- HbA1c ≥ 6.5 %

Obesity

- BMI ≥ 30 kg/m^2

Dyslipidemia

- LDL/HDL > 3.5 or
- Intake of C10 medication

Coronary artery disease

- Self-report

Metabolic syndrome

- If you have 3 or more of the following characteristics:
    - Waist ≥ 94 cm (men) / ≥ 80 cm (women)
    - HDL ≤ 40 mg/dL (men) / ≤ 45 mg/dL (women)
    - Glucose > 100 mg/dL (≥ 8h fasting)
    - Triglycerides > 150 mg/dL (≥ 8h fasting)
    - Arterial hypertension

Hyperuricemia

- Uric acid > 7 mg/dL

Atrial fibrillation

- Self-report

Myocardial infarction

- Self-report

Peripheral artery disease

- Self-report

Congestive heart failure

- Self-report

Chronic kidney disease

- Self-report

eGFR

- Calculated via the CKD-EPI-Formula

Family history of myocardial infarction/stroke

- Self-report

3. Supplementary figures

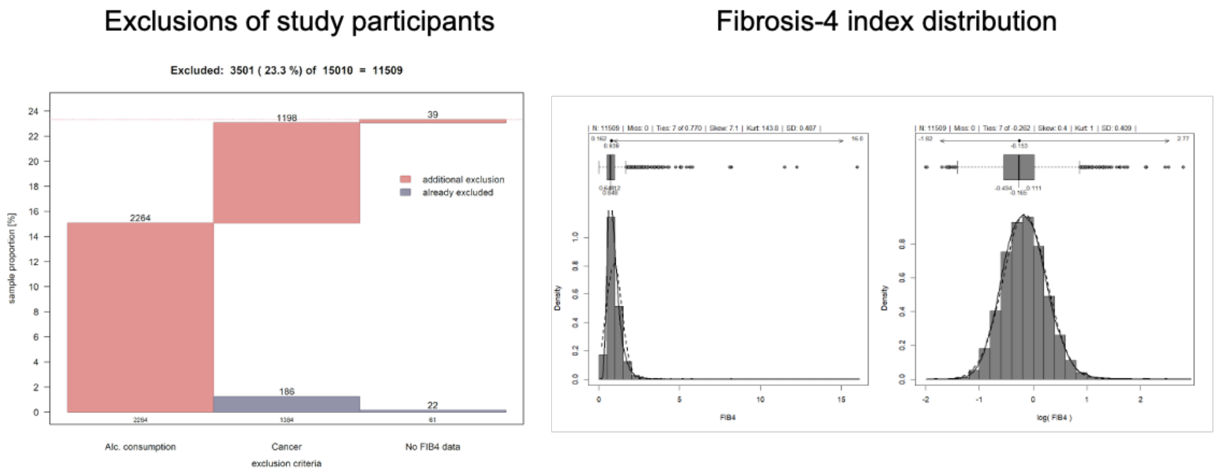

Fig. S1. Exclusions of study participants (left) and distribution of the fibrosis-4 index in de study sample (right). [Abbreviations: FIB-4, fibrosis-4]

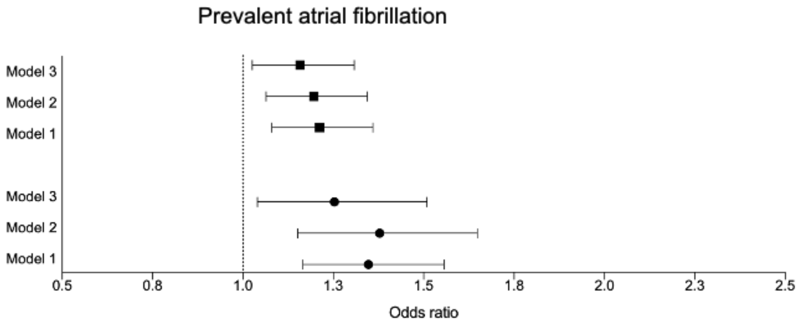

OR	L 95%CI	U 95%CI	p-value
1.158	1.026	1.308	**0.018**
1.196	1.064	1.344	**0.0026**
1.212	1.080	1.359	**0.0010**
1.253	1.040	1.509	**0.017**
1.378	1.152	1.648	**0.00046**
1.347	1.166	1.556	**<0.0001**

■ Log(APRI) per SD
● NFS per SD

Fig. S2. Relationship between the NFS and APRI, and prevalent atrial fibrillation. Relationship between NFS and APRI, and atrial fibrillation (symbols represent odds ratios and bars represent 95% CIs). Levels of significance: exact p-values on the figure, $p < 0.05$ is considered as statistically significant, significant values in bold (multivariate logistic regression, z-test; NFS model 1: N = 11373 (274 events); model 2: N = 11335 (274 events); model 3: N = 11186 (262 events) ; APRI model 1: N = 11395 (275 events); model 2: N = 11335 (274 events); model 3: N = 11186 (262 events)). [model 1: adjusted for age and sex; model 2: additional adjustment for smoking, arterial hypertension, diabetes mellitus, obesity, and dyslipidemia; model 3: additional adjustment for coronary artery disease and congestive heart failure] [Abbreviations: APRI, AST to platelet index; NFS, NAFLD fibrosis score; OR, odds ratio, CI, confidence inteval; L, lower; SD, standard deviation; U, upper]

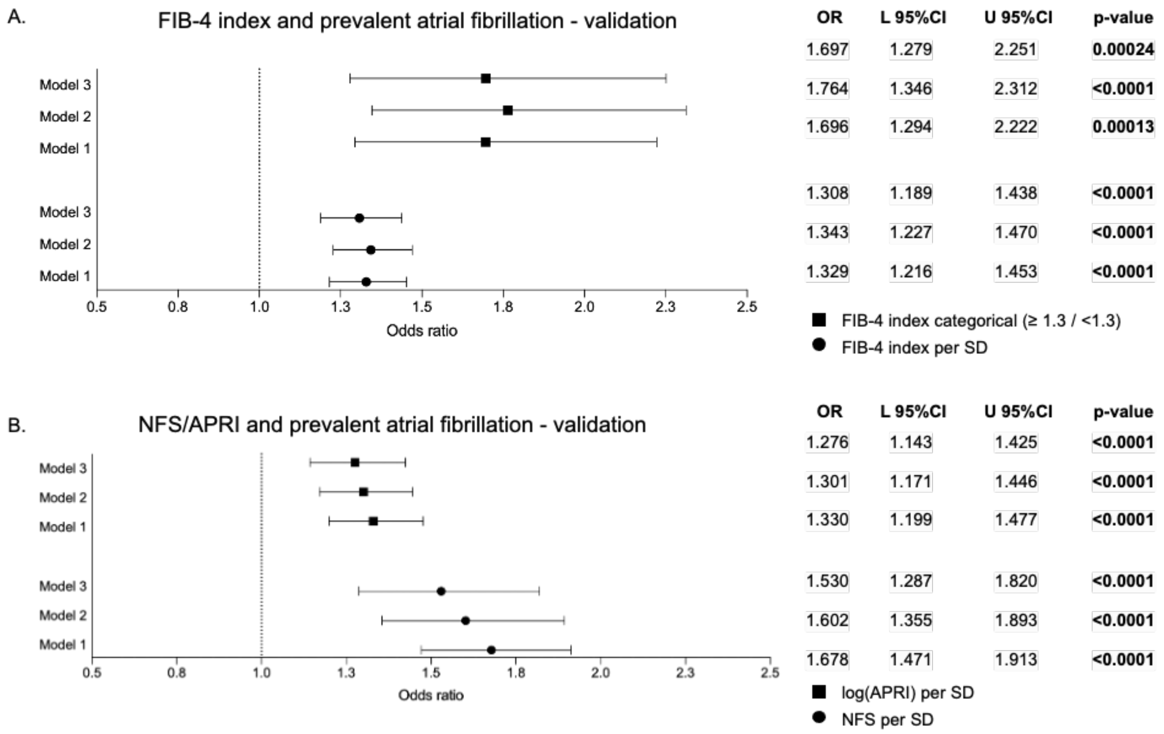

OR	L 95%CI	U 95%CI	p-value
1.697	1.279	2.251	**0.00024**
1.764	1.346	2.312	**<0.0001**
1.696	1.294	2.222	**0.00013**
1.308	1.189	1.438	**<0.0001**
1.343	1.227	1.470	**<0.0001**
1.329	1.216	1.453	**<0.0001**

OR	L 95%CI	U 95%CI	p-value
1.276	1.143	1.425	**<0.0001**
1.301	1.171	1.446	**<0.0001**
1.330	1.199	1.477	**<0.0001**
1.530	1.287	1.820	**<0.0001**
1.602	1.355	1.893	**<0.0001**
1.678	1.471	1.913	**<0.0001**

Fig. S3. Validation of the relationship between non-invasive tests for hepatic fibrosis and atrial fibrillation. (A) Relationship between the FIB-4 index and prevalent atrial fibrillation at 5 years follow-up (symbols represent odds ratios and bars represent 95% CIs). Levels of significance: exact p-values on the figure, $p < 0.05$ is considered as statistically significant, significant values in bold (multivariate logistic regression, z-test; model 1: N = 9290 (325 events); model 2: N = 9265 (325 events); model 3: N = 9192 (312 events)). (B) Relationship between the NFS and APRI and prevalent atrial fibrillation at 5 years follow-up (symbols represent odds ratios and bars represent 95% CIs). Levels of significance: exact p-values on the figure, $p < 0.05$ is considered as statistically significant, significant values in bold (multivariate logistic regression, z-test; NFS model 1: N = 9281 (325 events); model 2: N = 9265 (325 events); model 3: N = 9192 (312 events); APRI model 1: N = 9290 (325 events); model 2: N = 9265 (325 events); model 3: N = 9192 (312 events)). [model 1: adjusted for age and sex; model 2: additional adjustment for smoking, arterial hypertension, diabetes mellitus, obesity, and dyslipidemia; model 3: additional adjustment for coronary artery disease and congestive heart failure] [Abbreviations: APRI, AST to platelet index; CI, confidence inteval; FIB-4, fibrosis-4; L, lower; NFS, NAFLD fibrosis score; SD, standard deviation; U, upper]

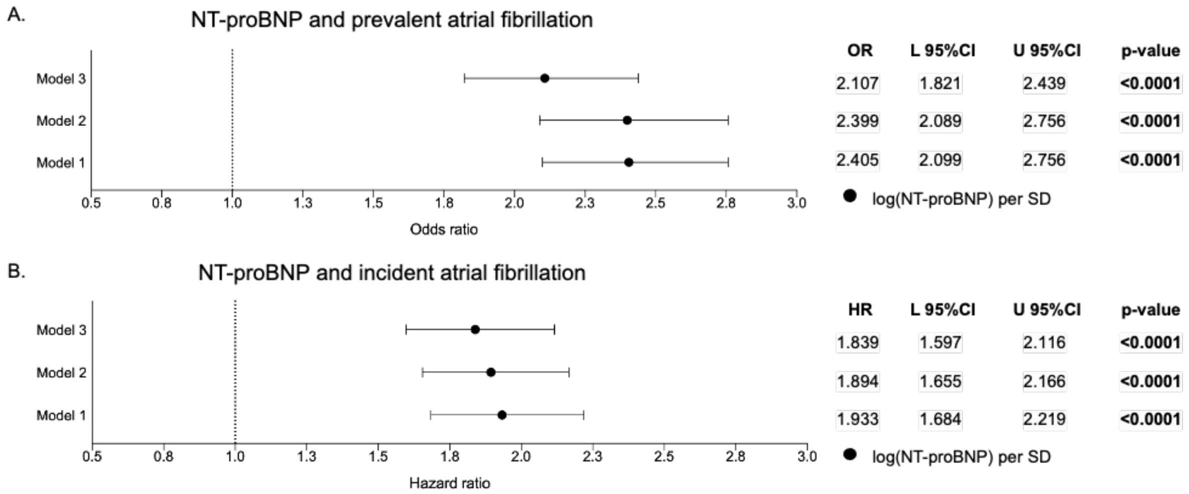

Fig. S4. NT-proBNP in relation to prevalent and incident atrial fibrillation. (A) Relationship between log(NT-proBNP) with prevalent atrial fibrillation (symbols represent odds ratios and bars represent 95% CIs). Levels of significance: exact p-values on the figure, $p < 0.05$ is considered as statistically significant, significant values in bold (multivariate logistic regression, z-test; model 1: N = 11006 (267 events); model 2: N = 10947 (266 events); model 3: N = 10804 (254 events)). (B) Relationship between log(NT-proBNP) and incident atrial fibrillation (symbols represent hazard ratios and bars represent 95% CIs). Levels of significance: exact p-values on the figure, $p < 0.05$ is considered as statistically significant, significant values in bold (Cox competing risk analysis, event = atrial fibrillation, competing event = death, z-test; model 1: N = 10427 (237 events); model 2: N = 10369 (237 events); model 3: N = 10230 (224 events)). [model 1: adjusted for age and sex; model 2: additional adjustment for smoking, arterial hypertension, diabetes mellitus, obesity, and dyslipidemia; model 3: additional adjustment for coronary artery disease and congestive heart failure] [Abbreviations: CI, confidence inteval; HR, hazard ratio; L, lower; NT-proBNP, N-terminal pro–B-type natriuretic peptide; OR, odds ratio; U, upper]

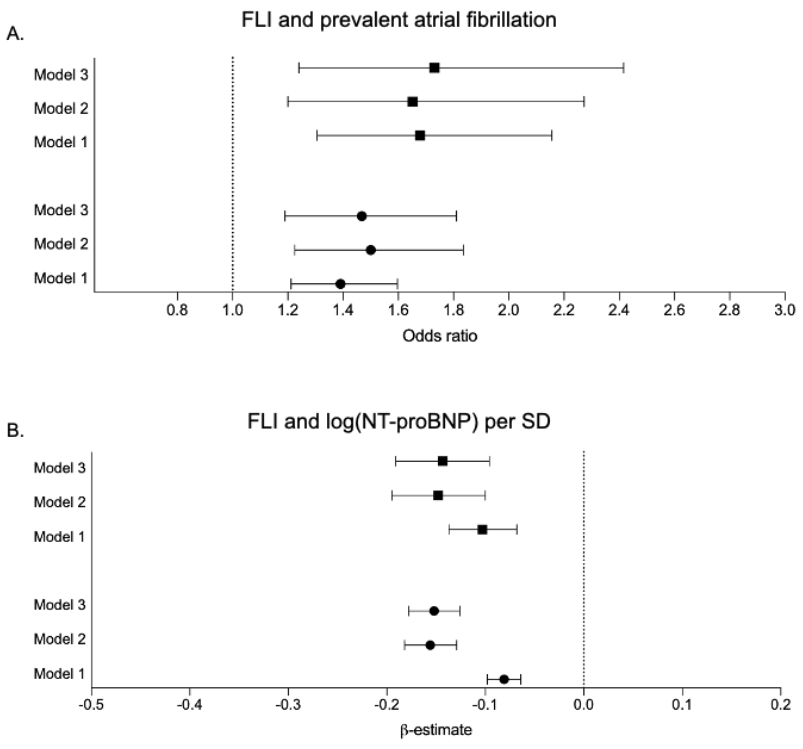

OR	L 95%CI	U 95%CI	p-value
1.731	1.240	2.416	**0.0013**
1.652	1.200	2.273	**0.0021**
1.679	1.306	2.156	**<0.0001**
1.468	1.190	1.811	**0.00034**
1.500	1.225	1.835	**<0.0001**
1.391	1.212	1.597	**<0.0001**

■ FLI categorical (≥ 60 / < 60)
● FLI per SD

β	L 95%CI	U 95%CI	p-value
-0.143	-0.191	-0.096	**<0.0001**
-0.148	-0.195	-0.100	**<0.0001**
-0.103	-0.137	-0.068	**<0.0001**
-0.152	-0.178	-0.126	**<0.0001**
-0.156	-0.182	-0.129	**<0.0001**
-0.081	-0.098	-0.064	**<0.0001**

■ FLI categorical (≥ 60 / < 60)
● FLI per SD

Fig. S5. Relation between the fatty liver index and atrial fibrillation. (A) Relationship between the FLI and prevalent atrial fibrillation (symbols represent odds ratios and bars represent 95% CIs). Levels of significance: exact p-values on the figure, $p < 0.05$ is considered as statistically significant, significant values in bold (multivariate logistic regression, z-test; model 1: N = 11385 (274 events); model 2: N = 11332 (274 events); model 3: N = 11183 (262 events)). (B) Relationship between the FLI and NT-proBNP (symbols represent β-estimates and bars represent 95% CIs) . Levels of significance: exact p-values on the figure, $p < 0.05$ is considered as statistically significant, significant values in bold (multivariate linear regression, t-test; model 1: N = 11105; model 2: N = 11051; model 3: N = 10888). [model 1: adjusted for age and sex; model 2: additional adjustment for smoking, arterial hypertension, diabetes mellitus, obesity, and dyslipidemia; model 3: additional adjustment for coronary artery disease and congestive heart failure] [Abbreviations: FLI, fatty liver index; CI, confidence inteval; L, lower; NT-proBNP, N-terminal pro–B-type natriuretic peptide; U, upper; SD, standard deviation]

	FIB-4 ≥ 1.3	FIB-4 <1.3	AFib	No AFib
P02778 (CXCL10)	44.1	-9.2	58.5	-2.1
Q9H5V8 (CDCP1)	63.6	-13.3	56.2	-2.3
Q9GZV9 (FGF 23)	12.6	-2.6	54.0	-2.2
P78556 (CCL20)	15.8	-3.3	53.1	-2.1
Q07325 (CXCL9)	49.3	-10.3	47.4	-1.9
O15444 (CCL25)	34.3	-7.2	46.9	-2.0
P10147 (CCL3)	35.4	-7.4	44.7	-1.6
P05231 (IL6)	21.3	-4.4	44.3	-1.8
P14210 (HGF)	34.7	-7.2	42.5	-1.7
Q07011 (TNFRSF9)	32.2	-6.7	41.2	-1.5
P80511 (EN RAGE)	11.7	-2.4	40.7	-1.7
P10145 (IL8)	28.9	-6.0	39.6	-1.6
P15692 (VEGFA)	7.7	-1.6	37.4	-1.5
Q13261 (IL 15RA)	29.0	-6.0	36.9	-1.3
P49771 (Flt3L)	38.0	-7.9	36.8	-1.4
Q9NSA1 (FGF 21)	17.2	-3.6	36.5	-1.8
P80098 (MCP 3)	26.6	-5.6	35.4	-1.3
Q14790 (CASP 8)	9.9	-2.1	35.0	-1.3
Q13541 (X4E BP1)	-8.5	1.8	34.4	-1.3
P29460 (IL 12B)	22.5	-4.7	28.9	-1.1
Q9P0M4 (IL 17C)	12.4	-2.6	28.9	-1.1
Q14116 (IL18)	16.5	-3.5	28.4	-1.2
P01135 (TGF alpha)	14.8	-3.1	27.5	-1.0
Q13478 (IL 18R1)	18.1	-3.8	27.2	-1.3
Q99731 (CCL19)	8.1	-1.7	26.9	-1.0
P51671 (CCL11)	24.7	-5.2	26.7	-0.8
P42702 (LIF R)	31.2	-6.5	26.3	-1.2
P13236 (CCL4)	22.0	-4.6	26.0	-0.8
O00300 (OPG)	34.5	-7.2	25.3	-1.2
P28325 (CST5)	30.2	-6.3	24.7	-0.8
Q9NZQ7 (PD L1)	15.2	-3.2	23.5	-0.9
P01375 (TNF)	11.8	-2.5	23.4	-0.6
P01579 (IFN gamma)	9.4	-2.0	20.3	-0.4
Q99616 (MCP 4)	12.8	-2.7	19.7	-0.6
Q08334 (IL 10RB)	17.6	-3.7	19.6	-0.9
P03956 (MMP 1)	-9.3	1.9	19.1	-0.7
P78423 (CX3CL1)	36.1	-7.5	18.9	-0.8
Q13291 (SLAMF1)	20.5	-4.3	18.2	-0.6
P30203 (CD6)	13.2	-2.8	17.8	-0.6
P13500 (MCP 1)	27.1	-5.6	16.9	-0.7
P25942 (CD40)	-2.1	0.4	15.8	-0.7
P01137 (LAP TGF beta 1)	-5.1	1.1	15.7	-0.5
P22301 (IL10)	12.8	-2.7	14.8	-0.3
P09603 (CSF 1)	11.9	-2.5	14.4	-0.6
P80075 (MCP 2)	12.5	-2.6	12.4	-0.5
O95750 (FGF 19)	11.7	-2.5	10.4	-0.5
Q16552 (IL 17A)	3.9	-0.8	10.4	-0.4
P09238 (MMP 10)	4.1	-0.9	10.2	-0.5
O14625 (CXCL11)	4.8	-1.0	9.4	-0.3
P12034 (FGF 5)	10.8	-2.2	8.3	-0.1
P00813 (ADA)	10.1	-2.1	8.3	0.0
Q9BZW8 (CD244)	-4.4	0.9	7.5	-0.3
P55773 (CCL23)	-0.5	0.1	6.7	-0.4
O14788 (TRANCE)	-18.2	3.8	6.7	0.1
P50225 (ST1A1)	-31.7	6.6	6.7	-0.1
P39905 (GDNF)	17.9	-3.7	5.4	-0.1
P01732 (CD8A)	6.6	-1.4	5.1	-0.1
P06127 (CD5)	0.8	-0.2	4.4	-0.2
P13725 (OSM)	-14.2	3.0	3.1	0.0
P80162 (CXCL6)	-10.2	2.1	2.9	0.0
Q9NRJ3 (CCL28)	18.2	-3.8	1.4	0.0
P00749 (uPA)	20.9	-4.4	0.8	0.1
P15018 (LIF)	-5.2	1.1	0.2	0.3
O43557 (TNFSF14)	-16.6	3.5	-1.7	0.1
P13232 (IL7)	-28.9	6.0	-2.1	0.1
P01374 (TNFB)	-3.5	0.7	-2.5	0.3
O95630 (STAMBP)	-36.3	7.6	-4.0	0.1
Q8N6P7 (IL 22 RA1)	-12.2	2.5	-5.0	0.2
P09341 (CXCL1)	-29.5	6.2	-5.7	0.2
P20783 (NT 3)	-14.4	3.0	-6.1	0.4
Q13651 (IL 10RA)	-8.1	1.7	-7.1	0.3
P05112 (IL4)	-7.7	1.6	-8.6	0.3
Q13007 (IL 24)	-7.0	1.5	-9.3	0.3
Q8IXJ6 (SIRT2)	-43.0	9.0	-10.1	0.4
P05113 (IL5)	-12.9	2.7	-10.5	0.6
P50591 (TRAIL)	-15.6	3.3	-11.1	0.7
P01138 (Beta NGF)	2.4	-0.5	-12.1	0.4
P42830 (CXCL5)	-27.1	5.7	-12.2	0.3
Q5T4W7 (ARTN)	-9.8	2.0	-12.4	0.6
Q969D9 (TSLP)	-6.3	1.3	-17.4	0.8
P21583 (SCF)	-0.8	0.2	-18.7	0.7
O95760 (IL33)	-7.9	1.6	-19.8	0.9
P01583 (IL 1 alpha)	-16.5	3.4	-20.6	0.8
P35225 (IL13)	-10.1	2.1	-21.1	1.0
P14784 (IL 2RB)	-8.0	1.7	-22.0	1.0
Q9UHF4 (IL 20RA)	-6.4	1.3	-23.2	0.8
Q9NYY1 (IL 20)	-11.9	2.5	-24.6	1.0
O15169 (AXIN1)	-43.2	9.0	-25.5	0.8
O43508 (TWEAK)	-8.2	1.7	-32.5	1.4
Q99748 (NRTN)	-5.6	1.2	-33.4	1.2
P60568 (IL2)	-18.8	3.9	-37.4	1.6
Q8NFT8 (DNER)	-16.3	3.4	-51.0	2.3

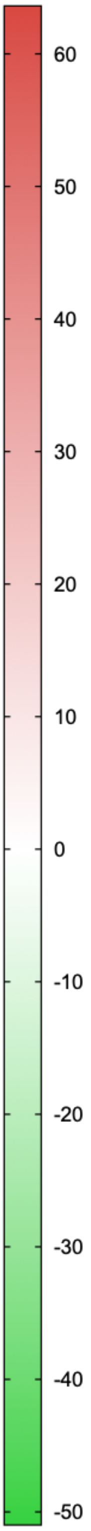

Protein expression
%SD of the overall mean

Fig. S6. Protein expressions in %SD of the overall mean in study participants with a fibrosis-4 index ≥ 1.3 and atrial fibrillation (sorted by atrial fibrillation). (color scale indicates protein expression rate; green = lower expression, and red = higher expression, compared to the overall mean) [Abbreviations: AFib, atrial fibrillation; FIB-4, fibrosis-4; SD, standard deviation]

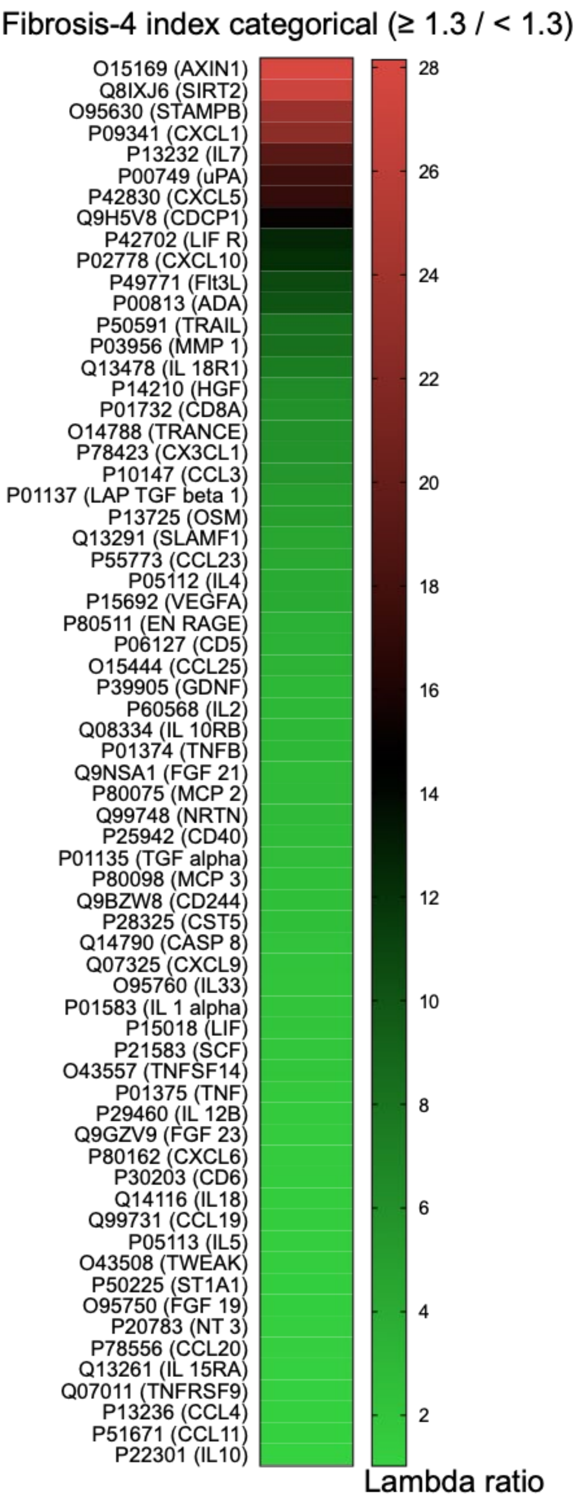

Fig. S7. Selection of the most important proteins related to liver fibrosis using the fibrosis-4 index as a categorical variable. Elastic net regularized regression for circulating proteins in liver fibrosis based on fibrosis-4 index categories (≥ 1.3 / < 1.3) (10 fold cross-validation AUC = 0.8870, (simple AUC = 0.8981), minimal lambda = 0.004 (10 fold-cross validation), N = 5741, events = 991, number of proteins = 92, adjusted for age [SD] and sex). Color scale indicates the lambda ratios within the spectrum of obtained results, green = lower, and red = higher lambda ratio [Abbreviations: AUC, area under the curve; SD, standard deviation]

A.

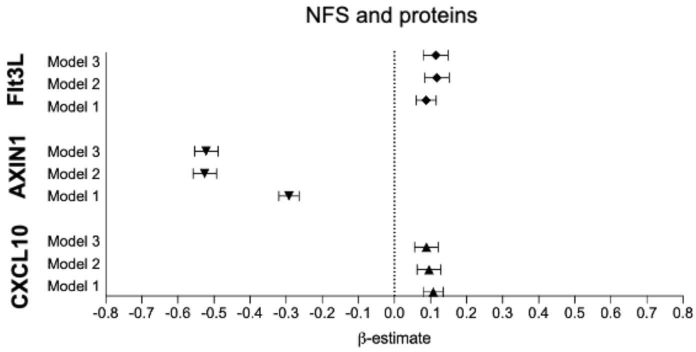

		β	L 95%CI	U 95%CI	p-value
Flt3L	Model 3	0.115	0.080	0.149	**<0.0001**
	Model 2	0.118	0.084	0.152	**<0.0001**
	Model 1	0.088	0.060	0.116	**<0.0001**
AXIN1	Model 3	-0.522	-0.554	-0.489	**<0.0001**
	Model 2	-0.526	-0.558	-0.493	**<0.0001**
	Model 1	-0.292	-0.320	-0.264	**<0.0001**
CXCL10	Model 3	0.089	0.056	0.122	**<0.0001**
	Model 2	0.096	0.063	0.129	**<0.0001**
	Model 1	0.108	0.080	0.135	**<0.0001**

B.

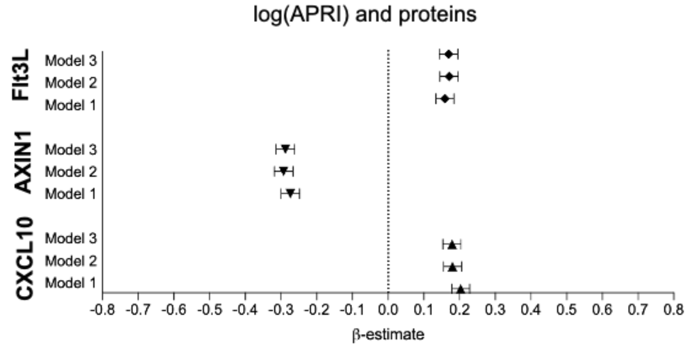

		β	L 95%CI	U 95%CI	p-value
Flt3L	Model 3	0.169	0.143	0.195	**<0.0001**
	Model 2	0.171	0.145	0.196	**<0.0001**
	Model 1	0.159	0.134	0.184	**<0.0001**
AXIN1	Model 3	-0.288	-0.314	-0.262	**<0.0001**
	Model 2	-0.293	-0.319	-0.267	**<0.0001**
	Model 1	-0.274	-0.300	-0.248	**<0.0001**
CXCL10	Model 3	0.179	0.153	0.204	**<0.0001**
	Model 2	0.180	0.155	0.205	**<0.0001**
	Model 1	0.203	0.178	0.228	**<0.0001**

Fig. S8. Relationships between CXCL10, AXIN1, and Flt3L with the NFS and APRI. (A) Relationship between the NFS (per SD) and circulating proteins (per SD) (symbols represent β-estimates and bars represent 95% CIs). Levels of significance: exact p-values on the figure, $p < 0.05$ is considered as statistically significant, significant values in bold (multivariate linear regression, t-test; model 1: N = 5723; model 2: N = 5704; model 3: N = 5601). (B) Relationship between log(APRI) (per SD) and circulating proteins (per SD) (symbols represent β-estimates and bars represent 95% CIs). Levels of significance: exact p-values on the figure, $p < 0.05$ is considered as statistically significant, significant values in bold (multivariate linear regression, t-test; model 1: N = 5741; model 2: N = 5704; model 3: N = 5601). [model 1: adjusted for age and sex; model 2: additional adjustment for smoking, arterial hypertension, diabetes mellitus, obesity, and dyslipidemia; model 3: additional adjustment for coronary artery disease and congestive heart failure] [Abbreviations: APRI, AST to platelet index; CI, confidence inteval; L, lower; NFS, NAFLD fibrosis score; U, upper; SD, standard deviation]

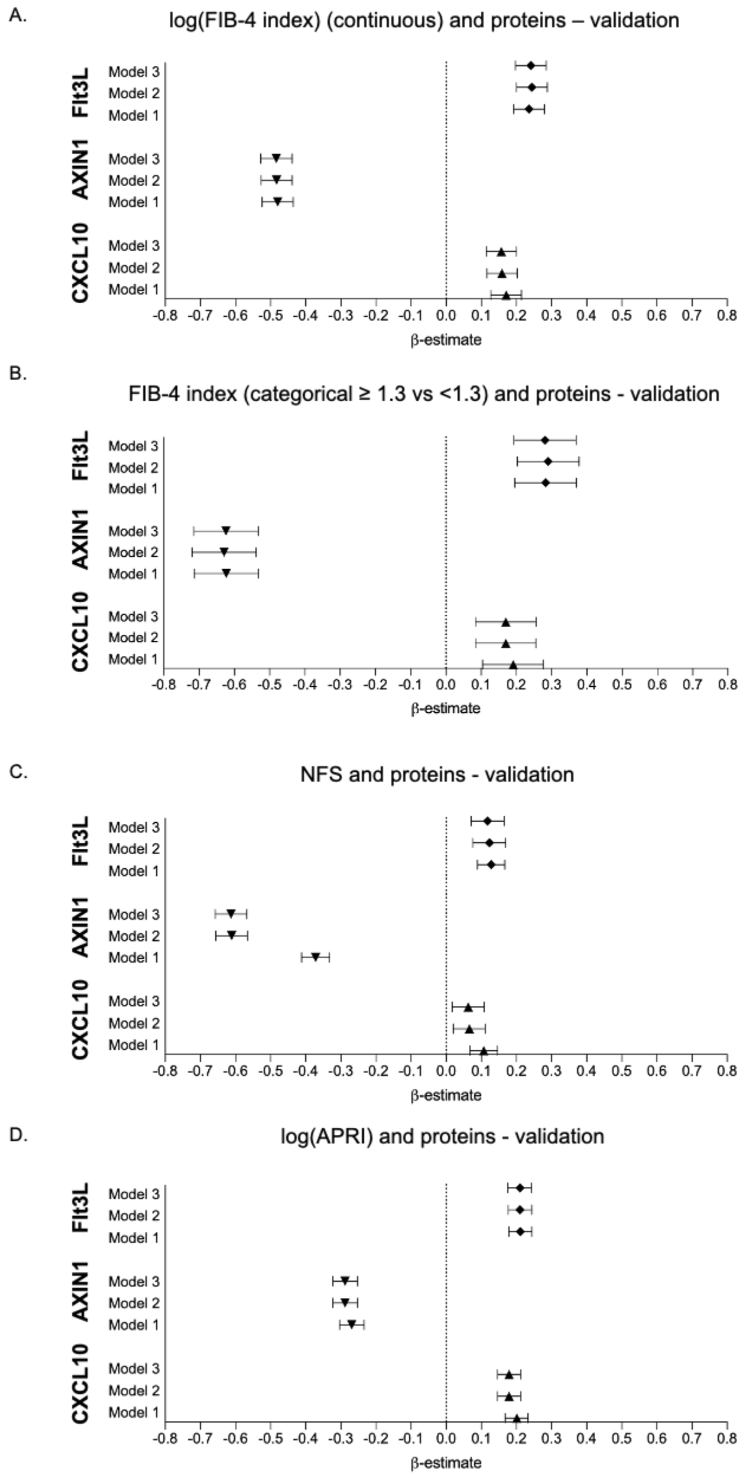

A.

β	L 95%CI	U 95%CI	p-value
0.241	0.197	0.285	**<0.0001**
0.244	0.200	0.288	**<0.0001**
0.236	0.192	0.280	**<0.0001**
-0.484	-0.528	-0.439	**<0.0001**
-0.483	-0.527	-0.439	**<0.000**
-0.480	-0.524	-0.436	**<0.0001**
0.157	0.114	0.200	**<0.0001**
0.159	0.116	0.202	**<0.0001**
0.171	0.127	0.214	**<0.0001**

B.

β	L 95%CI	U 95%CI	p-value
0.281	0.193	0.369	**<0.0001**
0.290	0.203	0.378	**<0.0001**
0.283	0.195	0.370	**<0.0001**
-0.624	-0.715	-0.532	**<0.0001**
-0.629	-0.720	-0.538	**<0.0001**
-0.623	-0.714	-0.532	**<0.0001**
0.170	0.084	0.256	**0.00010**
0.170	0.084	0.255	**<0.0001**
0.191	0.104	0.277	**<0.0001**

C.

β	L 95%CI	U 95%CI	p-value
0.118	0.071	0.165	**<0.0001**
0.123	0.076	0.169	**<0.0001**
0.128	0.089	0.167	**<0.0001**
-0.613	-0.658	-0.568	**<0.0001**
-0.611	-0.656	-0.566	**<0.0001**
-0.372	-0.412	-0.332	**<0.0001**
0.063	0.017	0.108	**0.0071**
0.066	0.020	0.111	**0.0046**
0.107	0.068	0.146	**<0.0001**

D.

β	L 95%CI	U 95%CI	p-value
0.210	0.176	0.243	**<0.0001**
0.210	0.177	0.244	**<0.0001**
0.211	0.178	0.244	**<0.0001**
-0.288	-0.323	-0.253	**<0.0001**
-0.288	-0.323	-0.253	**<0.0001**
-0.269	-0.304	-0.234	**<0.0001**
0.179	0.146	0.212	**<0.0001**
0.179	0.146	0.212	**<0.0001**
0.201	0.168	0.233	**<0.0001**

Fig. S9. Validation of the relationship between circulating proteins and non-invasive tests for liver fibrosis at 5 years follow-up. (A) Relationship between log(FIB-4) (per SD) and circulating proteins (per SD) (symbols represent β-estimates and bars represent 95% CIs). Levels of significance: exact p-values on the figure, $p < 0.05$ is considered as statistically significant, significant values in bold (multivariate linear regression, t-test; model 1: N = 3103; model 2: N = 3093; model 3: N = 3075). (B) Relationship between the FIB-4 index (categorical ≥ 1.3 vs <1.3) and circulating proteins (per SD) (symbols represent β-estimates and bars represent 95% CIs). Levels of significance: exact p-values on

the figure, $p < 0.05$ is considered as statistically significant, significant values in bold (multivariate linear regression, t-test; model 1: N = 3103; model 2: N = 3093; model 3: N = 3075). (C) Relationship between the NFS (per SD) and circulating proteins (per SD) (symbols represent β-estimates and bars represent 95% CIs). Levels of significance: exact p-values on the figure, $p < 0.05$ is considered as statistically significant, significant values in bold (multivariate linear regression, t-test; model 1: N = 3095; model 2: N = 3093; model 3: N = 3075). (D) Relationship between log(APRI) (per SD) and circulating proteins (per SD) (symbols represent β-estimates and bars represent 95% CIs). Levels of significance: exact p-values on the figure, $p < 0.05$ is considered as statistically significant, significant values in bold (multivariate linear regression, t-test; model 1: N = 3103; model 2: N = 3093; model 3: N = 3075). [model 1: adjusted for age and sex; model 2: additional adjustment for smoking, arterial hypertension, diabetes mellitus, obesity, and dyslipidemia; model 3: additional adjustment for coronary artery disease and congestive heart failure] [Abbreviations: APRI, AST to platelet index; CI, confidence inteval; L, lower; NFS, NAFLD fibrosis score; U, upper; SD, standard deviation]

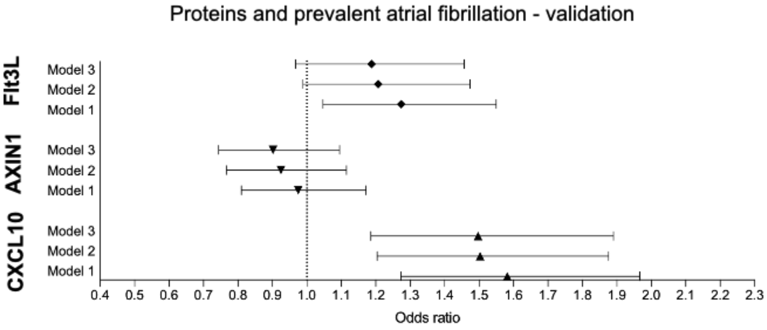

OR	L 95%CI	U 95%CI	p-value
1.188	0.968	1.457	0.099
1.207	0.989	1.474	0.065
1.274	1.047	1.550	**0.016**
0.902	0.744	1.095	0.30
0.925	0.767	1.115	0.41
0.975	0.810	1.172	0.79
1.497	1.186	1.890	**0.00068**
1.503	1.204	1.876	**0.00032**
1.582	1.273	1.967	**<0.0001**

Fig. S10. Validation of the relationship between circulating proteins and prevalent atrial fibrillation at 5 years follow-up. Relationship between circulating proteins (per SD) and atrial fibrillation (symbols represent odds ratios and bars represent 95% CIs). Levels of significance: exact p-values on the figure, $p < 0.05$ is considered as statistically significant, significant values in bold (multivariate logistic regression, z-test; model 1: N = 3100 (116 events); model 2: N = 3090 (116 events); model 3: N = 3073 (113 events)). [model 1: adjusted for age and sex; model 2: additional adjustment for smoking, arterial hypertension, diabetes mellitus, obesity, and dyslipidemia; model 3: additional adjustment for coronary artery disease and congestive heart failure] [Abbreviations: CI, confidence inteval; L, lower; OR, odds ratio; U, upper; SD, standard deviation]

4. Supplementary tables

Table S1: Proteins in the Olink inflammation panel (92 targets)

Protein	UniProt number
Adenosine Deaminase (ADA)	P00813
Artemin (ARTN)	Q5T4W7
Axis inhibition protein 1 (AXIN1)	O15169
Beta-nerve growth factor (Beta-NGF)	P01138
Caspase 8 (CASP-8)	Q14790
C-C motif chemokine 4 (CCL4)	P13236
C-C motif chemokine 19 (CCL19)	Q99731
C-C motif chemokine 20 (CCL20)	P78556
C-C motif chemokine 23 (CCL23)	P55773
C-C motif chemokine 25 (CCL25)	O15444
C-C motif chemokine 28 (CCL28)	Q9NRJ3
CD40L receptor (CD40)	P25942
CUB domain-containing protein 1 (CDCP1)	Q9H5V8
C-X-C motif chemokine 1 (CXCL1)	P09341
C-X-C motif chemokine 5 (CXCL5)	P42830
C-X-C motif chemokine 6 (CXCL6)	P80162
C-X-C motif chemokine 9 (CXCL9)	Q07325
C-X-C motif chemokine 10 (CXCL10)	P02778
C-X-C motif chemokine 11 (CXCL11)	O14625
Cystatin D (CST5)	P28325
Delta and Notch-like epidermal growth factor-related recep (DNER)	Q8NFT8
Eotaxin-1 (CCL11)	P51671
Eukaryotic translation initiation factor 4E-binding protein 1 (4E-BP1)	Q13541
Fibroblast growth factor 5 (FGF-5)	P12034
Fibroblast growth factor 19 (FGF-19)	O95750
Fibroblast growth factor 21 (FGF-21)	Q9NSA1
Fibroblast growth factor 23 (FGF-23)	Q9GZV9
Fms-related tyrosine kinase 3 ligand (Flt3L)	P49771
Fractalkine (CX3CL1)	P78423
Glial cell line-derived neurotrophic factor (GDNF)	P39905
Hepatocyte growth factor (HGF)	P14210
Interferon gamma (IFN-gamma)	P01579
Interleukin-1 alpha (IL-1 alpha)	P01583
Interleukin-2 (IL-2)	P60568
Interleukin-2 receptor subunit beta (IL-2RB)	P14784
Interleukin-4 (IL-4)	P05112
Interleukin-5 (IL-5)	P05113
Interleukin-6 (IL-6)	P05231
Interleukin-7 (IL-7)	P13232
Interleukin-8 (IL-8)	P10145
Interleukin-10 (IL-10)	P22301
Interleukin-10 receptor subunit alpha (IL-10RA)	Q13651
Interleukin-10 receptor subunit beta (IL-10RB)	Q08334
Interleukin-12 subunit beta (IL-12B)	P29460
Interleukin-13 (IL-13)	P35225
Interleukin-15 receptor subunit alpha (IL-15RA)	Q13261
Interleukin-17A (IL-17A)	Q16552
Interleukin-17C (IL-17C)	Q9P0M4
Interleukin-18 (IL-18)	Q14116
Interleukin-18 receptor 1 (IL-18R1)	Q13478
Interleukin-20 (IL-20)	Q9NYY1
Interleukin-20 receptor subunit alpha (IL-20RA)	Q9UHF4
Interleukin-22 receptor subunit alpha-1 (IL-22 RA1)	Q8N6P7
Interleukin-24 (IL-24)	Q13007
Interleukin-33 (IL-33)	O95760
Latency-associated peptide transforming growth factor beta 1 (LAP TGF-beta-1)	P01137
Leukemia inhibitory factor (LIF)	P15018

Leukemia inhibitory factor receptor (LIF-R)	P42702
Macrophage colony-stimulating factor 1 (CSF-1)	P09603
Macrophage inflammatory protein 1-alpha (CCL3)	P10147
Matrix metalloproteinase-1 (MMP-1)	P03956
Matrix metalloproteinase-10 (MMP-10)	P09238
Monocyte chemotactic protein 1 (MCP-1)	P13500
Monocyte chemotactic protein 2 (MCP-2)	P80075
Monocyte chemotactic protein 3 (MCP-3)	P80098
Monocyte chemotactic protein 4 (MCP-4)	Q99616
Natural killer cell receptor 2B4 (CD244)	Q9BZW8
Neurotrophin-3 (NT-3)	P20783
Neurturin (NRTN)	Q99748
Oncostatin-M (OSM)	P13725
Osteoprotegerin (OPG)	O00300
Programmed cell death 1 ligand 1 (PD-L1)	Q9NZQ7
Protein S100-A12 (EN-RAGE)	P80511
Signaling lymphocytic activation molecule (SLAMF1)	Q13291
SIR2-like protein 2 (SIRT2)	Q8IXJ6
STAM-binding protein (STAMPB)	O95630
Stem cell factor (SCF)	P21583
Sulfotransferase 1A1 (ST1A1)	P50225
T-cell surface glycoprotein CD5 (CD5)	P06127
T-cell surface glycoprotein CD6 isoform (CD6)	P30203
T-cell surface glycoprotein CD8 alpha chain (CD8A)	P01732
Thymic stromal lymphopoietin (TSLP)	Q969D9
TNF-beta (TNFB)	P01374
TNF-related activation-induced cytokine (TRANCE)	O14788
TNF-related apoptosis-inducing ligand (TRAIL)	P50591
Transforming growth factor alpha (TGF-alpha)	P01135
Tumor necrosis factor (Ligand) superfamily, member 12 (TWEAK)	O43508
Tumor necrosis factor (TNF)	P01375
Tumor necrosis factor ligand superfamily member 14 (TNFSF14)	O43557
Tumor necrosis factor receptor superfamily member 9 (TNFRSF9)	Q07011
Urokinase-type plasminogen activator (uPA)	P00749
Vascular endothelial growth factor A (VEGF-A)	P15692

Table S2 A. Multivariate logistic regression fibrosis-4 index (continuous per SD) – atrial fibrillation

(Level of significance: $p < 0.05$ is considered as statistically significant (z-test), in bold)

**Model 1**	AUC	N	Odds ratio	L 95%CI	U 95%CI	p-value
Atrial fibrillation	0.7734	11395 (275 events)				
FIB-4 [SD]			1.125	1.038	1.219	**0.0040**
Age [y]			1.094	1.078	1.109	**<0.0001**
Sex (Women)			0.524	0.406	0.675	**<0.0001**
**Model 2**	AUC	N	Odds ratio	L 95%CI	U 95%CI	p-value
Atrial fibrillation	0.7834	11335 (274 events)				
FIB-4 [SD]			1.126	1.039	1.221	**0.0038**
Age [y]			1.086	1.069	1.103	**<0.0001**
Sex (Women)			0.552	0.427	0.715	**<0.0001**
Smoking			0.931	0.636	1.362	0.71
Arterial hypertension			1.079	0.816	1.427	0.59
Diabetes mellitus			1.067	0.761	1.497	0.71
Obesity			1.212	0.926	1.588	0.16
Dyslipidemia			1.602	1.242	2.066	**0.00029**
**Model 3**	AUC	N	Odds ratio	L 95%CI	U 95%CI	p-value
Atrial fibrillation	0.8056	11186 (262 events)				
FIB-4 [SD]			1.100	1.011	1.196	**0.026**
Age [y]			1.077	1.060	1.094	**<0.0001**
Sex (Women)			0.551	0.420	0.724	**<0.0001**
Smoking			0.991	0.674	1.456	0.96
Arterial hypertension			0.998	0.747	1.334	0.99
Diabetes mellitus			1.040	0.727	1.488	0.83
Obesity			1.117	0.840	1.485	0.45
Dyslipidemia			1.414	1.075	1.861	**0.013**
Congestive heart failure			7.871	5.039	12.296	**<0.0001**
Coronary artery disease			1.935	1.330	2.814	**0.00056**

Table S2 B. Multivariate logistic regression fibrosis-4 index (categorical <1.3 / ≥ 1.3) – atrial fibrillation

(Level of significance: $p < 0.05$ is considered as statistically significant (z-test), in bold)

**Model 1**	AUC	N	Odds ratio	L 95%CI	U 95%CI	p-value
Atrial fibrillation	0.7735	11395 (275 events)				
FIB-4 ≥ 1.3			1.463	1.109	1.930	**0.0072**
Age [y]			1.092	1.076	1.108	**<0.0001**
Sex (Women)			0.534	0.413	0.689	**<0.0001**
**Model 2**	AUC	N	Odds ratio	L 95%CI	U 95%CI	p-value
Atrial fibrillation	0.7841	11335 (274 events)				
FIB-4 ≥ 1.3			1.489	1.127	1.966	**0.0050**
Age [y]			1.084	1.067	1.101	**<0.0001**
Sex (Women)			0.564	0.435	0.732	**<0.0001**
Smoking			0.929	0.635	1.361	0.71
Arterial hypertension			1.071	0.810	1.416	0.63
Diabetes mellitus			1.070	0.763	1.500	0.70
Obesity			1.227	0.937	1.606	0.14
Dyslipidemia			1.607	1.246	2.073	**0.00026**
**Model 3**	AUC	N	Odds ratio	L 95%CI	U 95%CI	p-value
Atrial fibrillation	0.8059	11186 (262 events)				
FIB-4 ≥ 1.3			1.363	1.017	1.826	**0.038**
Age [y]			1.076	1.058	1.093	**<0.0001**
Sex (Women)			0.560	0.426	0.737	**<0.0001**
Smoking			0.987	0.671	1.451	0.95
Arterial hypertension			0.990	0.741	1.323	0.95
Diabetes mellitus			1.043	0.729	1.492	0.82
Obesity			1.126	0.847	1.497	0.42
Dyslipidemia			1.411	1.072	1.857	**0.014**
Congestive heart failure			7.818	5.000	12.223	**<0.0001**
Coronary artery disease			1.952	1.343	2.837	**0.00045**

Table S3. Study participants characteristics stratified by the fibrosis-4 index at 5 years follow-up. Data presented as mean +/- standard deviation (Gaussian-distributed data), median with interquartile range (non-Gaussian distributed data), or as relative and absolute frequencies (categorical data); a two-sided t-test was used for comparing two Gaussian-distributed continuous variables, a Wilcoxon rank sum test for non-Gaussian distributed variables, and a chi-square for categorical data; Level of significance: a p-value < 0.05 is considered as statistically significant, in bold.

**Variable**	**Whole sample (9300)**	**Fibrosis-4 index < 1.3 (6041)**	**Fibrosis-4 index ≥ 1.3 (3259)**	**p-value**
		**Demographics**		
Sex (Women)	50.3% (4677)	53.7% (3243)	44.0% (1434)	**<0.0001**
Age [y]	58.2 +/- 10.8	54.3 +/- 9.4	65.5 +/- 9.3	**<0.0001**
BMI [kg/m^2]	26.7 (24.0/30.3)	26.6 (23.9/30.1)	27.0 (24.2/30.5)	**0.00028**
		**Cardiovascular risk factors**		
Dyslipidaemia	33.6% (3122)	30.5% (1843)	39.3% (1279)	**<0.0001**
Arterial hypertension	56.1% (5214)	49.4% (2984)	68.4% (2230)	**<0.0001**
Smoking	15.3% (1420)	18.0% (1087)	10.2% (333)	**<0.0001**
Obesity	26.6% (2474)	25.8% (1559)	28.1% (915)	**0.019**
Family history of myocardial infarction/stroke	23.8% (2215)	24.1% (1458)	23.2% (757)	0.33
Diabetes mellitus	11.0% (1019)	9.1% (548)	14.5% (471)	**<0.0001**
		**Comorbidities**		
Coronary artery disease	4.4% (406)	2.3% (139)	8.3% (267)	**<0.0001**
Myocardial infarction	1.1% (100)	0.6% (38)	1.9% (62)	**<0.0001**
Peripheral artery disease	1.7% (152)	1.1% (64)	2.8% (88)	**<0.0001**
Atrial fibrillation	3.5% (325)	1.7% (104)	6.8% (221)	**<0.0001**
Congestive heart failure	2.0% (189)	1.1% (66)	3.8% (123)	**<0.0001**
Chronic kidney disease	0.8% (78)	0.7% (42)	1.1% (36)	**0.043**
		**Liver parameters**		
Fatty liver index	48.30 +/- 29.96	46.50 +/- 29.94	52.73 +/- 29.57	**<0.0001**
Fatty liver index ≥ 60	38.2% (2395)	35.8% (1598)	43.9% (797)	**<0.0001**
Fibrosis-4 index	1.11 (0.86/1.47)	0.93 (0.75/1.10)	1.63 (1.44/1.98)	**<0.0001**
NAFLD fibrosis score	-1.83 +/- 1.29	-2.39 +/- 1.06	-0.78 +/- 0.97	**<0.0001**
AST to platelet index	0.32 (0.26/0.41)	0.29 (0.24/0.34)	0.42 (0.35/0.52)	**<0.0001**
Alanine aminotransferase [U/L]	30.00 (24.00/39.00)	31.00 (25.00/40.00)	29.00 (22.00/38.00)	**<0.0001**
Aspartate aminotransferase [U/L]	25.0 (22.0/30.0)	24.0 (21.0/28.0)	28.0 (24.0/33.0)	**<0.0001**
Gamma-glutamyl transferase [U/L]	24.00 (17.00/36.00)	24.00 (17.00/35.00)	25.00 (18.00/38.83)	**<0.0001**

Table S4 A. Cox competing risk analysis for log(fibrosis-4 index) (continuous per SD) and incident atrial fibrillation (event = atrial fibrillation, competing event = death)

(Level of significance: $p < 0.05$ is considered as statistically significant (z-test), in bold)

**Model 1**	Sample	HR	L95%CI	U95%CI	p-value
Atrial fibrillation ~	C-index: 0.7616, N:10796 (246 events, 166 competing events)				
log(FIB-4) [SD]		1.136	0.953	1.353	0.15
Age [y]		1.088	1.070	1.107	**<0.0001**
Sex (Women)		0.665	0.514	0.862	**0.0020**
**Model 2**	Sample	HR	L95%CI	U95%CI	p-value
Atrial fibrillation ~	C-index: 0.7739, N:10737 (246 events, 164 competing events)				
log(FIB-4) [SD]		1.157	0.977	1.370	0.091
Age [y]		1.080	1.061	1.100	**<0.0001**
Sex (Women)		0.708	0.545	0.919	**0.0096**
Smoking		1.236	0.860	1.777	0.25
Arterial hypertension		1.157	0.863	1.551	0.33
Diabetes mellitus		1.469	1.056	2.043	**0.022**
Obesity		1.391	1.060	1.824	**0.017**
Dyslipidemia		1.240	0.957	1.606	0.10
**Model 3**	Sample	HR	L95%CI	U95%CI	p-value
Atrial fibrillation ~	C-index: 0.7738, N:10591 (232 events, 159 competing events)				
log(FIB-4) [SD]		1.125	0.943	1.342	0.19
Age [y]		1.081	1.061	1.102	**<0.0001**
Sex (Women)		0.723	0.550	0.950	**0.020**
Smoking		1.260	0.871	1.825	0.22
Arterial hypertension		1.107	0.819	1.495	0.51
Diabetes mellitus		1.279	0.895	1.827	0.18
Obesity		1.303	0.980	1.733	0.069
Dyslipidemia		1.159	0.880	1.527	0.29
Congestive heart failure		2.537	1.369	4.701	**0.0031**
Coronary artery disease		1.306	0.844	2.021	0.23

Table S4 B. Cox competing risk analysis for fibrosis-4 index (categorical <1.3 / ≥ 1.3) and incident atrial fibrillation (event = atrial fibrillation, competing event = death)

(Level of significance: $p < 0.05$ is considered as statistically significant (z-test), in bold)

**Model 1**	Sample	HR	L95%CI	U95%CI	p-value
Atrial fibrillation ~	C-index: 0.7613, N:10796 (246 events, 166 competing events)				
FIB-4 (≥1.3)		1.155	0.863	1.545	0.33
Age [y]		1.093	1.078	1.109	**<0.0001**
Sex (Women)		0.652	0.505	0.841	**0.0010**
**Model 2**	Sample	HR	L95%CI	U95%CI	p-value
Atrial fibrillation ~	C-index: 0.7732, N:10737 (246 events, 164 competing events)				
FIB-4 (≥1.3)		1.179	0.880	1.578	0.27
Age [y]		1.08	1.069	1.103	**<0.0001**
Sex (Women)		0.69	0.534	0.895	**0.0051**
Smoking		1.214	0.845	1.743	0.29
Arterial hypertension		1.154	0.861	1.548	0.34
Diabetes mellitus		1.46	1.052	2.034	**0.024**
Obesity		1.393	1.063	1.826	**0.016**
Dyslipidemia		1.236	0.955	1.599	0.11
**Model 3**	Sample	HR	L95%CI	U95%CI	p-value
Atrial fibrillation ~	C-index: 0.7730, N:10591 (232 events, 159 competing events)				
FIB-4 (≥1.3)		1.098	0.809	1.490	0.55
Age [y]		1.087	1.069	1.105	**<0.0001**
Sex (Women)		0.705	0.539	0.923	**0.011**
Smoking		1.237	0.855	1.788	0.26
Arterial hypertension		1.104	0.817	1.491	0.52
Diabetes mellitus		1.270	0.889	1.815	0.19
Obesity		1.303	0.981	1.732	0.068
Dyslipidemia		1.153	0.877	1.516	0.31
Congestive heart failure		2.565	1.382	4.759	**0.0028**
Coronary artery disease		1.327	0.859	2.052	0.20

Table S5 A. Multivariate linear regression for log(fibrosis-4 index) (continuous per SD) and log(NT-proBNP) (continuous per SD)

(Level of significance: $p < 0.05$ is considered as statistically significant (t-test), in bold)

**Model 1**	R^2	N	Estimate	L 95% CI	U 95% CI	p-value
log(NT-proBNP) [SD]	0.2756	11114				
log(FIB-4) [SD]			0.128	0.106	0.149	**<0.0001**
Age [y]			0.031	0.029	0.033	**<0.0001**
Sex (Women)			0.621	0.588	0.653	**<0.0001**
**Model 2**	R^2	N	Estimate	L 95% CI	U 95% CI	p-value
log(NT-proBNP) [SD]	0.2296	11054				
log(FIB-4) [SD]			0.129	0.107	0.151	**<0.0001**
Age [y]			0.030	0.028	0.032	**<0.0001**
Sex (Women)			0.615	0.582	0.648	**<0.0001**
Smoking			0.049	0.008	0.090	**0.020**
Arterial hypertension			0.088	0.052	0.124	**<0.0001**
Diabetes mellitus			0.111	0.052	0.170	**0.00022**
Obesity			-0.053	-0.092	-0.015	**0.0064**
Dyslipidemia			-0.089	-0.125	-0.054	**<0.0001**
**Model 3**	R^2	N	Estimate	L 95% CI	U 95% CI	p-value
log(NT-proBNP) [SD]	0.3010	10891				
log(FIB-4) [SD]			0.117	0.095	0.138	**<0.0001**
Age [y]			0.029	0.027	0.031	**<0.0001**
Sex (Women)			0.633	0.600	0.665	**<0.0001**
Smoking			0.055	0.015	0.096	**0.0071**
Arterial hypertension			0.076	0.041	0.112	**<0.0001**
Diabetes mellitus			0.055	-0.005	0.114	0.071
Obesity			-0.074	-0.112	-0.036	**0.00013**
Dyslipidemia			-0.137	-0.173	-0.101	**<0.0001**
Congestive heart failure			0.727	0.583	0.871	**<0.0001**
Coronary artery disease			0.590	0.506	0.674	**<0.0001**

Table S5 B. Multivariate linear regression for fibrosis-4 index (categorical <1.3 / ≥ 1.3) and log(NT-proBNP) (continuous per SD)

(Level of significance: $p < 0.05$ is considered as statistically significant (t-test), in bold)

**Model 1**	R^2	N	Estimate	L 95% CI	U 95% CI	p-value
log(NT-proBNP) [SD]						
FIB-4 (≥1.3)	0.2790	11114	0.340	0.291	0.389	**<0.0001**
Age [y]			0.034	0.032	0.036	**<0.0001**
Sex (Women)			0.610	0.578	0.642	**<0.0001**
**Model 2**	R^2	N	Estimate	L 95% CI	U 95% CI	p-value
log(NT-proBNP) [SD]	0.2827	11054				
FIB-4 (≥1.3)			0.337	0.288	0.386	**<0.0001**
Age [y]			0.033	0.032	0.035	**<0.0001**
Sex (Women)			0.602	0.569	0.634	**<0.0001**
Smoking			0.039	-0.002	0.080	0.060
Arterial hypertension			0.084	0.048	0.120	**<0.0001**
Diabetes mellitus			0.102	0.043	0.161	**0.00067**
Obesity			-0.056	-0.094	-0.018	**0.0042**
Dyslipidemia			-0.093	-0.129	-0.058	**<0.0001**
**Model 3**	R^2	N	Estimate	L 95% CI	U 95% CI	p-value
log(NT-proBNP) [SD]	0.3035	10891				
FIB-4 (≥1.3)			0.305	0.256	0.354	**<0.0001**
Age [y]			0.032	0.030	0.033	**<0.0001**
Sex (Women)			0.621	0.588	0.653	**<0.0001**
Smoking			0.046	0.006	0.087	**0.023**
Arterial hypertension			0.072	0.037	0.108	**<0.0001**
Diabetes mellitus			0.049	-0.010	0.108	0.11
Obesity			-0.077	-0.115	-0.039	**<0.0001**
Dyslipidemia			-0.141	-0.176	-0.105	**<0.0001**
Congestive heart failure			0.721	0.578	0.865	**<0.0001**
Coronary artery disease			0.583	0.500	0.667	**<0.0001**

Table S6 A. Multivariate linear regression for log(fibrosis-4 index) (continuous per SD) and proteins (per SD)

(Level of significance: $p < 0.05$ is considered as statistically significant (t-test), in bold)

**Model 1**	R^2	N	Estimate	L 95% CI	U 95% CI	p-value
P78556 (CCL20) [SD]	0.0222	5741				
log(FIB-4) [SD]			0.041	0.006	0.075	**0.022**
Age [y]			0.011	0.007	0.014	**<0.0001**
Sex (Women)			-0.068	-0.120	-0.016	**0.011**
**Model 2**	R^2	N	Estimate	L 95% CI	U 95% CI	p-value
P78556 (CCL20) [SD]	0.0700	5704				
log(FIB-4) [SD]			0.068	0.034	0.103	**<0.0001**
Age [y]			0.005	0.001	0.008	**0.0054**
Sex (Women)			0.004	-0.048	0.057	0.87
Smoking			0.325	0.260	0.391	**<0.0001**
Arterial hypertension			0.114	0.058	0.171	**<0.0001**
Diabetes mellitus			0.274	0.202	0.346	**<0.0001**
Obesity			0.183	0.125	0.241	**<0.0001**
Dyslipidemia			0.115	0.060	0.169	**<0.0001**
**Model 3**	R^2	N	Estimate	L 95% CI	U 95% CI	p-value
P78556 (CCL20) [SD]	0.0709	5601				
log(FIB-4) [SD]			0.071	0.036	0.105	**<0.0001**
Age [y]			0.004	0.001	0.008	**0.012**
Sex (Women)			-0.002	-0.055	0.051	0.95
Smoking			0.325	0.259	0.390	**<0.0001**
Arterial hypertension			0.114	0.058	0.171	**<0.0001**
Diabetes mellitus			0.276	0.203	0.350	**<0.0001**
Obesity			0.175	0.117	0.234	**<0.0001**
Dyslipidemia			0.110	0.054	0.166	**0.00011**
Congestive heart failure			0.319	0.129	0.508	**0.00098**
Coronary artery disease			-0.049	-0.157	0.060	0.38
**Model 1**	R^2	N	Estimate	L 95% CI	U 95% CI	p-value
Q8NFT8 (DNER) [SD]	0.0373	5741				
log(FIB-4) [SD]			0.036	0.002	0.071	**0.041**
Age [y]			-0.018	-0.021	-0.015	**<0.0001**
Sex (Women)			-0.172	-0.223	-0.120	**<0.0001**
**Model 2**	R^2	N	Estimate	L 95% CI	U 95% CI	p-value
Q8NFT8 (DNER) [SD]	0.0750	5704				
log(FIB-4) [SD]			0.029	-0.005	0.064	0.091
Age [y]			-0.016	-0.019	-0.013	**<0.0001**
Sex (Women)			-0.190	-0.242	-0.137	**<0.0001**
Smoking			-0.024	-0.089	0.041	0.48
Arterial hypertension			0.019	-0.037	0.076	0.50
Diabetes mellitus			0.110	0.038	0.182	**0.0027**
Obesity			-0.429	-0.486	-0.371	**<0.0001**
Dyslipidemia			-0.056	-0.110	-0.001	**0.045**
**Model 3**	R^2	N	Estimate	L 95% CI	U 95% CI	p-value
Q8NFT8 (DNER) [SD]	0.0940	5601				
log(FIB-4) [SD]			0.040	0.006	0.074	**0.021**
Age [y]			-0.014	-0.017	-0.011	**<0.0001**

Sex (Women)			-0.215	-0.267	-0.163	**<0.0001**
Smoking			-0.029	-0.094	0.036	0.38
Arterial hypertension			0.035	-0.021	0.091	0.22
Diabetes mellitus			0.125	0.052	0.197	**0.00074**
Obesity			-0.409	-0.466	-0.351	**<0.0001**
Dyslipidemia			-0.003	-0.059	0.052	0.91
Congestive heart failure			-0.403	-0.591	-0.216	**<0.0001**
Coronary artery disease			-0.537	-0.645	-0.430	**<0.0001**
**Model 1**	R^2	N	Estimate	L 95% CI	U 95% CI	p-value
P02778 (CXCL10) [SD]	0.1217	5741				
log(FIB-4) [SD]			0.180	0.147	0.213	**<0.0001**
Age [y]			0.019	0.016	0.022	**<0.0001**
Sex (Women)			0.146	0.096	0.195	**<0.0001**
**Model 2**	R^2	N	Estimate	L 95% CI	U 95% CI	p-value
P02778 (CXCL10) [SD]	0.1488	5704				
log(FIB-4) [SD]			0.165	0.132	0.197	**<0.0001**
Age [y]			0.016	0.013	0.019	**<0.0001**
Sex (Women)			0.143	0.092	0.193	**<0.0001**
Smoking			-0.340	-0.402	-0.277	**<0.0001**
Arterial hypertension			0.035	-0.019	0.089	0.20
Diabetes mellitus			-0.059	-0.128	0.010	0.093
Obesity			0.196	0.140	0.251	**<0.0001**
Dyslipidemia			0.059	0.007	0.112	**0.026**
**Model 3**	R^2	N	Estimate	L 95% CI	U 95% CI	p-value
P02778 (CXCL10) [SD]	0.1468	5601				
log(FIB-4) [SD]			0.160	0.127	0.194	**<0.0001**
Age [y]			0.016	0.013	0.019	**<0.0001**
Sex (Women)			0.142	0.091	0.193	**<0.0001**
Smoking			-0.331	-0.394	-0.268	**<0.0001**
Arterial hypertension			0.034	-0.020	0.089	0.22
Diabetes mellitus			-0.062	-0.132	0.009	0.085
Obesity			0.188	0.132	0.244	**<0.0001**
Dyslipidemia			0.055	0.001	0.108	**0.047**
Congestive heart failure			0.158	-0.024	0.340	0.089
Coronary artery disease			0.052	-0.052	0.157	0.33
**Model 1**	R^2	N	Estimate	L 95% CI	U 95% CI	p-value
O15169 (AXIN1) [SD]	0.1158	5741				
log(FIB-4) [SD]			-0.457	-0.490	-0.424	**<0.0001**
Age [y]			0.022	0.019	0.025	**<0.0001**
Sex (Women)			-0.151	-0.201	-0.101	**<0.0001**
**Model 2**	R^2	N	Estimate	L 95% CI	U 95% CI	p-value
O15169 (AXIN1) [SD]	0.1305	5704				
log(FIB-4) [SD]			-0.457	-0.490	-0.424	**<0.0001**
Age [y]			0.016	0.013	0.020	**<0.0001**
Sex (Women)			-0.119	-0.170	-0.068	**<0.0001**
Smoking			-0.124	-0.187	-0.060	**0.00013**
Arterial hypertension			0.095	0.040	0.150	**0.00065**
Diabetes mellitus			0.184	0.115	0.254	**<0.0001**
Obesity			0.062	0.006	0.118	**0.030**
Dyslipidemia			0.088	0.035	0.141	**0.0012**

**Model 3**	R^2	N	Estimate	L 95% CI	U 95% CI	p-value
O15169 (AXIN1) [SD]	0.1337	5601				
log(FIB-4) [SD]			-0.451	-0.485	-0.418	**<0.0001**
Age [y]			0.017	0.014	0.020	**<0.0001**
Sex (Women)			-0.128	-0.179	-0.077	**<0.0001**
Smoking			-0.123	-0.187	-0.060	**0.00014**
Arterial hypertension			0.096	0.041	0.150	**0.00066**
Diabetes mellitus			0.191	0.120	0.262	**<0.0001**
Obesity			0.073	0.017	0.130	**0.011**
Dyslipidemia			0.112	0.057	0.166	**<0.0001**
Congestive heart failure			-0.015	-0.199	0.168	0.87
Coronary artery disease			-0.262	-0.368	-0.157	**<0.0001**
**Model 1**	R^2	N	Estimate	L 95% CI	U 95% CI	p-value
P49771 (Flt3L) [SD]	0.1105	5741				
log(FIB-4) [SD]			0.176	0.143	0.210	**<0.0001**
Age [y]			0.017	0.014	0.020	**<0.0001**
Sex (Women)			0.255	0.205	0.305	**<0.0001**
**Model 2**	R^2	N	Estimate	L 95% CI	U 95% CI	p-value
P49771 (Flt3L) [SD]	0.1193	5704				
log(FIB-4) [SD]			0.192	0.159	0.225	**<0.0001**
Age [y]			0.016	0.013	0.019	**<0.0001**
Sex (Women)			0.283	0.232	0.334	**<0.0001**
Smoking			0.216	0.153	0.280	**<0.0001**
Arterial hypertension			0.020	-0.035	0.075	0.48
Diabetes mellitus			0.041	-0.029	0.112	0.25
Obesity			0.014	-0.042	0.070	0.62
Dyslipidemia			0.048	-0.005	0.102	0.075
**Model 3**	R^2	N	Estimate	L 95% CI	U 95% CI	p-value
P49771 (Flt3L) [SD]	0.1190	5601				
log(FIB-4) [SD]			0.189	0.155	0.222	**<0.0001**
Age [y]			0.016	0.012	0.019	**<0.0001**
Sex (Women)			0.287	0.235	0.339	**<0.0001**
Smoking			0.223	0.159	0.287	**<0.0001**
Arterial hypertension			0.025	-0.030	0.081	0.37
Diabetes mellitus			0.029	-0.042	0.101	0.42
Obesity			0.003	-0.054	0.060	0.92
Dyslipidemia			0.041	-0.014	0.095	0.15
Congestive heart failure			0.171	-0.014	0.356	0.070
Coronary artery disease			0.030	-0.076	0.137	0.58

Table S6 B. Multivariate linear regression for fibrosis-4 index (categorical <1.3 / ≥ 1.3) and proteins (per SD)

(Level of significance: p < 0.05 is considered as statistically significant (t-test), in bold)

**Model 1**	R^2	N	Estimate	L 95% CI	U 95% CI	p-value
P78556 (CCL20) [SD]	0.0215	5741				
FIB-4 (≥1.3)			0.035	-0.040	0.109	0.36
Age [y]			0.012	0.010	0.015	**<0.0001**
Sex (Women)			-0.076	-0.128	-0.025	**0.0038**
**Model 2**	R^2	N	Estimate	L 95% CI	U 95% CI	p-value
P78556 (CCL20) [SD]	0.0679	5704				
FIB-4 (≥1.3)			0.055	-0.018	0.128	0.14
Age [y]			0.008	0.005	0.011	**<0.0001**
Sex (Women)			-0.012	-0.064	0.040	0.66
Smoking			0.314	0.249	0.379	**<0.0001**
Arterial hypertension			0.113	0.057	0.170	**<0.0001**
Diabetes mellitus			0.271	0.199	0.343	**<0.0001**
Obesity			0.182	0.124	0.240	**<0.0001**
Dyslipidemia			0.111	0.057	0.166	**<0.0001**
**Model 3**	R^2	N	Estimate	L 95% CI	U 95% CI	p-value
P78556 (CCL20) [SD]	0.0687	5601				
FIB-4 (≥1.3)			0.065	-0.009	0.139	0.087
Age [y]			0.007	0.005	0.010	**<0.0001**
Sex (Women)			-0.017	-0.070	0.035	0.51
Smoking			0.313	0.248	0.378	**<0.0001**
Arterial hypertension			0.113	0.056	0.170	**<0.0001**
Diabetes mellitus			0.273	0.200	0.347	**<0.0001**
Obesity			0.174	0.116	0.233	**<0.0001**
Dyslipidemia			0.106	0.050	0.162	**0.00020**
Congestive heart failure			0.327	0.137	0.517	**0.00074**
Coronary artery disease			-0.043	-0.151	0.066	0.44
**Model 1**	R^2	N	Estimate	L 95% CI	U 95% CI	p-value
Q8NFT8 (DNER) [SD]	0.0370	5741				
FIB-4 (≥1.3)			-0.055	-0.129	0.019	0.14
Age [y]			-0.015	-0.017	-0.012	**<0.0001**
Sex (Women)			-0.187	-0.238	-0.136	**<0.0001**
**Model 2**	R^2	N	Estimate	L 95% CI	U 95% CI	p-value
Q8NFT8 (DNER) [SD]	0.0750	5704				
FIB-4 (≥1.3)			-0.066	-0.139	0.007	0.075
Age [y]			-0.013	-0.016	-0.011	**<0.0001**
Sex (Women)			-0.205	-0.257	-0.153	**<0.0001**
Smoking			-0.032	-0.097	0.033	0.33
Arterial hypertension			0.019	-0.037	0.075	0.51
Diabetes mellitus			0.110	0.038	0.182	**0.0028**
Obesity			-0.430	-0.488	-0.372	**<0.0001**
Dyslipidemia			-0.058	-0.112	-0.003	**0.038**
**Model 3**	R^2	N	Estimate	L 95% CI	U 95% CI	p-value
Q8NFT8 (DNER) [SD]	0.0934	5601				
FIB-4 (≥1.3)			-0.049	-0.122	0.025	0.20
Age [y]			-0.011	-0.014	-0.008	**<0.0001**

Sex (Women)			-0.231	-0.283	-0.180	**<0.0001**
Smoking			-0.039	-0.104	0.026	0.24
Arterial hypertension			0.034	-0.022	0.090	0.23
Diabetes mellitus			0.123	0.051	0.196	**0.00087**
Obesity			-0.410	-0.468	-0.353	**<0.0001**
Dyslipidemia			-0.007	-0.062	0.048	0.81
Congestive heart failure			-0.389	-0.576	-0.202	**<0.0001**
Coronary artery disease			-0.527	-0.635	-0.420	**<0.0001**
**Model 1**	R^2	N	Estimate	L 95% CI	U 95% CI	p-value
P02778 (CXCL10) [SD]	0.1114	5741				
FIB-4 (≥1.3)			0.251	0.179	0.322	**<0.0001**
Age [y]			0.026	0.024	0.028	**<0.0001**
Sex (Women)			0.116	0.067	0.165	**<0.0001**
**Model 2**	R^2	N	Estimate	L 95% CI	U 95% CI	p-value
P02778 (CXCL10) [SD]	0.1406	5704				
FIB-4 (≥1.3)			0.233	0.162	0.303	**<0.0001**
Age [y]			0.022	0.019	0.025	**<0.0001**
Sex (Women)			0.113	0.063	0.163	**<0.0001**
Smoking			-0.362	-0.425	-0.300	**<0.0001**
Arterial hypertension			0.033	-0.021	0.088	0.23
Diabetes mellitus			-0.067	-0.136	0.002	0.058
Obesity			0.195	0.139	0.250	**<0.0001**
Dyslipidemia			0.052	0.000	0.105	0.051
**Model 3**	R^2	N	Estimate	L 95% CI	U 95% CI	p-value
P02778 (CXCL10) [SD]	0.1387	5601				
FIB-4 (≥1.3)			0.221	0.149	0.293	**<0.0001**
Age [y]			0.022	0.019	0.025	**<0.0001**
Sex (Women)			0.113	0.062	0.163	**<0.0001**
Smoking			-0.354	-0.417	-0.291	**<0.0001**
Arterial hypertension			0.032	-0.023	0.086	0.26
Diabetes mellitus			-0.069	-0.140	0.002	0.057
Obesity			0.186	0.130	0.243	**<0.0001**
Dyslipidemia			0.046	-0.008	0.100	0.092
Congestive heart failure			0.168	-0.015	0.351	0.072
Coronary artery disease			0.061	-0.044	0.166	0.26
**Model 1**	R^2	N	Estimate	L 95% CI	U 95% CI	p-value
O15169 (AXIN1) [SD]	0.0414	5741				
FIB-4 (≥1.3)			-0.576	-0.650	-0.502	**<0.0001**
Age [y]			0.004	0.001	0.006	**0.0049**
Sex (Women)			-0.071	-0.122	-0.020	**0.0066**
**Model 2**	R^2	N	Estimate	L 95% CI	U 95% CI	p-value
O15169 (AXIN1) [SD]	0.0581	5704				
FIB-4 (≥1.3)			-0.580	-0.653	-0.506	**<0.0001**
Age [y]			-0.002	-0.004	0.001	0.24
Sex (Women)			-0.030	-0.082	0.022	0.26
Smoking			-0.057	-0.123	0.008	0.085
Arterial hypertension			0.101	0.044	0.158	**0.00049**
Diabetes mellitus			0.206	0.133	0.278	**<0.0001**
Obesity			0.066	0.008	0.124	**0.027**
Dyslipidemia			0.108	0.053	0.163	**0.00012**

Model 3	R^2	N	Estimate	L 95% CI	U 95% CI	p-value
O15169 (AXIN1) [SD]	0.0611	5601				
FIB-4 (≥1.3)			-0.558	-0.633	-0.484	**<0.0001**
Age [y]			-0.001	-0.004	0.002	0.58
Sex (Women)			-0.040	-0.093	0.013	0.14
Smoking			-0.056	-0.122	0.010	0.096
Arterial hypertension			0.103	0.046	0.160	**0.00042**
Diabetes mellitus			0.210	0.136	0.284	**<0.0001**
Obesity			0.079	0.020	0.138	**0.0084**
Dyslipidemia			0.136	0.079	0.192	**<0.0001**
Congestive heart failure			-0.050	-0.241	0.141	0.61
Coronary artery disease			-0.290	-0.400	-0.180	**<0.0001**
**Model 1**	R^2	N	Estimate	L 95% CI	U 95% CI	p-value
P49771 (Flt3L) [SD]	0.0994	5741				
FIB-4 (≥1.3)			0.221	0.150	0.293	**<0.0001**
Age [y]			0.024	0.021	0.026	**<0.0001**
Sex (Women)			0.224	0.174	0.273	**<0.0001**
**Model 2**	R^2	N	Estimate	L 95% CI	U 95% CI	p-value
P49771 (Flt3L) [SD]	0.1058	5704				
FIB-4 (≥1.3)			0.232	0.160	0.303	**<0.0001**
Age [y]			0.024	0.021	0.026	**<0.0001**
Sex (Women)			0.245	0.194	0.295	**<0.0001**
Smoking			0.188	0.124	0.252	**<0.0001**
Arterial hypertension			0.017	-0.038	0.073	0.54
Diabetes mellitus			0.032	-0.038	0.103	0.37
Obesity			0.012	-0.044	0.069	0.67
Dyslipidemia			0.040	-0.014	0.093	0.15
**Model 3**	R^2	N	Estimate	L 95% CI	U 95% CI	p-value
P49771 (Flt3L) [SD]	0.1059	5601				
FIB-4 (≥1.3)			0.225	0.152	0.298	**<0.0001**
Age [y]			0.023	0.021	0.026	**<0.0001**
Sex (Women)			0.250	0.198	0.301	**<0.0001**
Smoking			0.195	0.130	0.259	**<0.0001**
Arterial hypertension			0.022	-0.034	0.078	0.44
Diabetes mellitus			0.021	-0.051	0.093	0.57
Obesity			0.000	-0.057	0.058	0.99
Dyslipidemia			0.030	-0.025	0.085	0.28
Congestive heart failure			0.187	0.000	0.373	0.050
Coronary artery disease			0.043	-0.064	0.150	0.44

Table S7. Multivariate logistic regression for proteins (per SD) and atrial fibrillation

(Level of significance: $p < 0.05$ is considered as statistically significant (z-test), in bold)

**Model 1**	AUC	N	Odds ratio	L 95%CI	U 95%CI	p-value
Atrial fibrillation	0.7698	5672 (172 events)				
P78556 (CCL20) [SD]			1.520	1.326	1.743	**<0.0001**
Age [y]			1.090	1.070	1.110	**<0.0001**
Sex (Women)			0.522	0.378	0.721	**<0.0001**
**Model 2**	AUC	N	Odds ratio	L 95%CI	U 95%CI	p-value
Atrial fibrillation	0.7779	5635 (171 events)				
P78556 (CCL20) [SD]			1.506	1.307	1.735	**<0.0001**
Age [y]			1.082	1.061	1.104	**<0.0001**
Sex (Women)			0.543	0.390	0.756	**0.00030**
Smoking			0.824	0.507	1.340	0.43
Arterial hypertension			1.157	0.800	1.673	0.44
Diabetes mellitus			0.939	0.648	1.361	0.74
Obesity			1.099	0.786	1.536	0.58
Dyslipidemia			1.631	1.173	2.268	**0.0036**
**Model 3**	AUC	N	Odds ratio	L 95%CI	U 95%CI	p-value
Atrial fibrillation	0.8028	5543 (162 events)				
P78556 (CCL20) [SD]			1.435	1.234	1.668	**<0.0001**
Age [y]			1.071	1.049	1.093	**<0.0001**
Sex (Women)			0.538	0.378	0.766	**0.00059**
Smoking			0.912	0.557	1.494	0.71
Arterial hypertension			1.073	0.732	1.573	0.72
Diabetes mellitus			1.004	0.679	1.485	0.98
Obesity			0.985	0.691	1.405	0.93
Dyslipidemia			1.488	1.038	2.133	**0.031**
Congestive heart failure			6.239	3.691	10.545	**<0.0001**
Coronary artery disease			1.818	1.178	2.806	**0.0069**
**Model 1**	AUC	N	Odds ratio	L 95%CI	U 95%CI	p-value
Atrial fibrillation	0.7675	5672 (172 events)				
Q8NFT8 (DNER) [SD]			0.690	0.592	0.803	**<0.0001**
Age [y]			1.083	1.063	1.103	**<0.0001**
Sex (Women)			0.540	0.392	0.746	**0.00018**
**Model 2**	AUC	N	Odds ratio	L 95%CI	U 95%CI	p-value
Atrial fibrillation	0.7746	5635 (171 events)				
Q8NFT8 (DNER) [SD]			0.706	0.603	0.825	**<0.0001**
Age [y]			1.075	1.053	1.096	**<0.0001**
Sex (Women)			0.578	0.416	0.804	**0.0011**
Smoking			0.910	0.561	1.477	0.70
Arterial hypertension			1.196	0.826	1.733	0.34
Diabetes mellitus			1.152	0.797	1.663	0.45
Obesity			1.008	0.717	1.418	0.96
Dyslipidemia			1.600	1.149	2.228	**0.0054**
**Model 3**	AUC	N	Odds ratio	L 95%CI	U 95%CI	p-value

Atrial fibrillation	0.8018	5543 (162 events)				
Q8NFT8 (DNER) [SD]			0.766	0.649	0.904	**0.0017**
Age [y]			1.067	1.045	1.089	**<0.0001**
Sex (Women)			0.555	0.390	0.790	**0.0011**
Smoking			1.006	0.617	1.641	0.98
Arterial hypertension			1.100	0.750	1.615	0.63
Diabetes mellitus			1.218	0.828	1.790	0.32
Obesity			0.943	0.659	1.350	0.75
Dyslipidemia			1.468	1.023	2.107	**0.037**
Congestive heart failure			6.432	3.808	10.863	**<0.0001**
Coronary artery disease			1.599	1.032	2.479	**0.036**
**Model 1**	AUC	N	Odds ratio	L 95%CI	U 95%CI	p-value
Atrial fibrillation	0.7656	5672 (172 events)				
P02778 (CXCL10) [SD]			1.556	1.316	1.841	**<0.0001**
Age [y]			1.079	1.060	1.100	**<0.0001**
Sex (Women)			0.501	0.363	0.693	**<0.0001**
**Model 2**	AUC	N	Odds ratio	L 95%CI	U 95%CI	p-value
Atrial fibrillation	0.7747	5635 (171 events)				
P02778 (CXCL10) [SD]			1.529	1.290	1.813	**<0.0001**
Age [y]			1.072	1.051	1.094	**<0.0001**
Sex (Women)			0.536	0.386	0.747	**0.00022**
Smoking			1.035	0.637	1.682	0.89
Arterial hypertension			1.204	0.833	1.739	0.32
Diabetes mellitus			1.101	0.763	1.588	0.61
Obesity			1.101	0.788	1.538	0.57
Dyslipidemia			1.620	1.164	2.254	**0.0042**
**Model 3**	AUC	N	Odds ratio	L 95%CI	U 95%CI	p-value
Atrial fibrillation	0.8003	5543 (162 events)				
P02778 (CXCL10) [SD]			1.455	1.217	1.741	**<0.0001**
Age [y]			1.062	1.041	1.084	**<0.0001**
Sex (Women)			0.523	0.367	0.745	**0.00034**
Smoking			1.119	0.685	1.830	0.65
Arterial hypertension			1.112	0.759	1.630	0.59
Diabetes mellitus			1.171	0.797	1.721	0.42
Obesity			0.986	0.692	1.406	0.94
Dyslipidemia			1.470	1.024	2.110	**0.037**
Congestive heart failure			6.609	3.918	11.150	**<0.0001**
Coronary artery disease			1.735	1.122	2.682	**0.013**
**Model 1**	AUC	N	Odds ratio	L 95%CI	U 95%CI	p-value
Atrial fibrillation	0.7517	5672 (172 events)				
O15169 (AXIN1) [SD]			0.812	0.698	0.943	**0.0065**
Age [y]			1.092	1.072	1.112	**<0.0001**
Sex (Women)			0.536	0.389	0.740	**0.00015**
**Model 2**	AUC	N	Odds ratio	L 95%CI	U 95%CI	p-value

Atrial fibrillation	0.7662	5635 (171 events)				
O15169 (AXIN1) [SD]			0.789	0.677	0.919	**0.0023**
Age [y]			1.081	1.060	1.103	**<0.0001**
Sex (Women)			0.580	0.418	0.806	**0.0012**
Smoking			0.926	0.571	1.501	0.76
Arterial hypertension			1.231	0.852	1.778	0.27
Diabetes mellitus			1.138	0.789	1.641	0.49
Obesity			1.187	0.851	1.656	0.31
Dyslipidemia			1.704	1.226	2.369	**0.0015**
**Model 3**	AUC	N	Odds ratio	L 95%CI	U 95%CI	p-value
Atrial fibrillation	0.7944	5543 (162 events)				
O15169 (AXIN1) [SD]			0.816	0.696	0.958	**0.013**
Age [y]			1.071	1.049	1.093	**<0.0001**
Sex (Women)			0.556	0.391	0.791	**0.0011**
Smoking			1.020	0.626	1.663	0.94
Arterial hypertension			1.123	0.766	1.645	0.55
Diabetes mellitus			1.224	0.833	1.799	0.30
Obesity			1.060	0.744	1.509	0.75
Dyslipidemia			1.538	1.073	2.206	**0.019**
Congestive heart failure			6.880	4.089	11.576	**<0.0001**
Coronary artery disease			1.672	1.081	2.584	**0.021**
**Model 1**	AUC	N	Odds ratio	L 95%CI	U 95%CI	p-value
Atrial fibrillation	0.7534	5672 (172 events)				
P49771 (Flt3L) [SD]			1.226	1.044	1.440	**0.013**
Age [y]			1.088	1.067	1.108	**<0.0001**
Sex (Women)			0.517	0.375	0.714	**<0.0001**
**Model 2**	AUC	N	Odds ratio	L 95%CI	U 95%CI	p-value
Atrial fibrillation	0.7657	5635 (171 events)				
P49771 (Flt3L) [SD]			1.222	1.040	1.437	**0.015**
Age [y]			1.078	1.057	1.100	**<0.0001**
Sex (Women)			0.554	0.398	0.770	**0.00043**
Smoking			0.893	0.551	1.447	0.65
Arterial hypertension			1.212	0.840	1.750	0.30
Diabetes mellitus			1.063	0.738	1.532	0.74
Obesity			1.164	0.834	1.624	0.37
Dyslipidemia			1.658	1.193	2.305	**0.0026**
**Model 3**	AUC	N	Odds ratio	L 95%CI	U 95%CI	p-value
Atrial fibrillation	0.7963	5543 (162 events)				
P49771 (Flt3L) [SD]			1.186	1.004	1.401	**0.044**
Age [y]			1.068	1.046	1.090	**<0.0001**
Sex (Women)			0.540	0.380	0.769	**0.00062**
Smoking			0.984	0.602	1.607	0.95
Arterial hypertension			1.109	0.757	1.625	0.59
Diabetes mellitus			1.137	0.774	1.670	0.51
Obesity			1.028	0.722	1.465	0.88

Dyslipidemia	1.504	1.050	2.156	**0.026**
Congestive heart failure	6.780	4.023	11.424	**<0.0001**
Coronary artery disease	1.759	1.139	2.714	**0.011**

Table S8. Cox competing risk analysis for the proteins (by tertiles) identified in the cross-sectional analysis (event = atrial fibrillation, competing event = death)

(Level of significance: $p < 0.05$ is considered as statistically significant (z-test), in bold)

**Model 1**	Sample	HR	L95%CI	U95%CI	p-value
Atrial fibrillation ~	C-index: 0.7375, N:5396 (155 events, 107 competing events)				
P78556 (CCL20) [SD]		1.134	0.979	1.314	0.094
Age [y]		1.089	1.071	1.107	**<0.0001**
Sex (Women)		0.700	0.509	0.964	**0.029**
**Model 2**	Sample	HR	L95%CI	U95%CI	p-value
Atrial fibrillation ~	C-index: 0.7548, N:5361 (155 events, 105 competing events)				
P78556 (CCL20) [SD]		1.069	0.921	1.240	0.38
Age [y]		1.085	1.065	1.105	**<0.0001**
Sex (Women)		0.755	0.547	1.040	0.086
Smoking		1.323	0.855	2.046	0.21
Arterial hypertension		0.961	0.668	1.381	0.83
Diabetes mellitus		1.504	1.047	2.160	**0.027**
Obesity		1.358	0.971	1.900	0.074
Dyslipidemia		1.275	0.923	1.761	0.14
**Model 3**	Sample	HR	L95%CI	U95%CI	p-value
Atrial fibrillation ~	C-index: 0.7537, N:5273 (146 events, 100 competing events)				
P78556 (CCL20) [SD]		1.053	0.899	1.235	0.52
Age [y]		1.082	1.061	1.103	**<0.0001**
Sex (Women)		0.778	0.555	1.091	0.15
Smoking		1.398	0.901	2.170	0.13
Arterial hypertension		0.947	0.650	1.380	0.78
Diabetes mellitus		1.277	0.863	1.888	0.22
Obesity		1.336	0.940	1.898	0.11
Dyslipidemia		1.141	0.806	1.615	0.46
Congestive heart failure		1.860	0.892	3.876	0.098
Coronary artery disease		1.572	0.979	2.525	0.061
**Model 1**	Sample	HR	L95%CI	U95%CI	p-value
Atrial fibrillation ~	C-index: 0.7436, N:5396 (155 events, 107 competing events)				
Q8NFT8 (DNER) [SD]		0.834	0.716	0.972	**0.020**
Age [y]		1.085	1.067	1.104	**<0.0001**
Sex (Women)		0.707	0.514	0.974	**0.034**
**Model 2**	Sample	HR	L95%CI	U95%CI	p-value
Atrial fibrillation ~	C-index: 0.7589, N:5361 (155 events, 105 competing events)				
Q8NFT8 (DNER) [SD]		0.857	0.730	1.007	0.060
Age [y]		1.082	1.062	1.102	**<0.0001**

Sex (Women)		0.761	0.551	1.051	0.097
Smoking		1.336	0.865	2.064	0.19
Arterial hypertension		0.960	0.666	1.384	0.83
Diabetes mellitus		1.577	1.092	2.277	**0.015**
Obesity		1.283	0.914	1.801	0.15
Dyslipidemia		1.265	0.913	1.753	0.16
**Model 3**	Sample	HR	L95%CI	U95%CI	p-value
Atrial fibrillation ~	C-index: 0.7565, N:5273 (146 events, 100 competing events)				
Q8NFT8 (DNER) [SD]		0.899	0.761	1.063	0.21
Age [y]		1.080	1.059	1.101	**<0.0001**
Sex (Women)		0.780	0.556	1.096	0.15
Smoking		1.411	0.911	2.186	0.12
Arterial hypertension		0.949	0.650	1.386	0.79
Diabetes mellitus		1.326	0.894	1.966	0.16
Obesity		1.288	0.906	1.833	0.16
Dyslipidemia		1.140	0.803	1.618	0.46
Congestive heart failure		1.829	0.872	3.833	0.11
Coronary artery disease		1.504	0.935	2.420	0.092
**Model 1**	Sample	HR	L95%CI	U95%CI	p-value
Atrial fibrillation ~	C-index: 0.7387, N:5396 (155 events, 107 competing events)				
P02778 (CXCL10) [SD]		1.154	0.976	1.364	0.094
Age [y]		1.085	1.066	1.104	**<0.0001**
Sex (Women)		0.687	0.498	0.947	**0.022**
**Model 2**	Sample	HR	L95%CI	U95%CI	p-value
Atrial fibrillation ~	C-index: 0.7581, N:5361 (155 events, 105 competing events)				
P02778 (CXCL10) [SD]		1.158	0.983	1.365	0.079
Age [y]		1.081	1.061	1.102	**<0.0001**
Sex (Women)		0.739	0.535	1.023	0.068
Smoking		1.416	0.915	2.193	0.12
Arterial hypertension		0.967	0.672	1.393	0.86
Diabetes mellitus		1.554	1.080	2.237	**0.018**
Obesity		1.340	0.957	1.877	0.089
Dyslipidemia		1.271	0.920	1.756	0.15
**Model 3**	Sample	HR	L95%CI	U95%CI	p-value
Atrial fibrillation ~	C-index: 0.7560, N:5273 (146 events, 100 competing events)				
P02778 (CXCL10) [SD]		1.120	0.944	1.328	0.19
Age [y]		1.079	1.057	1.101	**<0.0001**
Sex (Women)		0.766	0.545	1.078	0.13
Smoking		1.474	0.949	2.290	0.084
Arterial hypertension		0.955	0.655	1.394	0.81
Diabetes mellitus		1.312	0.886	1.943	0.18
Obesity		1.322	0.929	1.882	0.12

Dyslipidemia		1.139	0.805	1.613	0.46
Congestive heart failure		1.861	0.895	3.871	0.096
Coronary artery disease		1.551	0.965	2.494	0.070
**Model 1**	Sample	HR	L95%CI	U95%CI	p-value
Atrial fibrillation ~	C-index: 0.7376, N:5396 (155 events, 107 competing events)				
O15169 (AXIN1) [SD]		0.919	0.797	1.059	0.24
Age [y]		1.089	1.071	1.108	**<0.0001**
Sex (Women)		0.703	0.511	0.968	**0.031**
**Model 2**	Sample	HR	L95%CI	U95%CI	p-value
Atrial fibrillation ~	C-index: 0.7571, N:5361 (155 events, 105 competing events)				
O15169 (AXIN1) [SD]		0.885	0.766	1.022	0.095
Age [y]		1.084	1.064	1.104	**<0.0001**
Sex (Women)		0.764	0.554	1.054	0.10
Smoking		1.348	0.871	2.085	0.18
Arterial hypertension		0.972	0.676	1.398	0.88
Diabetes mellitus		1.574	1.089	2.275	**0.016**
Obesity		1.380	0.989	1.925	0.058
Dyslipidemia		1.301	0.940	1.801	0.11
**Model 3**	Sample	HR	L95%CI	U95%CI	p-value
Atrial fibrillation ~	C-index: 0.7561, N:5273 (146 events, 100 competing events)				
O15169 (AXIN1) [SD]		0.916	0.789	1.064	0.25
Age [y]		1.081	1.061	1.103	**<0.0001**
Sex (Women)		0.784	0.559	1.101	0.16
Smoking		1.422	0.916	2.207	0.12
Arterial hypertension		0.955	0.655	1.392	0.81
Diabetes mellitus		1.326	0.891	1.972	0.16
Obesity		1.353	0.954	1.917	0.090
Dyslipidemia		1.161	0.818	1.647	0.40
Congestive heart failure		1.882	0.903	3.920	0.091
Coronary artery disease		1.533	0.946	2.485	0.083
**Model 1**	Sample	HR	L95%CI	U95%CI	p-value
Atrial fibrillation ~	C-index: 0.7360, N:5396 (155 events, 107 competing events)				
P49771 (Flt3L) [SD]		1.027	0.863	1.223	0.76
Age [y]		1.089	1.071	1.108	**<0.0001**
Sex (Women)		0.700	0.506	0.966	**0.030**
**Model 2**	Sample	HR	L95%CI	U95%CI	p-value
Atrial fibrillation ~	C-index: 0.7551, N:5361 (155 events, 105 competing events)				
P49771 (Flt3L) [SD]		1.009	0.849	1.199	0.92
Age [y]		1.085	1.065	1.105	**<0.0001**
Sex (Women)		0.758	0.547	1.050	0.095

Smoking		1.348	0.868	2.092	0.18
Arterial hypertension		0.966	0.672	1.390	0.85
Diabetes mellitus		1.531	1.064	2.203	**0.022**
Obesity		1.373	0.983	1.918	0.063
Dyslipidemia		1.282	0.927	1.773	0.13
**Model 3**	Sample	HR	L95%CI	U95%CI	p-value
Atrial fibrillation ~	C-index: 0.7545, N:5273 (146 events, 100 competing events)				
P49771 (Flt3L) [SD]		0.968	0.815	1.149	0.71
Age [y]		1.083	1.062	1.104	**<0.0001**
Sex (Women)		0.787	0.559	1.110	0.17
Smoking		1.430	0.918	2.227	0.11
Arterial hypertension		0.951	0.652	1.387	0.79
Diabetes mellitus		1.298	0.877	1.922	0.19
Obesity		1.346	0.949	1.910	0.095
Dyslipidemia		1.146	0.809	1.623	0.44
Congestive heart failure		1.895	0.907	3.960	0.089
Coronary artery disease		1.580	0.982	2.542	0.059
