# Supplementary material for: Inflammation in liver fibrosis and atrial fibrillation: A prospective population-based proteomic study
Source: JHEP Rep. 2024 Jul 18;6(10):101171. doi: 10.1016/j.jhepr.2024.101171 (PMC11460462; doi:10.1016/j.jhepr.2024.101171)
Supplement: Multimedia component 2 [file mmc2.docx]

**JHEP Reports**

**CTAT methods**

Tables for a “Complete, Transparent, Accurate and Timely account” (CTAT) are now mandatory for all revised submissions. The aim is to enhance the reproducibility of methods.

- Only include the parts relevant to your study
- Refer to the CTAT in the main text as ‘Supplementary CTAT Table’
- Do not add subheadings
- Add as many rows as needed to include all information
- Only include one item per row

**If the CTAT form is not relevant to your study, please outline the reasons why:**

| / |
| --- |

- 1. **Antibodies**

| **Name** | **Citation** | **Supplier** | **Cat no.** | **Clone no.** |
| --- | --- | --- | --- | --- |
| N/A |  |  |  |  |

- 1. **Cell lines**

| **Name** | **Citation** | **Supplier** | **Cat no.** | **Passage no.** | **Authentication test method** |
| --- | --- | --- | --- | --- | --- |
| N/A |  |  |  |  |  |

- 1. **Organisms**

| **Name** | **Citation** | **Supplier** | **Strain** | **Sex** | **Age** | **Overall n number** |
| --- | --- | --- | --- | --- | --- | --- |
| N/A |  |  |  |  |  |  |

- 1. **Sequence based reagents**

| **Name** | **Sequence** | **Supplier** |
| --- | --- | --- |
| Olink Target 96 Inflammation panel | Olink UniProt numbers available in Table S1 | Olink Proteomics, Uppsala, Sweden |

- 1. **Biological samples**

| **Description** | **Source** | **Identifier** |
| --- | --- | --- |
| EDTA-anticoagulated plasma samples | General population | N/A |

- 1. **Deposited data**

| **Name of repository** | **Identifier** | **Link** |
| --- | --- | --- |
| N/A |  |  |

- 1. **Software**

| **Software name** | **Manufacturer** | **Version** |
| --- | --- | --- |
| R | www.R-project.org | Version 4.2.1 |
| Prism | GraphPad | Version 8.4.3 |
| Mendeley Reference Manager | Elsevier | Version 2.112.2 |

- 1. **Other (*e.g*. drugs, proteins, vectors etc.)**

| N/A |  |  |
| --- | --- | --- |
|  |  |  |

- 1. **Please provide the details of the corresponding methods author for the manuscript:**

| Jörn M. Schattenberg  Department of Medicine II, Saarland University Medical Center, Kirrberger Str. 100, 66421 Homburg, Germany |
| --- |

**2.0 Please confirm for randomised controlled trials all versions of the clinical protocol are included in the submission. These will be published online as supplementary information.**

| N/A |
| --- |
