# Supplementary material for: Inflammation in liver fibrosis and atrial fibrillation: A prospective population-based proteomic study
Source: JHEP Rep. 2024 Jul 18;6(10):101171. doi: 10.1016/j.jhepr.2024.101171 (PMC11460462; doi:10.1016/j.jhepr.2024.101171)
Supplement: Multimedia component 4 [file mmc4.pdf]

Inflammation in liver fibrosis and atrial fibrillation: A prospective population-based proteomic study☆

Joost Boeckmans[1,2,3], Maurice Michel[1,2,4], Alexander Gieswinkel[5], Oliver Tüscher[6,7,8], Stavros V. Konstantinides[9,10], Jochem König[11], Thomas Münzel[12,13], Karl J. Lackner[14], Jasmin Ghaemi Kerahrodi[15], Alexander K. Schuster[16], Philipp S. Wild[5,7,9,13], Peter R. Galle[2], Jörn M. Schattenberg[1,2,4,*]

JHEP Reports **2024**. vol. 6 | 1–11

Check for updates

Background & Aims: Elevated liver stiffness has been associated with atrial fibrillation (AFib) in the general population. The mechanism underlying this association is unclear.

Methods: Participants were recruited from the general population and prospectively enrolled with follow-up for 5 years. The fibrosis-4 (FIB-4) index was used as a surrogate marker for liver fibrosis. Proteomics analysis was performed using the 92-target Olink inflammation panel. Validation was performed using the NAFLD fibrosis score (NFS), aspartate aminotransferase to platelet index (APRI), and repeat confirmation proteomics.

Results: A sample of 11,509 participants with a mean age of 54.0 ± 11.1 years, 51.3% women, and a median FIB-4 index of 0.85 (0.65/1.12), was used. The FIB-4 index was predictive for prevalent (FIB-4 index adjusted odds ratio (aOR) per SD: 1.100 with 95% CI 1.011-1.196; $p = 0.026$), but not incident AFib (log[FIB-4 index]) adjusted hazard ratio: 1.125 with 95% CI 0.943-1.342, $p = 0.19$). Elastic net regularized regression identified CCL20, DNER, and CXCL10 for prevalent AFib, and AXIN1, CXCL10, and Flt3L for the log(FIB-4 index) (per SD) as most important in common regulated proteins. The relationship between the FIB-4 index, the identified proteins, and AFib was relevant and reproduced at the 5-year follow-up for CXCL10 after adjusting for confounders (log[FIB-4 index] per SD - CXCL10 [per SD] adjusted β 0.160 with 95% CI 0.127-0.194, $p < 0.0001$; CXCL10 [per SD] - AFib aOR 1.455 with 95% CI 1.217-1.741, $p < 0.0001$), reproduced using the NFS and APRI, and corresponding to increased serum levels.

Conclusions: CXCL10 is linked to liver fibrosis, as determined by the FIB-4 index, and to prevalent AFib.

© 2024 The Author(s). Published by Elsevier B.V. on behalf of European Association for the Study of the Liver (EASL). This is an open access article under the CC BY license (http://creativecommons.org/licenses/by/4.0/).

Introduction

Metabolic dysfunction-associated steatotic liver disease (MASLD) is often associated with cardiovascular disease, including coronary artery disease, congestive heart failure, and atrial fibrillation (AFib), resulting in a high mortality rate.[1] Liver fibrosis with resulting stiffening that is associated with the more advanced stages of MASLD has been indicated as a relevant cue to prompt cardiovascular risk assessment and further investigations, including N-terminal pro–B-type natriuretic peptide (NT-proBNP) determination and electrocardiography.[2] AFib is the most common cardiac arrhythmia affecting approximately 60 million persons and its prevalence is increasing in parallel with MASLD.[3–5] The lifetime risk for AFib is 33% and largely depends on modifiable cardiovascular risk factors, including arterial hypertension, type 2 diabetes mellitus, alcohol consumption, and a sedentary lifestyle.[4]

AFib increases the risk of incident heart failure[6] and cardiovascular accidents[7] and is consequently an important condition to be treated. The treatment of AFib is, apart from cardiovascular risk management, currently based on anticoagulation therapy with vitamin K antagonists or direct-acting anticoagulants, and rate and rhythm control using pharmacological treatment or ablation. Life-threatening side effects including excessive bleeding and arrhythmias are hence inherent to current treatment modalities.[8] Mechanism-based strategies in current drug development for AFib focus on repairing the protein quality control system, DNA damage, and mitochondrial function, as well as dampening inflammatory responses.[3,9]

Patients with AFib often experience multiple comorbidities based on the presence of shared risk factors, including those involved in MASLD. Considering the lack of both safe and effective therapies for AFib, it is relevant to investigate the interrelationship between these two conditions.[3] Recent evidence indicates that liver stiffness when determined by vibration-controlled transient elastography (VCTE) rather than liver steatosis itself, is related to prevalent AFib in the general population.[10] The link between liver stiffness and AFib remains undetermined, but could lie in mechanisms involving metabolic inflammation.[11] Metabolic inflammation arising from MASLD

☆ Given their role as Editor, Jörn M. Schattenberg had no involvement in the peer-review of this article and had no access to information regarding its peer-review. Full responsibility for the editorial process for this article was delegated to the Guest Editor Ramon Bataller.

* Corresponding author. Address: Department of Medicine II, Saarland University Medical Center, Kirrberger Str. 100, 66421 Homburg, Germany. Tel.: +4968411615027.
E-mail address: Joern.Schattenberg@uks.eu (J.M. Schattenberg).

https://doi.org/10.1016/j.jhepr.2024.101171

Table 1. Baseline characteristics of the study participants stratified by the fibrosis-4 index.

Variable	Whole sample (N = 11,509)	Fibrosis-4 index <1.3 (n = 9,795)	Fibrosis-4 index ≥1.3 (n = 1,714)	*p* value
**Demographics**				
Sex (female)	51.3% (5,902)	53.5% (5,242)	38.5% (660)	<0.0001
Age, yr	54.0 ± 11.1	52.1 ± 10.5	64.8 ± 7.6	<0.0001
BMI, kg/m^2	26.6 (23.8/30.1)	26.5 (23.7/30.0)	27.3 (24.6/30.7)	<0.0001
Weight, kg	79.5 ± 16.7	79.2 ± 16.8	80.9 ± 16.1	0.00014
Height, m	1.70 ± 0.10	1.70 ± 0.10	1.70 ± 0.09	0.28
Waist, cm	94.1 ± 13.9	93.6 ± 13.9	97.3 ± 13.9	<0.0001
Protein data available	49.9% (5,741)	48.5% (4,750)	57.8% (991)	<0.0001
**Cardiovascular risk factors**				
Dyslipidaemia	34.1% (3,920)	32.2% (3,146)	45.2% (774)	<0.0001
Arterial hypertension	47.1% (5,416)	43.7% (4,274)	66.6% (1,142)	<0.0001
Smoking	19.2% (2,201)	20.7% (2,021)	10.5% (180)	<0.0001
Obesity	25.5% (2,932)	25.0% (2,443)	28.5% (489)	0.0020
Family history of myocardial infarction/stroke	22.5% (2,587)	22.7% (2,223)	21.2% (364)	0.19
Diabetes mellitus	8.9% (1,022)	7.7% (749)	15.9% (273)	<0.0001
**Comorbidities**				
Metabolic syndrome	21.9% (2,519)	20.7% (2,023)	28.9% (496)	<0.0001
Hyperuricemia	6.2% (717)	5.4% (530)	10.9% (187)	<0.0001
Coronary artery disease	4.0% (458)	3.0% (289)	10.2% (169)	<0.0001
Myocardial infarction	3.0% (339)	2.2% (216)	7.2% (123)	<0.0001
Peripheral artery disease	3.3% (371)	2.9% (280)	5.4% (91)	<0.0001
Atrial fibrillation	2.4% (275)	1.7% (168)	6.4% (107)	<0.0001
Congestive heart failure	1.3% (154)	1.0% (94)	3.5% (60)	<0.0001
Chronic kidney disease	1.0% (117)	1.0% (100)	1.0% (17)	1.00
**Liver parameters**				
Fatty liver index	45.98 ± 30.47	44.90 ± 30.49	52.14 ± 29.58	<0.0001
Fatty liver index ≥60	36.1% (4,146)	34.8% (3407)	43.1% (739)	<0.0001
Fibrosis-4 index	0.85 (0.65/1.12)	0.78 (0.62/0.98)	1.55 (1.40/1.80)	<0.0001
NAFLD fibrosis score	-2.59 ± 1.30	-2.87 ± 1.15	-1.04 ± 0.97	<0.0001
AST to platelet index	0.28 (0.23/0.35)	0.27 (0.22/0.32)	0.43 (0.36/0.54)	<0.0001
Alanine aminotransferase, U/L	32.0 (26.0/42.0)	32.0 (26.0/41.0)	34.0 (28.0/44.0)	<0.0001
Aspartate aminotransferase, U/L	25.00 (21.00/29.00)	24.00 (21.00/28.00)	29.00 (25.00/36.00)	<0.0001
Gamma-glutamyltransferase, U/L	23.00 (16.00/35.00)	23.00 (16.00/34.00)	26.00 (18.00/42.00)	<0.0001
**Other laboratory measurements**				
Cholesterol, mg/dl	219.4 ± 40.4	220.2 ± 40.2	215.1 ± 41.7	<0.0001
High-density lipoprotein, mg/dl	56.7 ± 15.4	56.8 ± 15.3	56.3 ± 16.1	0.24
Low-density lipoprotein, mg/dl	138.6 ± 35.2	139.3 ± 34.9	134.5 ± 36.7	<0.0001
Triglycerides, mg/dl	104.0 (77.0/145.0)	103.4 (77.0/145.0)	105.0 (79.0/150.0)	0.058
C-reactive protein, mg/L	1.50 (0.52/3.10)	1.50 (0.50/3.10)	1.60 (0.68/3.01)	0.23
Fibrinogen, mg/dl	321.00 (278.00/375.00)	320.00 (277.00/373.00)	332.00 (288.00/386.58)	<0.0001
Glucose, mg/L	91.0 (85.0/97.2)	90.0 (85.0/97.0)	94.0 (88.0/102.0)	<0.0001
HbA1c, %	5.50 (5.20/5.80)	5.50 (5.20/5.80)	5.60 (5.30/6.00)	<0.0001

Data presented as mean ± SD (Gaussian-distributed data), median with IQR (non-Gaussian distributed data), or as relative and absolute frequencies (categorical data); a two-sided t-test was used for comparing two Gaussian-distributed continuous variables, a Wilcoxon rank sum test for non-Gaussian distributed variables, and a chi-square for categorical data; *p* values <0.05 were considered significant.

leads to hepatic fibrosis over time and is the main cause of liver stiffening.[2,11,12] In this context, liver-derived inflammatory factors could contribute to, or even trigger AFib.[13,14] Several non-invasive tests (NITs) for advanced hepatic fibrosis have been developed in recent years, among which the fibrosis-4 (FIB-4) index has shown utility in the general population.[15]

In the present study, we investigated circulating inflammatory factors by proteomics analysis and explored targets related to both liver fibrosis, determined by the FIB-4 index, and AFib in a large population-based cohort to better understand the relationship between liver stiffening and AFib and to ultimately provide novel avenues for drug development.

Patients and methods

Study description

The Gutenberg Health Study is a prospective population-based observational cohort study underway in the Rhine-Main Region in Germany. The study has been approved by the local ethics committee and the local and federal data safety commissioners. Written informed consent was obtained from all study participants. The study protocol was in agreement with the ethical guidelines of the Declaration of Helsinki.[16]

Individuals between 35- and 74-years old from Mainz and the Mainz-Bingen district were invited to enrol to the study. The study sample consisted of 15,010 participants at baseline, enrolled between 2012 and 2017. After 5 years, data was obtained from 12,423 participants.

Exclusion criteria

Participants with cancer, participants consuming alcohol in amounts ≥20 g/day for women and ≥30 g/day for men,[17] and participants without available FIB-4 index data were excluded from the study sample.

Definitions of diseases and risk factors

Liver fibrosis was determined using the FIB-4 index according to Sterling *et al.*[18] Two clinically relevant categories were used: FIB-4 index <1.3 (low risk for advanced fibrosis) and ≥1.3 (indeterminant and high risk for advanced fibrosis). The fatty liver index (FLI) was determined according to Bedogni *et al.*[19] and was used a measure of hepatic steatosis with a cut-off of ≥60. Definitions of diseases and risk factors used throughout the text can be found in the supplemental material.

Proteomics analysis for circulating inflammatory proteins

Blood plasma was collected in ethylenediaminetetraacetic acid tubes and analysed with proximity extension assay technology (Olink Proteomics, Uppsala, Sweden) using the Inflammation panel consisting of 92 targets (full list available in Table S1). Briefly, antibody pairs containing unique DNA sequences hybridize upon binding of the specific protein, resulting in proximity extension and amplification by real-time PCR.

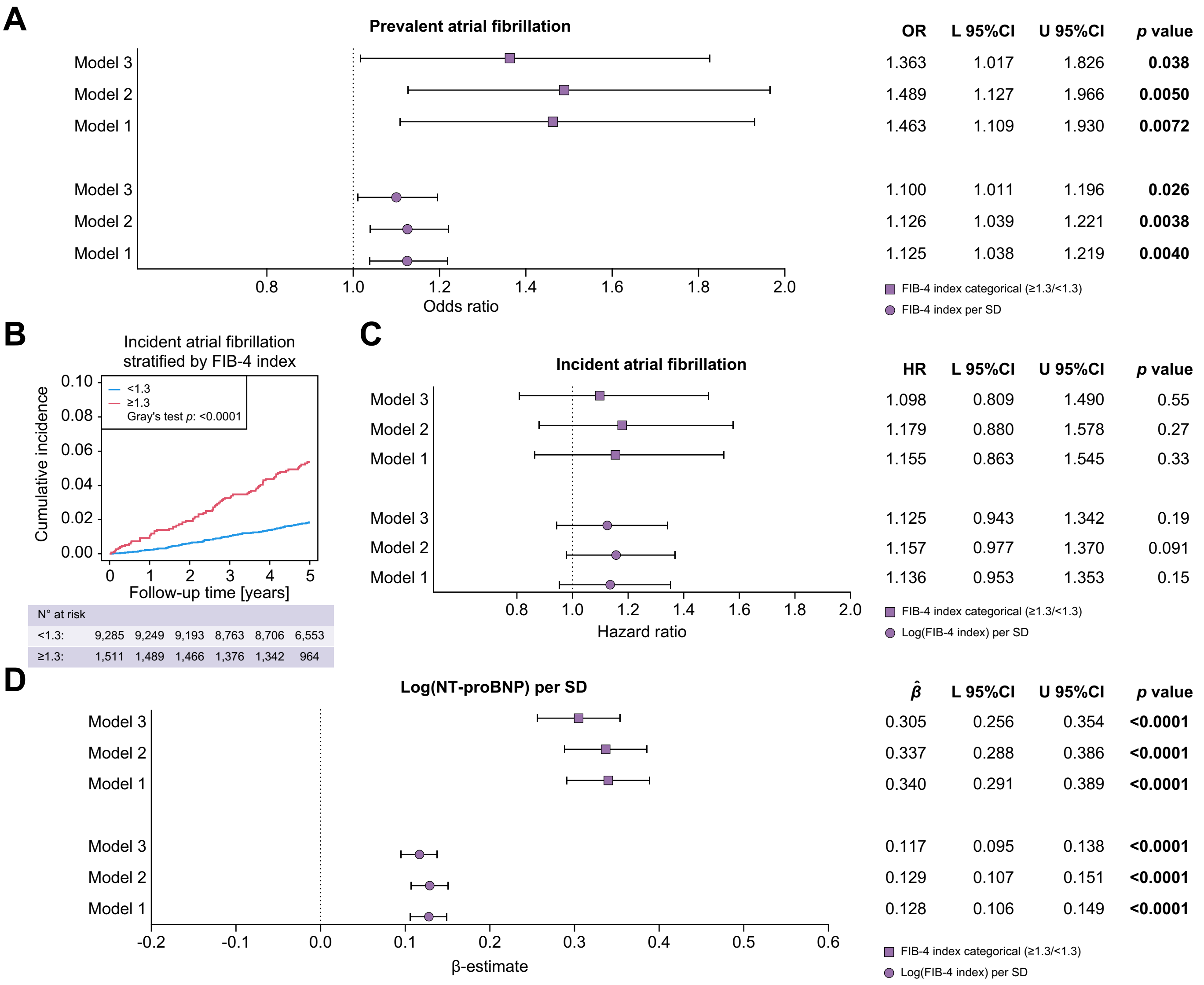

Fig. 1. Relationship between the fibrosis-4 index and atrial fibrillation. (A) Relationship between the FIB-4 index and prevalent atrial fibrillation (symbols represent odds ratios and bars represent 95% CIs). Level of significance: p <0.05 is considered as statistically significant. (multivariate logistic regression, z-test) (model 1: n = 11,395 (275 events); model 2: n = 11,335 (274 events); model 3: n = 11,186 (262 events)). (B) Cumulative incidence of atrial fibrillation based on the FIB-4 index (blue line indicates cumulative incidence of atrial fibrillation with FIB-4 index <1.3 and red line indicates cumulative incidence of atrial fibrillation with FIB-4 index ≥1.3). Level of significance: a p <0.05 is considered as statistically significant (Gray's test). (C) Relationship between the FIB-4 index and incident atrial fibrillation (symbols represent hazard ratios and bars represent 95% CIs). Level of significance: a p <0.05 is considered as statistically significant (Cox competing risk analysis, event = atrial fibrillation, competing event = death, z-test) (model 1: n = 10,796 (246 events, 166 competing events); model 2: n = 10,737 (246 events, 164 competing events); model 3: n = 10,591 (232 events, 159 competing events)). (D) Relationship between the FIB-4 index and NT-proBNP (symbols represent β-estimates and bars represent 95% CIs). Level of significance: p <0.05 is considered as statistically significant (multivariate linear regression, t-test). Model 1: adjusted for age and sex; model 2: additional adjustment for smoking, arterial hypertension, diabetes mellitus, obesity, and dyslipidaemia; model 3: additional adjustment for coronary artery disease and congestive heart failure. FIB-4, fibrosis-4; L, lower; NT-proBNP, N-terminal pro–B-type natriuretic peptide; U, upper.

	FIB-4 ≥1.3	FIB-4 <1.3	AFib	No AFib
Q9H5V8 (CDCP1)	63.6	-13.3	56.2	-2.3
Q07325 (CXCL9)	49.3	-10.3	47.4	-1.9
P02778 (CXCL10)	44.1	-9.2	58.5	-2.1
P49771 (Flt3L)	38.0	-7.9	36.8	-1.4
P78423 (CX3CL1)	36.1	-7.5	18.9	-0.8
P10147 (CCL3)	35.4	-7.4	44.7	-1.6
P14210 (HGF)	34.7	-7.2	42.5	-1.7
O00300 (OPG)	34.5	-7.2	25.3	-1.2
O15444 (CCL25)	34.3	-7.2	46.9	-2.0
Q07011 (TNFRSF9)	32.2	-6.7	41.2	-1.5
P42702 (LIF R)	31.2	-6.5	26.3	-1.2
P28325 (CST5)	30.2	-6.3	24.7	-0.8
Q13261 (IL 15RA)	29.0	-6.0	36.9	-1.3
P10145 (IL8)	28.9	-6.0	39.6	-1.6
P13500 (MCP 1)	27.1	-5.6	16.9	-0.7
P80098 (MCP 3)	26.6	-5.6	35.4	-1.3
P51671 (CCL11)	24.7	-5.2	26.7	-0.8
P29460 (IL 12B)	22.5	-4.7	28.9	-1.1
P13236 (CCL4)	22.0	-4.6	26.0	-0.8
P05231 (IL6)	21.3	-4.4	44.3	-1.8
P00749 (uPA)	20.9	-4.4	0.8	0.1
Q13291 (SLAMF1)	20.5	-4.3	18.2	-0.6
Q9NRJ3 (CCL28)	18.2	-3.8	1.4	0.0
Q13478 (IL 18R1)	18.1	-3.8	27.2	-1.3
P39905 (GDNF)	17.9	-3.7	5.4	-0.1
Q08334 (IL 10RB)	17.6	-3.7	19.6	-0.9
Q9NSA1 (FGF 21)	17.2	-3.6	36.5	-1.8
Q14116 (IL18)	16.5	-3.5	28.4	-1.2
P78556 (CCL20)	15.8	-3.3	53.1	-2.1
Q9NZQ7 (PD L1)	15.2	-3.2	23.5	-0.9
P01135 (TGF alpha)	14.8	-3.1	27.5	-1.0
P30203 (CD6)	13.2	-2.8	17.8	-0.6
Q99616 (MCP 4)	12.8	-2.7	19.7	-0.6
P22301 (IL10)	12.8	-2.7	14.8	-0.3
Q9GZV9 (FGF 23)	12.6	-2.6	54.0	-2.2
P80075 (MCP 2)	12.5	-2.6	12.4	-0.5
Q9P0M4 (IL 17C)	12.4	-2.6	28.9	-1.1
P09603 (CSF 1)	11.9	-2.5	14.4	-0.6
P01375 (TNF)	11.8	-2.5	23.4	-0.6
P80511 (EN RAGE)	11.7	-2.4	40.7	-1.7
O95750 (FGF 19)	11.7	-2.5	10.4	-0.5
P12034 (FGF 5)	10.8	-2.2	8.3	-0.1
P00813 (ADA)	10.1	-2.1	8.3	0.0
Q14790 (CASP 8)	9.9	-2.1	35.0	-1.3
P01579 (IFN gamma)	9.4	-2.0	20.3	-0.4
Q99731 (CCL19)	8.1	-1.7	26.9	-1.0
P15692 (VEGFA)	7.7	-1.6	37.4	-1.5
P01732 (CD8A)	6.6	-1.4	5.1	-0.1
O14625 (CXCL11)	4.8	-1.0	9.4	-0.3
P09238 (MMP 10)	4.1	-0.9	10.2	-0.5
Q16552 (IL 17A)	3.9	-0.8	10.4	-0.4
P01138 (Beta NGF)	2.4	-0.5	-12.1	0.4
P06127 (CD5)	0.8	-0.2	4.4	-0.2
P55773 (CCL23)	-0.5	0.1	6.7	-0.4
P21583 (SCF)	-0.8	0.2	-18.7	0.7
P25942 (CD40)	-2.1	0.4	15.8	-0.7
P01374 (TNFB)	-3.5	0.7	-2.5	0.3
Q9BZW8 (CD244)	-4.4	0.9	7.5	-0.3
P01137 (LAP TGF beta 1)	-5.1	1.1	15.7	-0.5
P15018 (LIF)	-5.2	1.1	0.2	0.3
Q99748 (NRTN)	-5.6	1.2	-33.4	1.2
Q969D9 (TSLP)	-6.3	1.3	-17.4	0.8
Q9UHF4 (IL 20RA)	-6.4	1.3	-23.2	0.8
Q13007 (IL 24)	-7.0	1.5	-9.3	0.3
P05112 (IL4)	-7.7	1.6	-8.6	0.3
O95760 (IL33)	-7.9	1.6	-19.8	0.9
P14784 (IL 2RB)	-8.0	1.7	-22.0	1.0
Q13651 (IL 10RA)	-8.1	1.7	-7.1	0.3
O43508 (TWEAK)	-8.2	1.7	-32.5	1.4
Q13541 (X4E BP1)	-8.5	1.8	34.4	-1.3
P03956 (MMP 1)	-9.3	1.9	19.1	-0.7
Q5T4W7 (ARTN)	-9.8	2.0	-12.4	0.6
P35225 (IL13)	-10.1	2.1	-21.1	1.0
P80162 (CXCL6)	-10.2	2.1	2.9	0.0
Q9NYY1 (IL 20)	-11.9	2.5	-24.6	1.0
Q8N6P7 (IL 22 RA1)	-12.2	2.5	-5.0	0.2
P05113 (IL5)	-12.9	2.7	-10.5	0.6
P13725 (OSM)	-14.2	3.0	3.1	0.0
P20783 (NT 3)	-14.4	3.0	-6.1	0.4
P50591 (TRAIL)	-15.6	3.3	-11.1	0.7
Q8NFT8 (DNER)	-16.3	3.4	-51.0	2.3
P01583 (IL 1 alpha)	-16.5	3.4	-20.6	0.8
O43557 (TNFSF14)	-16.6	3.5	-1.7	0.1
O14788 (TRANCE)	-18.2	3.8	6.7	0.1
P60568 (IL2)	-18.8	3.9	-37.4	1.6
P42830 (CXCL5)	-27.1	5.7	-12.2	0.3
P13232 (IL7)	-28.9	6.0	-2.1	0.1
P09341 (CXCL1)	-29.5	6.2	-5.7	0.2
P50225 (ST1A1)	-31.7	6.6	6.7	-0.1
O95630 (STAMBP)	-36.3	7.6	-4.0	0.1
Q8IXJ6 (SIRT2)	-43.0	9.0	-10.1	0.4
O15169 (AXIN1)	-43.2	9.0	-25.5	0.8

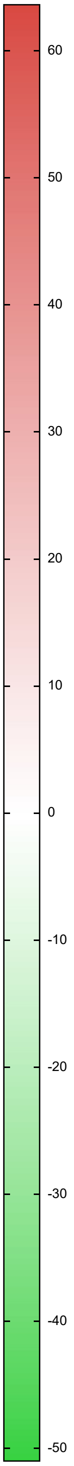

Fig. 2. Protein expressions in %SD of the overall mean in study participants with a Fibrosis-4 Index ≥1.3 and atrial fibrillation (sorted by Fibrosis-4 Index). Colour scale indicates protein expression rate; green, lower expression, and red, higher expression, compared with the overall mean). AFib, atrial fibrillation; FIB-4, fibrosis-4.

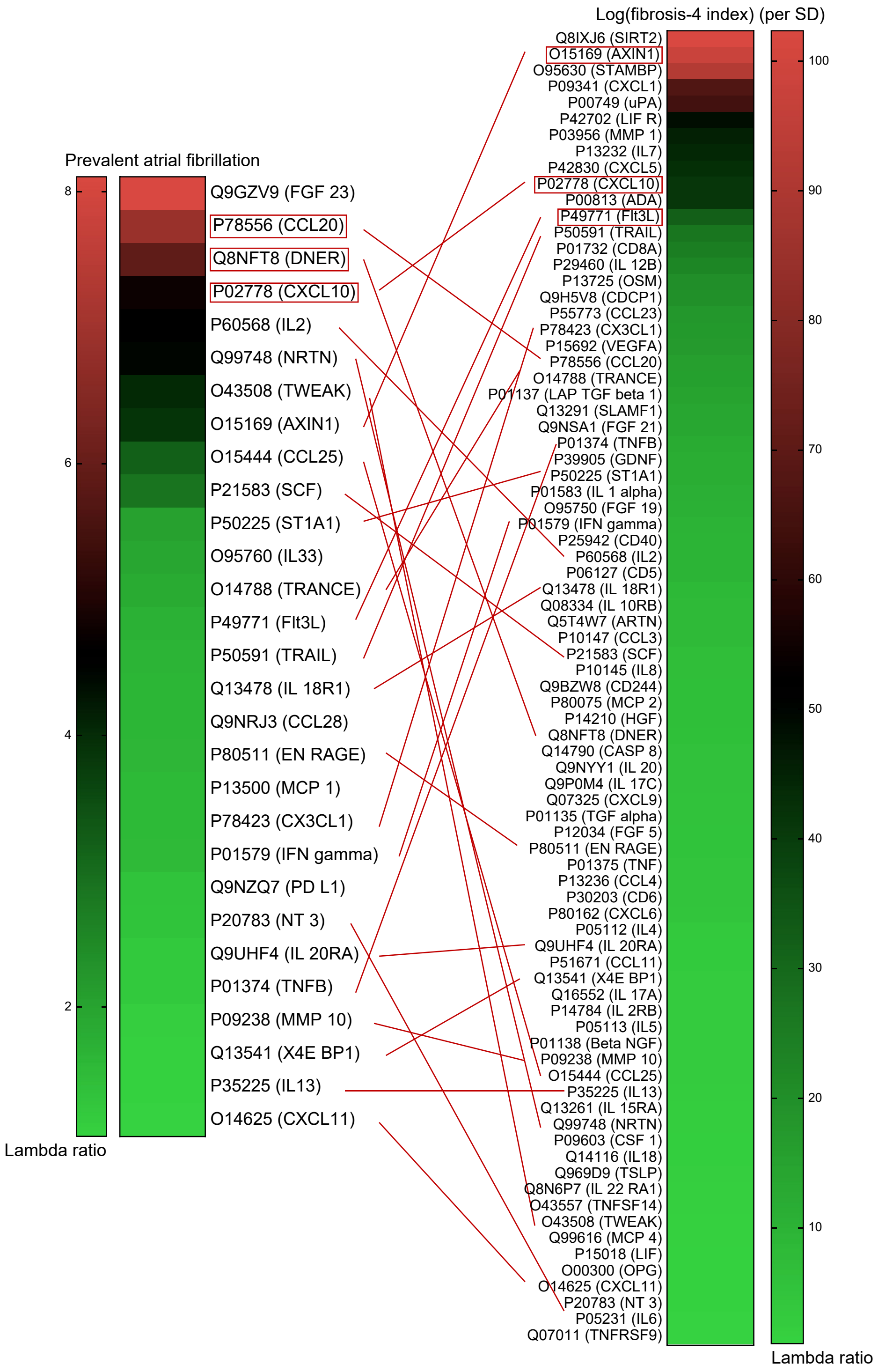

Fig. 3. Selection of the most important proteins related to liver fibrosis and atrial fibrillation. Elastic net regularized regression was used to identify the most relevant circulating proteins in atrial fibrillation and liver fibrosis based on the FIB-4 index on a continuous scale (atrial fibrillation: 10 fold cross-validation AUC = 0.7867, (simple AUC = 0.8202), minimal lambda = 0.004 (10 fold-cross validation), n = 5,672, events = 172, number of proteins = 92, adjusted for age (SD) and sex; log(FIB-4 index): 10-fold cross-validation R^2 = 0.6173, (simple R^2 = 0.6302), minimal lambda = 0.005 (10-fold-cross validation), m = 5,741, events = 155, number of proteins = 92, (adjusted for age [SD] and sex). Colour scale indicates the lambda ratios within the spectrum of obtained results, green = lower, and red = higher lambda ratio (Lambda ratios prevalent arial fibrillation: CCL20: 6.868, DNER: 5.872, CXCL10: 4.750; log[fibrosis-4 index]: AXIN1: 97.731, CXCL10: 41.078, FIT3L: 32.026). AUC, area under the curve; AXIN1, Axis inhibition protein 1; CCL20, C-C motif chemokine ligand 20; CXCL10, C-X-C motif chemokine ligand 10; DNER, Delta and Notch-like epidermal growth factor-related receptor; Flt3L: Fms related receptor tyrosine kinase 3 ligand.

Validation of results

The NAFLD fibrosis score (NFS), determined according to Angulo *et al.*[20] and the AST to platelet index (APRI), determined according to Wai *et al.*[21] using aspartate aminotransferase (AST) cut-offs of 35 U/L and 31 U/L for men and women, respectively,[22] were used to validate results obtained with the FIB-4 index. Validation of the proteomics results was performed by running a repeated Olink inflammation analysis at the 5-year follow-up.

Statistical analyses

Continuous normally distributed data are presented as mean ± SD and continuous skewed data were presented as median with IQR. Discrete data are described using absolute and relative frequencies. Multivariate logistic regression were used to investigate the relationship between NITs for liver fibrosis and AFib. Cox competing risk analysis was used to adjust for potential confounders in the longitudinal analysis. Multivariate logistic and linear regression for the cross-sectional analyses, and Cox competing risk analysis for longitudinal analyses, were used to investigate the relationships between the NITs for liver fibrosis, AFib, and systemic proteins with adjustments for potential confounders. Elastic net regularized regression models with 10-fold cross-validation were employed to select the most relevant in common modulated inflammatory mediators, with adjustment for age and sex. For that purpose, the λ ratio was used as a scale-invariant measure of predictive robustness to rank proteins according to their relevance. Analyses were performed using R (www.R-project.org, v.4.2.1) and graphs were prepared using GraphPad Prism (v.8.4.3).

Results

Baseline characteristics of the study participants

Of the 15,010 enrolled participants, 3501 participants were excluded resulting in a study sample of 11,509 individuals (Fig. S1). The study participants had a mean age of 54.0 ± 11.1 years and consisted of 51.3% women (Table 1). The median FIB-4 index was 0.85 (0.65/1.12) and 36.1% had hepatic steatosis as determined by a fatty liver index ≥60. Cardiovascular risk factors were more often present in participants with a FIB-4 index ≥1.3 compared with persons with a FIB-4 index <1.3, including dyslipidaemia (45.2% *vs*. 32.2%), arterial hypertension (66.6% *vs*. 43.7%), obesity (28.5% *vs*. 25.0%), and diabetes mellitus (15.9% *vs*. 7.7%). Consequently, participants with a FIB-index ≥1.3 presented with the metabolic syndrome more frequently than participants with a FIB-4 index <1.3 (28.9% *vs*. 20.7%). In addition, AFib was present in 6.4% of the persons with a FIB-4 index ≥1.3 compared with 1.7% in participants with a FIB-index <1.3. Other cardiovascular diseases were also more prevalent in participants with a FIB-4 index ≥1.3 compared with those having a FIB-4 index <1.3, among which were congestive heart failure (3.5% *vs*. 1.0%), coronary artery disease (10.2% *vs*. 3.0%), and peripheral artery disease (5.4% *vs*. 2.9%). On the contrary, participants with a FIB-4 index <1.3 were more often smokers compared with participants with a FIB-4 index ≥1.3 (20.7% *vs*. 10.5%).

Relationship between liver fibrosis and atrial fibrillation

The relationships between liver fibrosis determined by the FIB-4 index and prevalent and incident AFib were investigated employing three additive models (model 1: adjusted for age and sex; model 2: additional adjustment for smoking, arterial hypertension, diabetes mellitus, obesity, and dyslipidaemia; model 3: additional adjustment for coronary artery disease and congestive heart failure) using multivariate logistic regression and Cox competing risk analysis, respectively. The FIB-4 index used both as a continuous and a categorical variable was significantly related to prevalent AFib in all three models (model 3: FIB-4 index per SD: odds ratio (OR) 1.100 with 95% CI 1.011-1.196, p = 0.026; FIB-4 index categorical (≥1.3/<1.3): OR 1.363 with 95% CI 1.017-1.826, p = 0.038) (Fig. 1A). Age, sex, dyslipidaemia, congestive heart failure, and coronary artery disease were factors that significantly influenced the relationship between the FIB-4 index and prevalent AFib (Table S2A and B). The relationship between liver fibrosis and prevalent Afib was replicated using NFS (model 3: NFS per SD: OR 1.253 with 95% CI 1.040-1.509, p = 0.017) and APRI (model 3: log[APRI] per SD: OR 1.158 with 95% CI 1.026-1.308, p = 0.018) (Fig. S2). The data on the FIB-4 index, NFS, and APRI were reproduced through repeated measurements in the same cohort after 5 years (Fig. S3A and B; Table S3 shows the study participant characteristics at the 5-year follow-up). Although a FIB-4 index ≥1.3 predicted incident AFib in an unadjusted model (Gray's test p <0.0001) (Fig. 1B), it was not the case when confounders were taken into account (model 3: log[FIB-4 index] per SD: hazard ratio [HR] 1.125 with 95% CI 0.943-1.342, p = 0.19; FIB-4 index categorical [≥1.3/<1.3]: HR 1.098 with 95% CI 0.809-1.490, p = 0.55) (Fig. 1C). Age, sex, and congestive heart failure were factors that influenced the relationship between the FIB-4 index and incident AFib (Table S4A and B). Nonetheless, the FIB-4 index related to log(NT-proBNP) levels per SD (model 3: β-estimate log[FIB-4 index] per SD: 0.117 with 95% CI 0.095-0.138, p <0.0001; β-estimate FIB-4 index categorical [≥1.3/<1.3]: 0.305 with 95% CI 0.256-0.354, p <0.0001) (Fig. 1D and Table S5A and B), which is an independent marker for prevalent and incident AFib,[23] which was also reproduced in the cohort (Fig. S4). Although the FLI was also related to prevalent AFib (Fig. S5A), it was inversely related to NT-proBNP levels (Fig. S5B).

Proteomics analysis based on the fibrosis-4 index and atrial fibrillation

As the FIB-4 index was related to prevalent, but not incident AFib, we searched for inflammatory mediators connecting liver fibrosis to prevalent AFib. Fig. 2 shows the relative changes in the %SD of the overall mean of 92 systemic protein levels based on the FIB-4 index (≥1.3/<1.3) and AFib. The top three upregulated proteins in participants with a FIB-4 index of at least 1.3 were CUB domain containing protein 1 (CDCP1) (+63.6%), C-X-C motif chemokine ligand (CXCL) 9 (+49.3%), and CXCL10 (+44.1%), whereas the top three downregulated proteins were AXIN1 (-43.2%), Sirtuin 2 (SIRT2) (-43.0%), and STAM binding protein (STAMBP) (-36.3%). The top three elevated proteins in participants with AFib were CXCL10 (+58.5%), CDCP1 (+56.2%) and fibroblast growth factor 23 (FGF23) (+54.0%) and the top three down-regulated proteins were delta and notch-like epidermal growth factor-related receptor (DNER) (-51.0%), IL-2 (-37.4%), and neurturin (NRTN) (-33.4%) (Fig. S6).

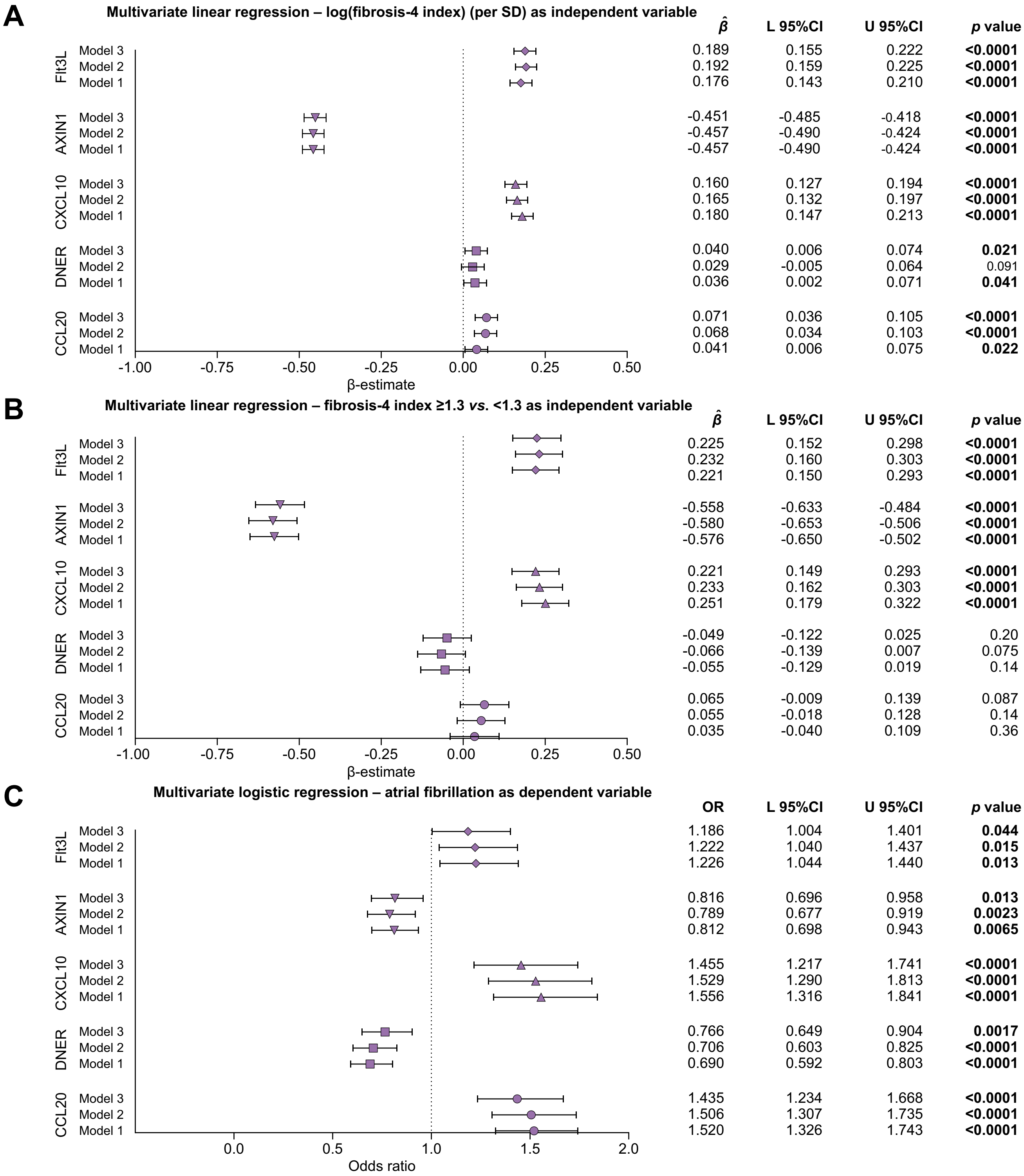

A

		$\hat{\beta}$	L 95%CI	U 95%CI	*p* value
Flt3L	Model 3	0.189	0.155	0.222	**<0.0001**
	Model 2	0.192	0.159	0.225	**<0.0001**
	Model 1	0.176	0.143	0.210	**<0.0001**
AXIN1	Model 3	-0.451	-0.485	-0.418	**<0.0001**
	Model 2	-0.457	-0.490	-0.424	**<0.0001**
	Model 1	-0.457	-0.490	-0.424	**<0.0001**
CXCL10	Model 3	0.160	0.127	0.194	**<0.0001**
	Model 2	0.165	0.132	0.197	**<0.0001**
	Model 1	0.180	0.147	0.213	**<0.0001**
DNER	Model 3	0.040	0.006	0.074	**0.021**
	Model 2	0.029	-0.005	0.064	0.091
	Model 1	0.036	0.002	0.071	**0.041**
CCL20	Model 3	0.071	0.036	0.105	**<0.0001**
	Model 2	0.068	0.034	0.103	**<0.0001**
	Model 1	0.041	0.006	0.075	**0.022**

B

		$\hat{\beta}$	L 95%CI	U 95%CI	*p* value
Flt3L	Model 3	0.225	0.152	0.298	**<0.0001**
	Model 2	0.232	0.160	0.303	**<0.0001**
	Model 1	0.221	0.150	0.293	**<0.0001**
AXIN1	Model 3	-0.558	-0.633	-0.484	**<0.0001**
	Model 2	-0.580	-0.653	-0.506	**<0.0001**
	Model 1	-0.576	-0.650	-0.502	**<0.0001**
CXCL10	Model 3	0.221	0.149	0.293	**<0.0001**
	Model 2	0.233	0.162	0.303	**<0.0001**
	Model 1	0.251	0.179	0.322	**<0.0001**
DNER	Model 3	-0.049	-0.122	0.025	0.20
	Model 2	-0.066	-0.139	0.007	0.075
	Model 1	-0.055	-0.129	0.019	0.14
CCL20	Model 3	0.065	-0.009	0.139	0.087
	Model 2	0.055	-0.018	0.128	0.14
	Model 1	0.035	-0.040	0.109	0.36

C

		OR	L 95%CI	U 95%CI	*p* value
Flt3L	Model 3	1.186	1.004	1.401	**0.044**
	Model 2	1.222	1.040	1.437	**0.015**
	Model 1	1.226	1.044	1.440	**0.013**
AXIN1	Model 3	0.816	0.696	0.958	**0.013**
	Model 2	0.789	0.677	0.919	**0.0023**
	Model 1	0.812	0.698	0.943	**0.0065**
CXCL10	Model 3	1.455	1.217	1.741	**<0.0001**
	Model 2	1.529	1.290	1.813	**<0.0001**
	Model 1	1.556	1.316	1.841	**<0.0001**
DNER	Model 3	0.766	0.649	0.904	**0.0017**
	Model 2	0.706	0.603	0.825	**<0.0001**
	Model 1	0.690	0.592	0.803	**<0.0001**
CCL20	Model 3	1.435	1.234	1.668	**<0.0001**
	Model 2	1.506	1.307	1.735	**<0.0001**
	Model 1	1.520	1.326	1.743	**<0.0001**

Fig. 4. Relationship between circulating inflammatory proteins with the Fibrosis-4 Index and atrial fibrillation. (A) Relationship between the log(FIB-4 index) (per SD) and circulating proteins (symbols represent β-estimates and bars represent 95% CIs). Level of significance: p <0.05 is considered as statistically significant (multivariate linear regression, t-test) (model 1: n = 5,741; model 2: n = 5,704; model 3: n = 5,601). (B) Relationship between the FIB-4 index (categorical ≥1.3 *vs.* <1.3) and circulating proteins (symbols represent β-estimates and bars represent 95% CIs). Level of significance: p <0.05 is considered as statistically significant (multivariate linear regression, t-test) (model 1: n = 5,741; model 2: n = 5,704; model 3: n = 5,601). (C) Relationship between circulating proteins and atrial fibrillation (symbols represent odds ratios and bars represent 95% CIs). Level of significance: p <0.05 is considered as statistically significant (multivariate logistic regression, z-test) (model 1: n = 5,672 [172 events]; model 2: n = 5,635 [171 events]; model 3: n = 5,543 [162 events]). Model 1: adjusted for age and sex; model 2: additional adjustment for smoking, arterial hypertension, diabetes mellitus, obesity, and dyslipidaemia; model 3: additional adjustment for coronary artery disease and congestive heart failure. AXIN1, Axis inhibition protein 1; CCL20, C-C motif chemokine ligand 20; CXCL10, C-X-C motif chemokine ligand 10; DNER, Delta and Notch-like epidermal growth factor-related receptor; FIB-4, fibrosis-4; Flt3L: Fms related receptor tyrosine kinase 3 ligand; L, lower; OR, odds ratio; U, upper.

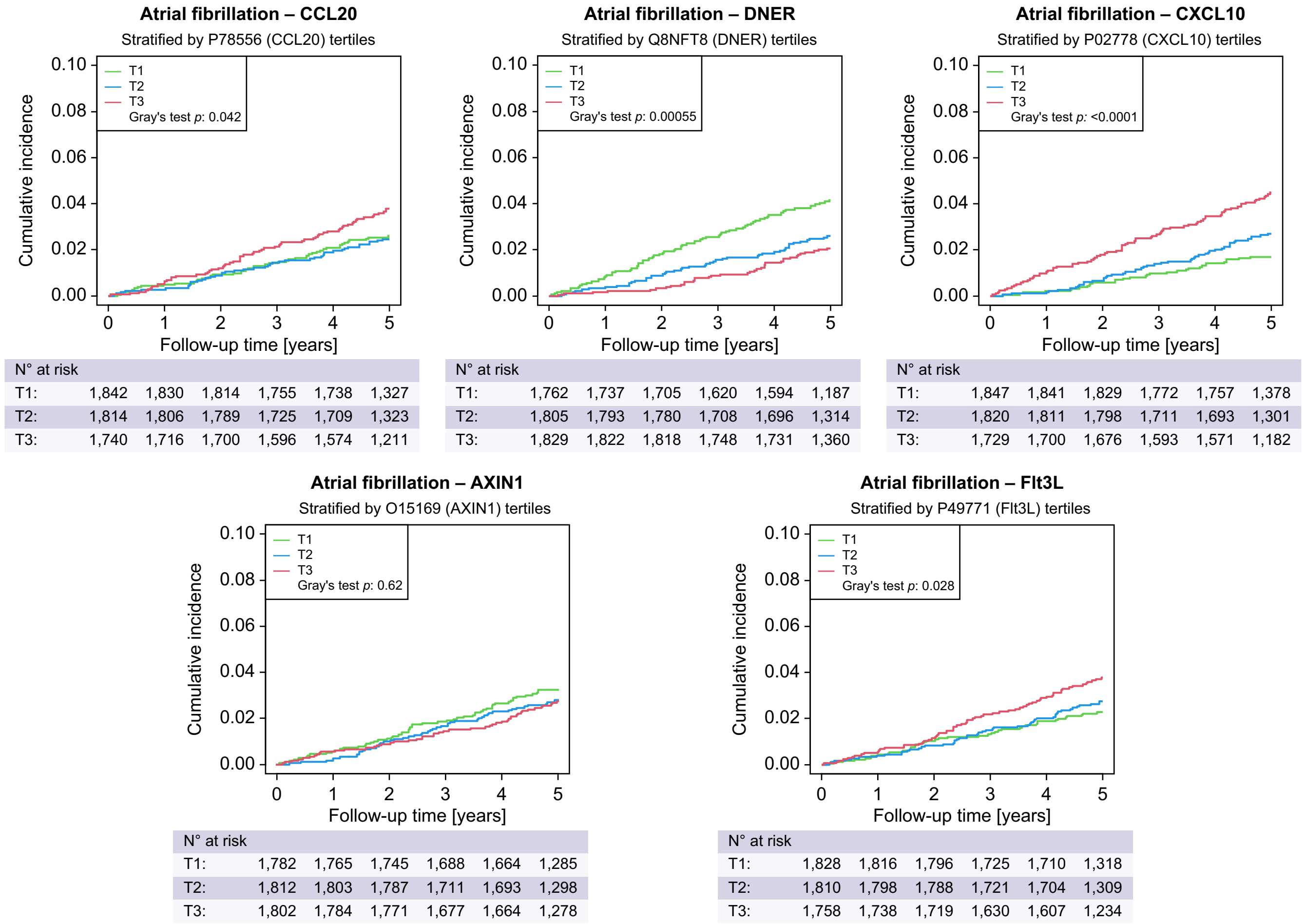

Fig. 5. Cumulative incidence of atrial fibrillation based on tertiles of proteins. Green line indicates the first tertile, blue line indicates the second tertile, and red line indicates the third tertile of protein expression. Level of significance: p <0.05 is considered as statistically significant (Gray's test). AXIN1, Axis inhibition protein 1; CCL20, C-C motif chemokine ligand 20; CXCL10, C-X-C motif chemokine ligand 10; DNER, Delta and Notch-like epidermal growth factor-related receptor; Flt3L: Fms related receptor tyrosine kinase 3 ligand.

To find the most relevant and important proteins that could link liver fibrosis to AFib, Elastic net regularized regression analyses were performed with adjustments for age and sex (Fig. 3). As the lower FIB-4 index cut-off of 1.3 performs best in persons ≤65 years,[24] and the median age of participants with a FIB-4 index ≥1.3 was 64.8 ± 7.6 years, we used the FIB-4 index on a continuous scale in the protein selection process. The top-three in common modulated proteins were selected for both AFib and log(FIB-4 index) per SD. For AFib, CCL20, DNER, and CXCL10 were selected and for the FIB-4 index, AXIN1, CXCL10, and Fms related receptor tyrosine kinase 3 ligand (Flt3L) were selected, resulting in five unique proteins for further analysis. AXIN1, CXCL10, and Flt3L also had the highest λ ratios based on a FIB-4 index ≥1.3, as common modulated proteins with AFib (Fig. S7).

CXCL10 was an inflammatory nexus between liver fibrosis and prevalent atrial fibrillation

Multivariate linear and logistic regression analyses were used to investigate the exact relationships between the FIB-4 index and the selected proteins, and the selected proteins and AFib, respectively.

When adjusted for age, sex, smoking, arterial hypertension, diabetes mellitus, obesity, dyslipidaemia, coronary artery disease, and congestive heart failure, the standardized log(FIB-4 index) was significantly related with Flt3L (β-estimate 0.189 with 95% CI 0.155-0.222, p <0.0001), AXIN1 (β-estimate -0.451 with 95% CI -0.485 to -0.418, p <0.0001), CXCL10 (β-estimate 0.160 with 95% CI 0.127-0.194, p <0.0001), DNER (β-estimate 0.040 with 95% CI 0.006-0.074, p = 0.021), and CCL20 (β-estimate 0.071 with 95% CI 0.036; 0.105, p <0.0001) (Fig. 4A). When performing the same regression analyses with the FIB-4 index as a categorical variable (≥1.3/<1.3), the relationships with DNER and CCL20 disappeared (Fig. 4B). The relationship between log(FIB-4 index) per SD with Flt3L was influenced by sex, age, and smoking, whereas the relationship with AXIN1 was additionally impacted by arterial hypertension, diabetes mellitus, obesity, dyslipidaemia, and coronary artery disease. The relationship with CXCL10 was influenced by sex, age, smoking, obesity, and dyslipidaemia (Table S6A and B).

Logistic regression analyses with the inflammatory proteins as independent variables and AFib as the dependent variable adjusted for the same set of possible confounders as done for the linear regression analyses with the FIB-4 index showed significant relationships for FltL3 (OR 1.186 with 95% CI 1.004-

1.401, p = 0.044), AXIN1 (OR 0.816 with 95% CI 0.696-0.958, p = 0.013), CXCL10 (OR 1.455 with 95% CI 1.217-1.741, p <0.0001), DNER (OR 0.766 with 95% CI 0.649-0.904, p = 0.0017), and CCL20 (OR 1.435 with 95% CI 1.234-1.668, p <0.0001) (Fig. 4C). Congestive heart failure was the most important confounder among all investigated proteins in relation to AFib (Table S7).

Multivariate linear regressions with NFS and APRI confirmed an independent relationship of liver fibrosis with Flt3L, AXIN1, and CXCL10 (Fig. S8). Identical multivariate linear regressions using repeated measurements after 5 years validated the relationships between the FIB-4 index, NFS, and APRI, and Flt3L, AXIN1, and CXCL10 (Fig. S9). In contrast, only CXCL10 was validated in multivariate logistic regression analysis as a predictor of prevalent AFib (Fig. S10). Consequently, CXCL10 was identified as the most important inflammatory protein at the interface between liver fibrosis and prevalent AFib. Additional analyses for incident AFib showed that CXCL10 (by tertiles) adds to the risk of incident AFib in a crude competing risk analysis (Gray's test p <0.0001) (Fig. 5), but not when adjusted for confounders (Table S8).

Discussion

Elevated liver stiffness determined by the VCTE was recently found to be associated with AFib in the general population (OR 1.09 per kPa, 95% CI 1.03-1.16).[10] We hypothesised that liver-related systemic inflammation against a background of liver fibrosis induced from MASLD could lay at the basis of incident and prevalent AFib.

In this large prospective population-based Western-European cohort, we found an independent relationship between the FIB-4 index and prevalent AFib, which is in line with the findings of a South-Korean study with 74,946 patients with MASLD in which an adjusted OR of 2.255 (with 95% CI 1.744-2.915) for the FIB-4 index (used as categorical variable with <1.30, 1.3–2.67, and >2.67 cut-off values) and prevalent AFib was reported.[25] The relationship between liver stiffness and the FIB-4 index and the risk of incident AFib is less clear. A Japanese study including 37,892 unemployed or retired participants aged ≥40 years with a median follow-up period of 5 years reported an adjusted HR of 1.70 (95% CI 1.29-2.23) of developing AFib for subjects with a FIB-4 index in the highest quartile compared with the lowest quartile.[26] Although we found a significant predictive role of the FIB-4 index for incident AFib in the crude risk analysis, it was not the case when the analysis was adjusted for age and sex and an additional set of well-established confounders. In contrast, the relationship between the FIB-4 index and NT-proBNP levels in the fully-adjusted model supported the role of liver fibrosis in AFib, as NT-proBNP is even a better marker for prevalent and incident AFib than for heart failure in stable outpatients.[23]

As mechanism-based treatments for AFib and MASLD are gaining momentum in drug development, and it is well known that AFib can have its basis in systemic inflammation[9,27–29] we consequently searched for inflammatory proteins that could link liver fibrosis with prevalent AFib.[3]

In the current analysis, we identified increased CXCL10 levels as an inflammatory nexus between liver fibrosis and prevalent AFib. The association of this circulating protein was preserved after correcting for a broad set of potential confounders in multivariate regression models and was reproduced at the 5-year follow-up.

CXCL10 can bind onto CXCR3 and is a chemoattractant produced by both immune and non-immune cells with pleiotropic functions including the chemoattraction of activated T-cells, macrophages, monocytes, and natural killer cells.[30] CXCL10 has reported to be elevated in patients experiencing AFib[31] and other cardiovascular diseases including atherosclerosis[32] and myocardial infarction.[33] In the liver, extracellular vesicles containing CXCL10 are released by hepatocytes mediated by mixed lineage kinase 3 in response to lipotoxicity, which in turn function as an attractant for macrophages.[34] Hence, CXCL10 has been identified as a crucial protein in the pathogenesis of MASH, together with other pro-inflammatory cytokines (monocyte chemoattractant protein 1, IL-1β, and tumour necrosis factor-α), and mechanisms including lipogenesis, and oxidative stress, and also correlating with lobular inflammation.[35]

Mechanistically in relation to AFib, myocardial infarction-associated transcript (MIAT), which is increased in serum extracellular vesicles of patients with AFib, can bind to miR-485-5p to decrease its inhibitory effect on CXCL10, resulting in atrial myocyte fibrosis, inflammation, and oxidative stress in both *in vitro* and *in vivo* experimental models.[36] Apart from a direct action of CXCL10 on the myocardium, CXCL10 can also promote AFib through its pro-atherogenic properties since subclinical atherosclerosis is an independent risk factor for developing AFib.[32,37] Consequently, CXCL10 can act through different mechanisms in the pathophysiology of AFib, which could at least partly be attributed to underlying liver fibrosis. Nonetheless, CXCL10 levels decrease after cryoballoon and radiofrequency balloon ablation, suggesting that CXCL10 secretion is also mediated by AFib itself.[38] Furthermore, CXCL10 levels are also increased in patients with non-fibrotic MASLD,[39] positioning it as a potential prognostic marker and therapeutic target for progressive MASLD-related cardiovascular disease, including AFib.

Four additional proteins identified as possible connections between liver fibrosis and AFib in this study were AXIN1, Flt3L, DNER, and CCL20. While AXIN1 potently negatively correlated with NITs for liver fibrosis, its relationship with AFib was not reproduced at the 5-year follow-up period. Nonetheless, the link between AXIN1 and AFib has been earlier elegantly identified through exosome sequencing in the serum of patients with AFib. Circulating exosomal miRNA-124-3p was increased in the plasma of patients with AFib, whereas AXIN1 appeared to be its target in a luciferase assay. In addition, miR-124-3p overexpression in rat myocardial fibroblasts resulted in reduced levels of AXIN1, whereas β-catenin, collagen 1, and α-SMA were elevated, suggesting that AXIN1 regulates activation and proliferation of myocardial fibroblasts through Wnt/β-catenin signaling.[40] Although Flt3L levels were related to NITs of liver fibrosis and decreases following different ablation techniques used to treat Afib,[38] its relationship with AFib was insignificant in the validation study. The relationship of the FIB-4 index as a continuous variable with DNER and CCL20 was of minor importance compared with the other proteins that were identified, whereas the relationship between the FIB-4 index as a clinically-relevant categorical (≥1.3/<1.3) variable and these proteins was insignificant. Therefore, CXCL10 is the most important liver-related factor in MASLD-related liver fibrosis

and can maintain a pathogenic environment for AFib, likely a result of atrial myocyte fibrosis and atherosclerosis.[36,37] However, the exact mechanism of how CXCL10 contributes to AFib in the setting of liver fibrosis remains to be determined. CXCL10 may be responsible for the recurrence of AFib after ablation, as liver fibrosis determined by the FIB-4 index has been shown to be an independent predictor for AFib recurrence after ablation in a 1-year follow-up study.[41]

Our study should be interpreted considering several limitations. First, the follow-up period for incident AFib was 5 years which might have been too short to define a predictive role of liver fibrosis in the development of AFib. Secondly, the inflammatory panel was limited to 92 targets using the Olink assay; thus, other markers related to liver fibrosis and AFib may have been missed. Thirdly, participants with chronic viral hepatitis or other causes of liver fibrosis different from MASLD were not specifically assessed through testing and thus occult infections could also have been missed. In contrast, MASLD is by far the most common cause of liver fibrosis in the Western population[42] and chronic viral hepatitis has a relatively low prevalence at the general population level in Germany (age-standardized prevalence rate [cirrhosis and other chronic liver diseases associated with to hepatitis] per 100,000: 548.22 for hepatitis C and 284.79 for hepatitis B).[43,44] In addition, the sample size of our study, the application of different parameters for assessing hepatic fibrosis, and reproduction of the results after a 5-year interval, allowed for accurate, real-world estimations.

In conclusion, CXCL10 was identified through targeted proteomics and can be considered a biomarker at the interface between the risk of advanced liver fibrosis and prevalent AFib in the general population. Targeting the drivers of hepatic and cardiac inflammation and fibrosis could allow for evidence-based drug development for patients with metabolic inflammation, MASLD, and AFib.

Affiliations

[1]*Metabolic Liver Research Center, Department of Medicine, University Medical Center Mainz, Mainz, Germany;* [2]*I. Department of Medicine, University Medical Center Mainz, Mainz, Germany;* [3]*In Vitro Liver Disease Modelling Team, Department of In Vitro Toxicology and Dermato-Cosmetology, Faculty of Medicine and Pharmacy, Vrije Universiteit Brussel, Brussels, Belgium;* [4]*Department of Medicine II, Saarland University Medical Center, Homburg, Germany;* [5]*Preventive Cardiology and Preventive Medicine, Department of Cardiology, University Medical Center of the Johannes Gutenberg-University Mainz, Mainz, Germany;* [6]*Clinic for Psychiatry and Psychotherapy, University Medical Center of the Johannes Gutenberg-University Mainz, Mainz, Germany;* [7]*Institute of Molecular Biology (IMB), Mainz, Germany;* [8]*Leibniz Institute for Resilience Research, Mainz, Germany;* [9]*Center for Thrombosis and Hemostasis, University Medical Center of the Johannes Gutenberg University Mainz, Germany;* [10]*Department of Cardiology, Democritus University of Thrace, Alexandroupolis, Greece;* [11]*Institute of Medical Biostatistics, Epidemiology and Informatics, University Medical Center of the Johannes Gutenberg-University Mainz, Mainz, Germany;* [12]*Department of Cardiology, Cardiology I, University Medical Center of the Johannes Gutenberg University, Mainz, Germany;* [13]*German Center for Cardiovascular Research (DZHK), partner site Rhine-Main, Mainz, Germany;* [14]*Institute of Clinical Chemistry and Laboratory Medicine, University Medical Center of the Johannes Gutenberg-University Mainz, Mainz, Germany;* [15]*Department of Psychosomatic Medicine and Psychotherapy, University Medical Center of the Johannes Gutenberg University Mainz, Mainz, Germany;* [16]*Department of Ophthalmology, University Medical Center of the Johannes Gutenberg University Mainz, Mainz, Germany*

Abbreviations

AFib, atrial fibrillation; APRI, AST to platelet index; AXIN1, axis inhibition protein 1; CCL20, C-C motif chemokine ligand 20; CDCP1, CUB domain containing protein 1; CXCL, C-X-C motif chemokine ligand; DNER, delta and notch-like epidermal growth factor-related receptor; FGF23, fibroblast growth factor 23; FIB-4, fibrosis-4; FLI, fatty liver index; Flt3L, Fms related receptor tyrosine kinase 3 ligand; HCC, hepatocellular carcinoma; HR, hazard ratio; IL, interleukin; MASLD, metabolic dysfunction-associated steatotic liver disease; NFS, NAFLD fibrosis score; NIT, non-invasive test; NRTN, neurturin; NT-proBNP, N-terminal pro–B-type natriuretic peptide; OR, odds ratio; SIRT2, sirtuin 2; STAMBP, STAM Binding Protein; VCTE, vibration-controlled transient elastography.

Financial support

The Gutenberg Health Study is funded by the government of Rhineland-Palatinate (“Stiftung Rheinland-Pfalz für Innovation”, contract AZ 961-386261/733), the research programs “Wissen schafft Zukunft” and “Center for Translational Vascular Biology (CTVB)” of the Johannes Gutenberg-University of Mainz, and its contract with Boehringer Ingelheim and PHILIPS Medical Systems, including an unrestricted grant for the Gutenberg Health Study. J.B. receives funding from Colgate-Palmolive – Society of Toxicology, Onderzoeksraad Vrije Universiteit Brussel, and Chair Mireille Aerens for the Development of Alternative Methods.

Conflicts of interest

A.K.S. received financial and research support by Abbvie, Apellis, Bayer Vital, Heidelberg Engineering, Novartis, Santen and has acted as consultant for Apellis. P.S.W. reports grants from Bayer AG, non-financial grants from Philips Medical Systems, grants and consulting fees from Boehringer Ingelheim, grants and consulting fees from Novartis Pharma, grants and consulting fees from Sanofi-Aventis, grants, consulting and lecturing fees from Bayer Health Care, grants from Daiichi Sankyo Europe, consulting fees from Astra Zeneca, consulting fees and non-financial support from Diasorin and non-financial support from I.E.M. Independent of this research study, J.M.S. has acted as Consultant to Apollo Endosurgery, Albireo Pharma Inc, Bayer, Boehringer Ingelheim, Gilead Sciences, GSK, Intercept Pharmaceuticals, Ipsen, Inventiva Pharma, Madrigal, MSD, Northsea Therapeutics, Novartis, Novo Nordisk, Pfizer, Roche, Sanofi, Siemens Healthineers; has received research Funding from Gilead Sciences, Boehringer Ingelheim, Siemens Healthcare GmbH and Speaker Honorarium from Boehringer Ingelheim, Echosens, MedPublico GmbH, Novo Nordisk, Madrigal Pharmaceuticals. All other authors report no relevant conflict of interest.

Please refer to the accompanying ICMJE disclosure forms for further details.

Authors’ contributions

Conceptualization: JB, MM, AG, TM, PSW, PRG, JMS. Data Curation: TM, PSW, PRG. Formal Analysis: JB, MM, AG, JMS. Funding Acquisition: TM, PSW, PRG. Investigation: JB, MM, AG, PSW, JMS. Methodology: JB, MM, AG, PSW, JMS. Project Administration: TM, PSW, PRG, JMS. Resources: TM, PSW, PRG. Software: J.B., TM, PSW, PRG. Supervision: PSW, JMS. Validation: AG. Visualization: JB, AG. Writing – Original Draft: JB, MM, JMS. Writing – Review & Editing: all authors.

Data availability statement

The data that support the findings of this study are available from the corresponding author upon reasonable request.

Acknowledgements

The authors thank all study participants of the Gutenberg Health study for their willingness to participate and all the GHS study team (http://www.gutenberg-gesundheitsstudie.de/ghs/team/studienzentrum.html) for their support. Servier Medical Art was used to make the graphical abstract.

Supplementary data

Supplementary data to this article can be found online at https://doi.org/10.1016/j.jhepr.2024.101171.

References

Author names in bold designate shared co-first authorship

[1] Anstee QM, Mantovani A, Tilg H, et al. Risk of cardiomyopathy and cardiac arrhythmias in patients with nonalcoholic fatty liver disease. Nat Rev Gastroenterol Hepatol 2018;15:425–439.

[2] Boeckmans J, Sandrin L, Knackstedt C, et al. Liver stiffness as a cornerstone in heart disease risk assessment. Liver Int 2024;44:344–356.
[3] Brundel BJJM, Ai X, Hills MT, et al. Atrial fibrillation. Nat Rev Dis Primers 2022;8:21.
[4] Elliott AD, Middeldorp ME, Van Gelder IC, et al. Epidemiology and modifiable risk factors for atrial fibrillation. Nat Rev Cardiol 2023;6:404–417.
[5] **Wai-Sun Wong V**, **Ekstedt M**, Lai-Hung Wong G, et al. Changing epidemiology, global trends and implications for outcomes of NAFLD. J Hepatol 2023;79:842–852.
[6] Kotecha D, Piccini JP. Atrial fibrillation in heart failure: what should we do? Eur Heart J 2015;36:3250–3257.
[7] Odutayo A, Wong CX, Hsiao AJ, et al. Atrial fibrillation and risks of cardiovascular disease, renal disease, and death: systematic review and meta-analysis. BMJ 2016;354:i4482.
[8] Lacoste JL, Szymanski TW, Avalon JC, et al. Atrial fibrillation management: a comprehensive review with a focus on pharmacotherapy, rate, and rhythm control strategies. Am J Cardiovasc Drugs 2022;22:475–496.
[9] Van Wagoner DR, Chung MK. Inflammation, inflammasome activation, and atrial fibrillation: evidence for causation and new therapeutic targets. Circulation 2018;138:2243–2246.
[10] van Kleef LA, Lu Z, Arfan Ikram M, et al. Liver stiffness not fatty liver disease is associated with atrial fibrillation: the Rotterdam study. J Hepatol 2022;77:931–938.
[11] Gehrke N, Schattenberg JM. Metabolic inflammation—a role for hepatic inflammatory pathways as drivers of comorbidities in nonalcoholic fatty liver disease? Gastroenterology 2020;158:1929–1947.e6.
[12] Yang L, Hao Y, Boeckmans J, et al. Immune cells and their derived microRNA-enriched extracellular vesicles in nonalcoholic fatty liver diseases: novel therapeutic targets. Pharmacol Ther 2023;243:108353.
[13] Haghbin H, Gangwani MK, Ravi SJK, et al. Nonalcoholic fatty liver disease and atrial fibrillation: possible pathophysiological links and therapeutic interventions. Ann Gastroenterol 2020;33(6):603–614.
[14] Ding YH, Ma Y, Qian LY, et al. Linking atrial fibrillation with non-alcoholic fatty liver disease: potential common therapeutic targets. Oncotarget 2017;8:60673–60683.
[15] Serra-Burriel M, Juanola A, Serra-Burriel F, et al. Development, validation, and prognostic evaluation of a risk score for long-term liver-related outcomes in the general population: a multicohort study. Lancet 2023;402:988–996.
[16] Wild PS, Zeller T, Beutel M, et al. Die Gutenberg gesundheitsstudie. Bundesgesundheitsblatt Gesundheitsforschung Gesundheitsschutz 2012;55:824–830.
[17] Ratziu V, Bellantani S, Cortez-Pinto H, et al. A position statement on NAFLD/NASH based on the EASL 2009 special conference. J Hepatol 2010;53:372–384.
[18] Sterling RK, Lissen E, Clumeck N, et al. Development of a simple noninvasive index to predict significant fibrosis in patients with HIV/HCV coinfection. Hepatology 2006;43:1317–1325.
[19] Bedogni G, Bellentani S, Miglioli L, et al. The Fatty Liver Index: a simple and accurate predictor of hepatic steatosis in the general population. BMC Gastroenterol 2006;6:1–7.
[20] Angulo P, Hui JM, Marchesini G, et al. The NAFLD fibrosis score: a noninvasive system that identifies liver fibrosis in patients with NAFLD. Hepatology 2007;45:846–854.
[21] Wai CT, Greenson JK, Fontana RJ, et al. A simple noninvasive index can predict both significant fibrosis and cirrhosis in patients with chronic hepatitis C. Hepatology 2003;38:518–526.
[22] Huber Y, Schulz A, Schmidtmann I, et al. Prevalence and risk factors of advanced liver fibrosis in a population-based study in Germany. Hepatol Commun 2022;6:1457–1466.
[23] Werhahn SM, Becker C, Mende M, et al. NT-proBNP as a marker for atrial fibrillation and heart failure in four observational outpatient trials. ESC Heart Fail 2022;9:100–109.
[24] McPherson S, Hardy T, Dufour JF, et al. Age as a confounding factor for the accurate non-invasive diagnosis of advanced NAFLD fibrosis. Am J Gastroenterol 2017;112:740–751.
[25] Park HE, Lee H, Choi SY, et al. The risk of atrial fibrillation in patients with non-alcoholic fatty liver disease and a high hepatic fibrosis index. Sci Rep 2020;10:5023.
[26] Tanaka Y, Chuzi S, Morikawa N, et al. Association between the Liver Fibrosis-4 Score and incident atrial fibrillation in a general Japanese population of middle-aged to older adults. Circulation 2021;143:AP004.
[27] Boos CJ, Anderson RA, Lip GYH. Is atrial fibrillation an inflammatory disorder? Eur Heart J 2006;27:136–149.
[28] Nso N, Bookani KR, Metzl M, et al. Role of inflammation in atrial fibrillation: a comprehensive review of current knowledge. J Arrhythm 2021;37:1–10.
[29] Harrison SA, Allen AM, Dubourg J, et al. Challenges and opportunities in NASH drug development. Nat Med 2023;29:562–573.
[30] Xu Z, Zhang X, Lau J, Yu J. C-X-C motif chemokine 10 in non-alcoholic steatohepatitis: role as a pro-inflammatory factor and clinical implication. Expert Rev Mol Med 2016;18:e16.
[31] Martins GL, Duarte RCF, Vieira ÉLM, et al. Evaluation of new potential inflammatory markers in patients with nonvalvular atrial fibrillation. Int J Mol Sci 2023;24:3326.
[32] Heller EA, Liu E, Tager AM, et al. Chemokine CXCL10 promotes atherogenesis by modulating the local balance of effector and regulatory T cells. Circulation 2006;113:2301–2312.
[33] Altara R, Manca M, Hessel MH, et al. CXCL10 Is a circulating inflammatory marker in patients with advanced heart failure: a Pilot Study. J Cardiovasc Transl Res 2016;9:302–314.
[34] Ibrahim SH, Hirsova P, Tomita K, et al. Mixed lineage kinase 3 mediates release of C-X-C motif ligand 10-bearing chemotactic extracellular vesicles from lipotoxic hepatocytes. Hepatology 2015;63:731–744.
[35] Zhang X, Shen J, Man K, et al. CXCL10 plays a key role as an inflammatory mediator and a non-invasive biomarker of non-alcoholic steatohepatitis. J Hepatol 2014;61:1365–1375.
[36] Chen Y, Chen X, Li H, et al. Serum extracellular vesicles containing MIAT induces atrial fibrosis, inflammation and oxidative stress to promote atrial remodeling and atrial fibrillation via blockade of miR-485-5p-mediated CXCL10 inhibition. Clin Transl Med 2021;11:e482.
[37] Heeringa J, Van Der Kuip DAM, Hofman A, et al. Subclinical atherosclerosis and risk of atrial fibrillation. Arch Intern Med 2007;167:382–387.
[38] **Lin M**, **Bao Y**, **Du Z**, et al. Plasma protein profiling analysis in patients with atrial fibrillation before and after three different ablation techniques. Front Cardiovasc Med 2023;9:1077992.
[39] **Kriss M**, **Golden-Mason L**, Kaplan J, et al. Increased hepatic and circulating chemokine and osteopontin expression occurs early in human NAFLD development. PLoS One 2020;15:e0236353.
[40] Zhu P, Li H, Zhang A, et al. MicroRNAs sequencing of plasma exosomes derived from patients with atrial fibrillation: miR-124-3p promotes cardiac fibroblast activation and proliferation by regulating AXIN1. J Physiol Biochem 2022;78:85–98.
[41] Wang Z, Wang Y, Luo F, et al. Impact of advanced liver fibrosis on atrial fibrillation recurrence after ablation in non-alcoholic fatty liver disease patients. Front Cardiovasc Med 2022;9:960259.
[42] Caballería L, Pera G, Arteaga I, et al. High prevalence of liver fibrosis among European adults with unknown liver disease: a population-based study. Clin Gastroenterol Hepatol 2018;16:1138–1145.e5.
[43] **Steffen G**, **Sperle I**, Leendertz SA, et al. The epidemiology of Hepatitis B, C and D in Germany: a scoping review. PLoS One 2020;15(3):e0229166.
[44] **Cortesi PA**, **Fornari C**, **Conti S**, et al. Hepatitis B and C in Europe: an update from the global burden of disease study 2019. Lancet Public Health 2023;8:e701–e716.

Keywords: Atrial fibrillation; C-X-C motif chemokine ligand 10 (CXCL10); Fibrosis-4 index (FIB-4 index); Metabolic dysfunction-associated steatotic liver disease (MASLD); Non-invasive test; Proteomics.
Received 5 January 2024; received in revised form 14 June 2024; accepted 15 July 2024; Available online 18 July 2024

Journal of Hepatology, Volume *6*

Supplemental information

Inflammation in liver fibrosis and atrial fibrillation: A prospective population-based proteomic study

Joost Boeckmans, Maurice Michel, Alexander Gieswinkel, Oliver Tüscher, Stavros V. Konstantinides, Jochem König, Thomas Münzel, Karl J. Lackner, Jasmin Ghaemi Kerahrodi, Alexander K. Schuster, Philipp S. Wild, Peter R. Galle, and Jörn M. Schattenberg

Supplementary material

Inflammation in liver fibrosis and atrial fibrillation: a prospective population-based proteomic study

Joost Boeckmans, Maurice Michel, Alexander Gieswinkel, Oliver Tüscher, Stavros V. Konstantinides, Jochem König, Thomas Münzel, Karl J. Lackner, Jasmin Ghaemi Kerahrodi, Alexander K. Schuster, Philipp S. Wild, Peter R. Galle, Jörn M. Schattenberg.

Table of contents:

1. Abbreviations…………………………………………… p. 2
2. Definitions of diseases and parameters…………………p. 3
3. Supplementary figures……………………………………p. 5
4. Supplementary tables……………………………………..p. 14

1. Abbreviations

AFib: atrial fibrillation

APRI: AST to platelet index

AUC: area under the curve

AXIN1: Axis inhibition protein 1

BMI: body mass index

CCL20: C-C motif chemokine ligand 20

CI: confidence interval

CXCL10: C-X-C motif chemokine ligand 10

DNER: Delta and Notch-like epidermal growth factor-related receptor

eGFR: estimated glomerular filtration rate

FIB-4: fibrosis-4

Flt3L: Fms related receptor tyrosine kinase 3 ligand

HDL: high-density lipoprotein

L: lower

LDL: low-density lipoprotein

NFS: NAFLD fibrosis score

SD: standard deviation

U: upper

y: year

2. Definitions of diseases and parameters

(self-reporting through questionnaires)

Smoking

- Self-report for "active smoking"

Arterial hypertension

- Self-report or
- Systolic blood pressure > 140 mm Hg or
- Diastolic blood pressure > 90 mmHg

Diabetes mellitus

- Self-report or
- Intake of A10 medication or
- HbA1c ≥ 6.5 %

Obesity

- BMI ≥ 30 kg/m^2

Dyslipidemia

- LDL/HDL > 3.5 or
- Intake of C10 medication

Coronary artery disease

- Self-report

Metabolic syndrome

- If you have 3 or more of the following characteristics:
    - Waist ≥ 94 cm (men) / ≥ 80 cm (women)
    - HDL ≤ 40 mg/dL (men) / ≤ 45 mg/dL (women)
    - Glucose > 100 mg/dL (≥ 8h fasting)
    - Triglycerides > 150 mg/dL (≥ 8h fasting)
    - Arterial hypertension

Hyperuricemia

- Uric acid > 7 mg/dL

Atrial fibrillation

- Self-report

Myocardial infarction

- Self-report

Peripheral artery disease

- Self-report

Congestive heart failure

- Self-report

Chronic kidney disease

- Self-report

eGFR

- Calculated via the CKD-EPI-Formula

Family history of myocardial infarction/stroke

- Self-report

3. <u>Supplementary figures</u>

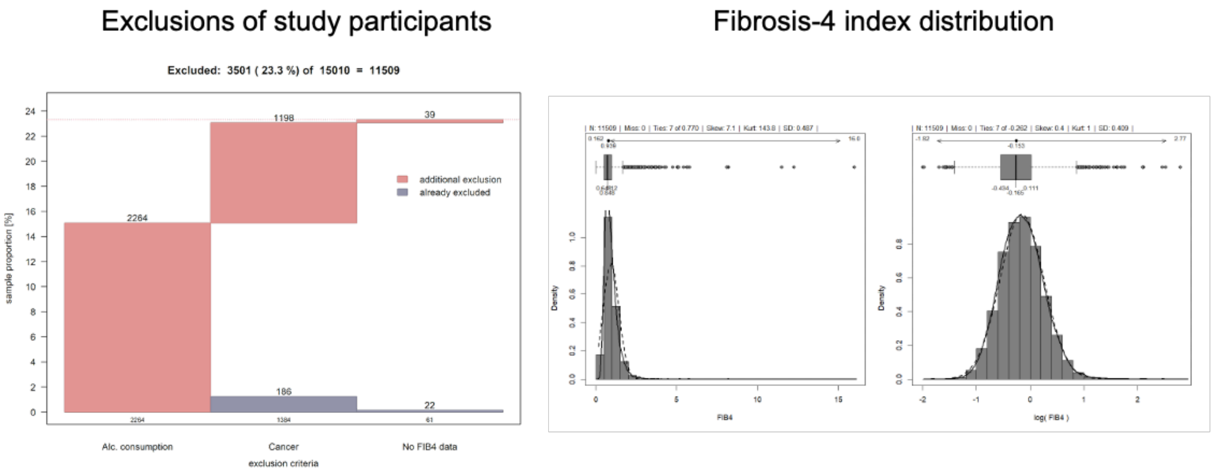

Fig. S1. Exclusions of study participants (left) and distribution of the fibrosis-4 index in de study sample (right). [Abbreviations: FIB-4, fibrosis-4]

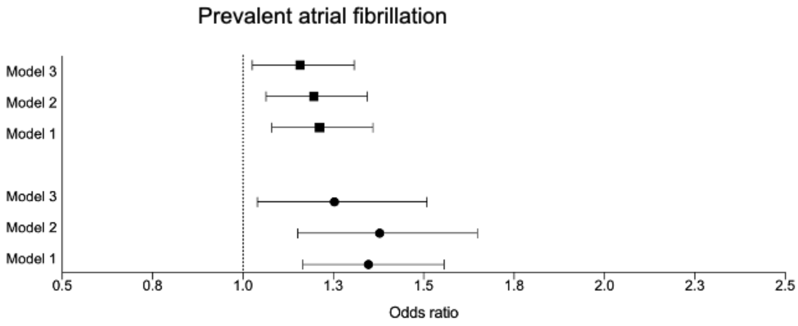

OR	L 95%CI	U 95%CI	p-value
1.158	1.026	1.308	**0.018**
1.196	1.064	1.344	**0.0026**
1.212	1.080	1.359	**0.0010**
1.253	1.040	1.509	**0.017**
1.378	1.152	1.648	**0.00046**
1.347	1.166	1.556	**<0.0001**

■ Log(APRI) per SD
● NFS per SD

Fig. S2. Relationship between the NFS and APRI, and prevalent atrial fibrillation. Relationship between NFS and APRI, and atrial fibrillation (symbols represent odds ratios and bars represent 95% CIs). Levels of significance: exact p-values on the figure, $p < 0.05$ is considered as statistically significant, significant values in bold (multivariate logistic regression, z-test; NFS model 1: N = 11373 (274 events); model 2: N = 11335 (274 events); model 3: N = 11186 (262 events) ; APRI model 1: N = 11395 (275 events); model 2: N = 11335 (274 events); model 3: N = 11186 (262 events)). [model 1: adjusted for age and sex; model 2: additional adjustment for smoking, arterial hypertension, diabetes mellitus, obesity, and dyslipidemia; model 3: additional adjustment for coronary artery disease and congestive heart failure] [Abbreviations: APRI, AST to platelet index; NFS, NAFLD fibrosis score; OR, odds ratio, CI, confidence inteval; L, lower; SD, standard deviation; U, upper]

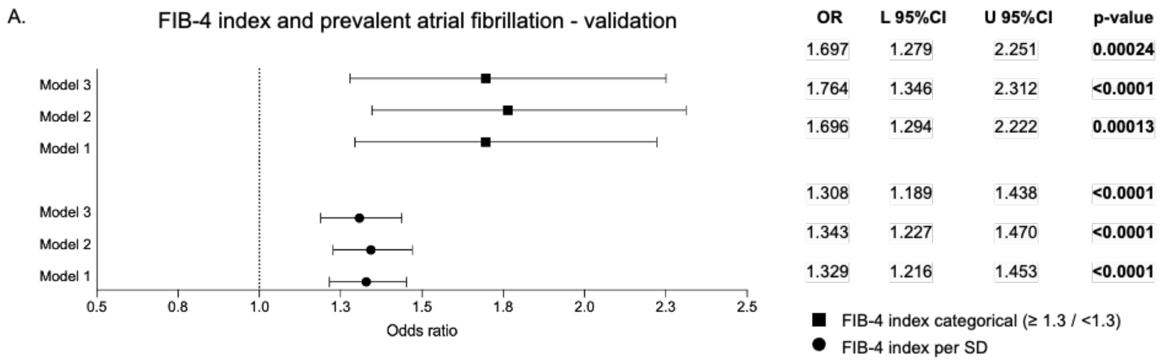

OR	L 95%CI	U 95%CI	p-value
1.697	1.279	2.251	**0.00024**
1.764	1.346	2.312	**<0.0001**
1.696	1.294	2.222	**0.00013**
1.308	1.189	1.438	**<0.0001**
1.343	1.227	1.470	**<0.0001**
1.329	1.216	1.453	**<0.0001**

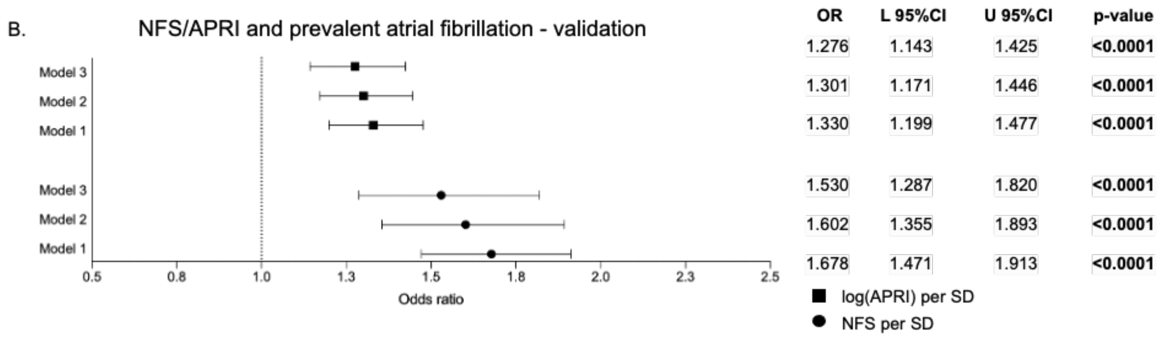

OR	L 95%CI	U 95%CI	p-value
1.276	1.143	1.425	**<0.0001**
1.301	1.171	1.446	**<0.0001**
1.330	1.199	1.477	**<0.0001**
1.530	1.287	1.820	**<0.0001**
1.602	1.355	1.893	**<0.0001**
1.678	1.471	1.913	**<0.0001**

Fig. S3. Validation of the relationship between non-invasive tests for hepatic fibrosis and atrial fibrillation. (A) Relationship between the FIB-4 index and prevalent atrial fibrillation at 5 years follow-up (symbols represent odds ratios and bars represent 95% CIs). Levels of significance: exact p-values on the figure, $p < 0.05$ is considered as statistically significant, significant values in bold (multivariate logistic regression, z-test; model 1: N = 9290 (325 events); model 2: N = 9265 (325 events); model 3: N = 9192 (312 events)). (B) Relationship between the NFS and APRI and prevalent atrial fibrillation at 5 years follow-up (symbols represent odds ratios and bars represent 95% CIs). Levels of significance: exact p-values on the figure, $p < 0.05$ is considered as statistically significant, significant values in bold (multivariate logistic regression, z-test; NFS model 1: N = 9281 (325 events); model 2: N = 9265 (325 events); model 3: N = 9192 (312 events); APRI model 1: N = 9290 (325 events); model 2: N = 9265 (325 events); model 3: N = 9192 (312 events)). [model 1: adjusted for age and sex; model 2: additional adjustment for smoking, arterial hypertension, diabetes mellitus, obesity, and dyslipidemia; model 3: additional adjustment for coronary artery disease and congestive heart failure] [Abbreviations: APRI, AST to platelet index; CI, confidence inteval; FIB-4, fibrosis-4; L, lower; NFS, NAFLD fibrosis score; SD, standard deviation; U, upper]

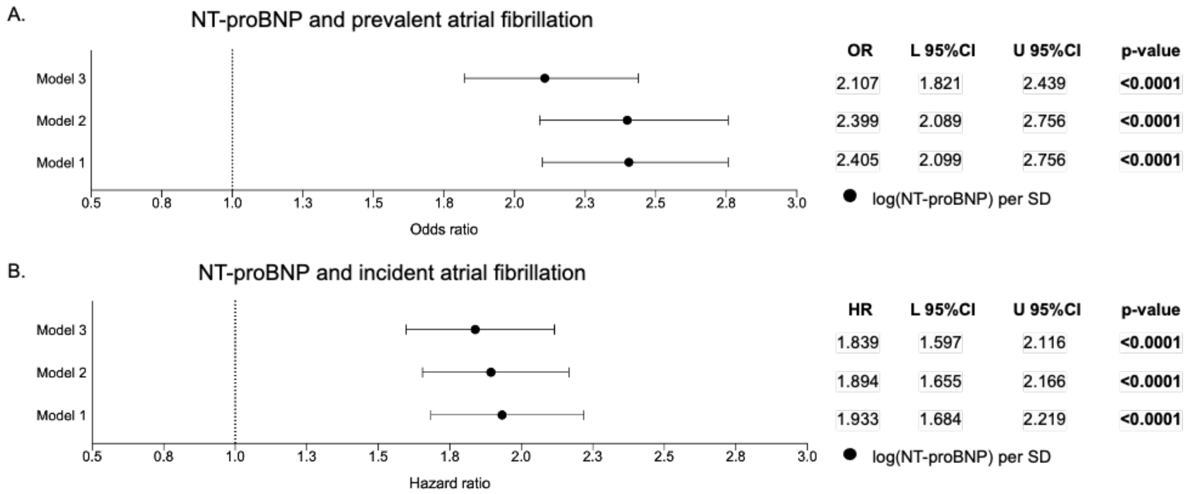

Fig. S4. NT-proBNP in relation to prevalent and incident atrial fibrillation. (A) Relationship between log(NT-proBNP) with prevalent atrial fibrillation (symbols represent odds ratios and bars represent 95% CIs). Levels of significance: exact p-values on the figure, $p < 0.05$ is considered as statistically significant, significant values in bold (multivariate logistic regression, z-test; model 1: N = 11006 (267 events); model 2: N = 10947 (266 events); model 3: N = 10804 (254 events)). (B) Relationship between log(NT-proBNP) and incident atrial fibrillation (symbols represent hazard ratios and bars represent 95% CIs). Levels of significance: exact p-values on the figure, $p < 0.05$ is considered as statistically significant, significant values in bold (Cox competing risk analysis, event = atrial fibrillation, competing event = death, z-test; model 1: N = 10427 (237 events); model 2: N = 10369 (237 events); model 3: N = 10230 (224 events)). [model 1: adjusted for age and sex; model 2: additional adjustment for smoking, arterial hypertension, diabetes mellitus, obesity, and dyslipidemia; model 3: additional adjustment for coronary artery disease and congestive heart failure] [Abbreviations: CI, confidence inteval; HR, hazard ratio; L, lower; NT-proBNP, N-terminal pro–B-type natriuretic peptide; OR, odds ratio; U, upper]

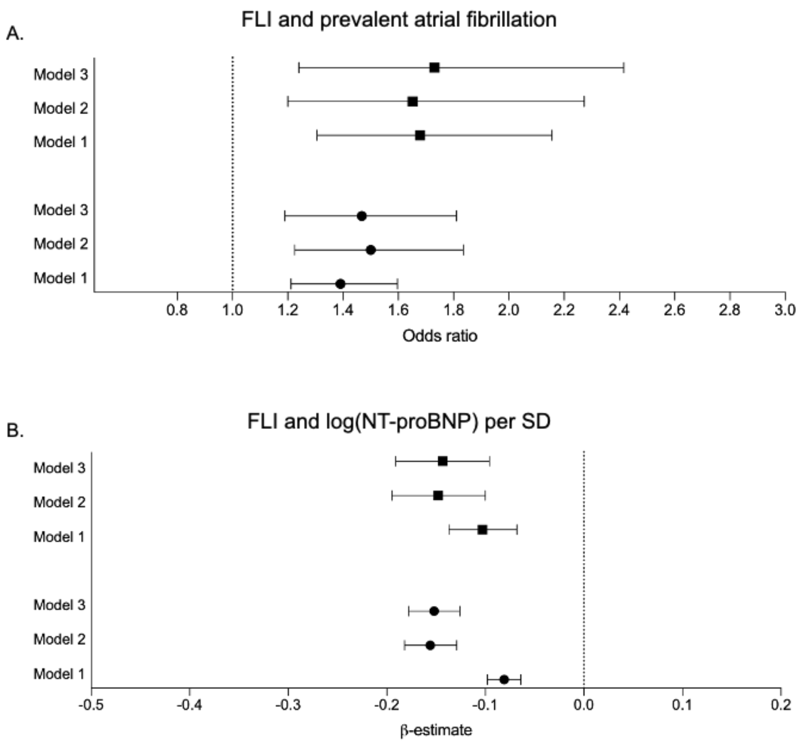

OR	L 95%CI	U 95%CI	p-value
1.731	1.240	2.416	**0.0013**
1.652	1.200	2.273	**0.0021**
1.679	1.306	2.156	**<0.0001**
1.468	1.190	1.811	**0.00034**
1.500	1.225	1.835	**<0.0001**
1.391	1.212	1.597	**<0.0001**

■ FLI categorical (≥ 60 / < 60)
● FLI per SD

β	L 95%CI	U 95%CI	p-value
-0.143	-0.191	-0.096	**<0.0001**
-0.148	-0.195	-0.100	**<0.0001**
-0.103	-0.137	-0.068	**<0.0001**
-0.152	-0.178	-0.126	**<0.0001**
-0.156	-0.182	-0.129	**<0.0001**
-0.081	-0.098	-0.064	**<0.0001**

■ FLI categorical (≥ 60 / < 60)
● FLI per SD

Fig. S5. Relation between the fatty liver index and atrial fibrillation. (A) Relationship between the FLI and prevalent atrial fibrillation (symbols represent odds ratios and bars represent 95% CIs). Levels of significance: exact p-values on the figure, $p < 0.05$ is considered as statistically significant, significant values in bold (multivariate logistic regression, z-test; model 1: N = 11385 (274 events); model 2: N = 11332 (274 events); model 3: N = 11183 (262 events)). (B) Relationship between the FLI and NT-proBNP (symbols represent β-estimates and bars represent 95% CIs) . Levels of significance: exact p-values on the figure, $p < 0.05$ is considered as statistically significant, significant values in bold (multivariate linear regression, t-test; model 1: N = 11105; model 2: N = 11051; model 3: N = 10888). [model 1: adjusted for age and sex; model 2: additional adjustment for smoking, arterial hypertension, diabetes mellitus, obesity, and dyslipidemia; model 3: additional adjustment for coronary artery disease and congestive heart failure] [Abbreviations: FLI, fatty liver index; CI, confidence inteval; L, lower; NT-proBNP, N-terminal pro–B-type natriuretic peptide; U, upper; SD, standard deviation]

	FIB-4 ≥ 1.3	FIB-4 <1.3	AFib	No AFib
P02778 (CXCL10)	44.1	-9.2	58.5	-2.1
Q9H5V8 (CDCP1)	63.6	-13.3	56.2	-2.3
Q9GZV9 (FGF 23)	12.6	-2.6	54.0	-2.2
P78556 (CCL20)	15.8	-3.3	53.1	-2.1
Q07325 (CXCL9)	49.3	-10.3	47.4	-1.9
O15444 (CCL25)	34.3	-7.2	46.9	-2.0
P10147 (CCL3)	35.4	-7.4	44.7	-1.6
P05231 (IL6)	21.3	-4.4	44.3	-1.8
P14210 (HGF)	34.7	-7.2	42.5	-1.7
Q07011 (TNFRSF9)	32.2	-6.7	41.2	-1.5
P80511 (EN RAGE)	11.7	-2.4	40.7	-1.7
P10145 (IL8)	28.9	-6.0	39.6	-1.6
P15692 (VEGFA)	7.7	-1.6	37.4	-1.5
Q13261 (IL 15RA)	29.0	-6.0	36.9	-1.3
P49771 (Flt3L)	38.0	-7.9	36.8	-1.4
Q9NSA1 (FGF 21)	17.2	-3.6	36.5	-1.8
P80098 (MCP 3)	26.6	-5.6	35.4	-1.3
Q14790 (CASP 8)	9.9	-2.1	35.0	-1.3
Q13541 (X4E BP1)	-8.5	1.8	34.4	-1.3
P29460 (IL 12B)	22.5	-4.7	28.9	-1.1
Q9P0M4 (IL 17C)	12.4	-2.6	28.9	-1.1
Q14116 (IL18)	16.5	-3.5	28.4	-1.2
P01135 (TGF alpha)	14.8	-3.1	27.5	-1.0
Q13478 (IL 18R1)	18.1	-3.8	27.2	-1.3
Q99731 (CCL19)	8.1	-1.7	26.9	-1.0
P51671 (CCL11)	24.7	-5.2	26.7	-0.8
P42702 (LIF R)	31.2	-6.5	26.3	-1.2
P13236 (CCL4)	22.0	-4.6	26.0	-0.8
O00300 (OPG)	34.5	-7.2	25.3	-1.2
P28325 (CST5)	30.2	-6.3	24.7	-0.8
Q9NZQ7 (PD L1)	15.2	-3.2	23.5	-0.9
P01375 (TNF)	11.8	-2.5	23.4	-0.6
P01579 (IFN gamma)	9.4	-2.0	20.3	-0.4
Q99616 (MCP 4)	12.8	-2.7	19.7	-0.6
Q08334 (IL 10RB)	17.6	-3.7	19.6	-0.9
P03956 (MMP 1)	-9.3	1.9	19.1	-0.7
P78423 (CX3CL1)	36.1	-7.5	18.9	-0.8
Q13291 (SLAMF1)	20.5	-4.3	18.2	-0.6
P30203 (CD6)	13.2	-2.8	17.8	-0.6
P13500 (MCP 1)	27.1	-5.6	16.9	-0.7
P25942 (CD40)	-2.1	0.4	15.8	-0.7
P01137 (LAP TGF beta 1)	-5.1	1.1	15.7	-0.5
P22301 (IL10)	12.8	-2.7	14.8	-0.3
P09603 (CSF 1)	11.9	-2.5	14.4	-0.6
P80075 (MCP 2)	12.5	-2.6	12.4	-0.5
O95750 (FGF 19)	11.7	-2.5	10.4	-0.5
Q16552 (IL 17A)	3.9	-0.8	10.4	-0.4
P09238 (MMP 10)	4.1	-0.9	10.2	-0.5
O14625 (CXCL11)	4.8	-1.0	9.4	-0.3
P12034 (FGF 5)	10.8	-2.2	8.3	-0.1
P00813 (ADA)	10.1	-2.1	8.3	0.0
Q9BZW8 (CD244)	-4.4	0.9	7.5	-0.3
P55773 (CCL23)	-0.5	0.1	6.7	-0.4
O14788 (TRANCE)	-18.2	3.8	6.7	0.1
P50225 (ST1A1)	-31.7	6.6	6.7	-0.1
P39905 (GDNF)	17.9	-3.7	5.4	-0.1
P01732 (CD8A)	6.6	-1.4	5.1	-0.1
P06127 (CD5)	0.8	-0.2	4.4	-0.2
P13725 (OSM)	-14.2	3.0	3.1	0.0
P80162 (CXCL6)	-10.2	2.1	2.9	0.0
Q9NRJ3 (CCL28)	18.2	-3.8	1.4	0.0
P00749 (uPA)	20.9	-4.4	0.8	0.1
P15018 (LIF)	-5.2	1.1	0.2	0.3
O43557 (TNFSF14)	-16.6	3.5	-1.7	0.1
P13232 (IL7)	-28.9	6.0	-2.1	0.1
P01374 (TNFB)	-3.5	0.7	-2.5	0.3
O95630 (STAMBP)	-36.3	7.6	-4.0	0.1
Q8N6P7 (IL 22 RA1)	-12.2	2.5	-5.0	0.2
P09341 (CXCL1)	-29.5	6.2	-5.7	0.2
P20783 (NT 3)	-14.4	3.0	-6.1	0.4
Q13651 (IL 10RA)	-8.1	1.7	-7.1	0.3
P05112 (IL4)	-7.7	1.6	-8.6	0.3
Q13007 (IL 24)	-7.0	1.5	-9.3	0.3
Q8IXJ6 (SIRT2)	-43.0	9.0	-10.1	0.4
P05113 (IL5)	-12.9	2.7	-10.5	0.6
P50591 (TRAIL)	-15.6	3.3	-11.1	0.7
P01138 (Beta NGF)	2.4	-0.5	-12.1	0.4
P42830 (CXCL5)	-27.1	5.7	-12.2	0.3
Q5T4W7 (ARTN)	-9.8	2.0	-12.4	0.6
Q969D9 (TSLP)	-6.3	1.3	-17.4	0.8
P21583 (SCF)	-0.8	0.2	-18.7	0.7
O95760 (IL33)	-7.9	1.6	-19.8	0.9
P01583 (IL 1 alpha)	-16.5	3.4	-20.6	0.8
P35225 (IL13)	-10.1	2.1	-21.1	1.0
P14784 (IL 2RB)	-8.0	1.7	-22.0	1.0
Q9UHF4 (IL 20RA)	-6.4	1.3	-23.2	0.8
Q9NYY1 (IL 20)	-11.9	2.5	-24.6	1.0
O15169 (AXIN1)	-43.2	9.0	-25.5	0.8
O43508 (TWEAK)	-8.2	1.7	-32.5	1.4
Q99748 (NRTN)	-5.6	1.2	-33.4	1.2
P60568 (IL2)	-18.8	3.9	-37.4	1.6
Q8NFT8 (DNER)	-16.3	3.4	-51.0	2.3

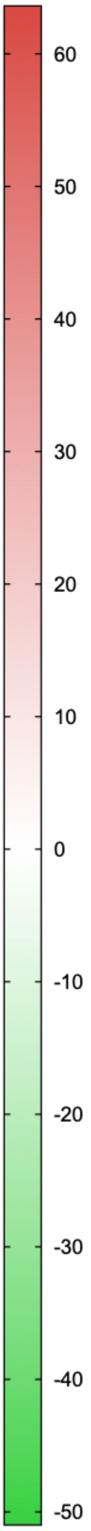

Protein expression
%SD of the overall mean

Fig. S6. Protein expressions in %SD of the overall mean in study participants with a fibrosis-4 index ≥ 1.3 and atrial fibrillation (sorted by atrial fibrillation). (color scale indicates protein expression rate; green = lower expression, and red = higher expression, compared to the overall mean) [Abbreviations: AFib, atrial fibrillation; FIB-4, fibrosis-4; SD, standard deviation]

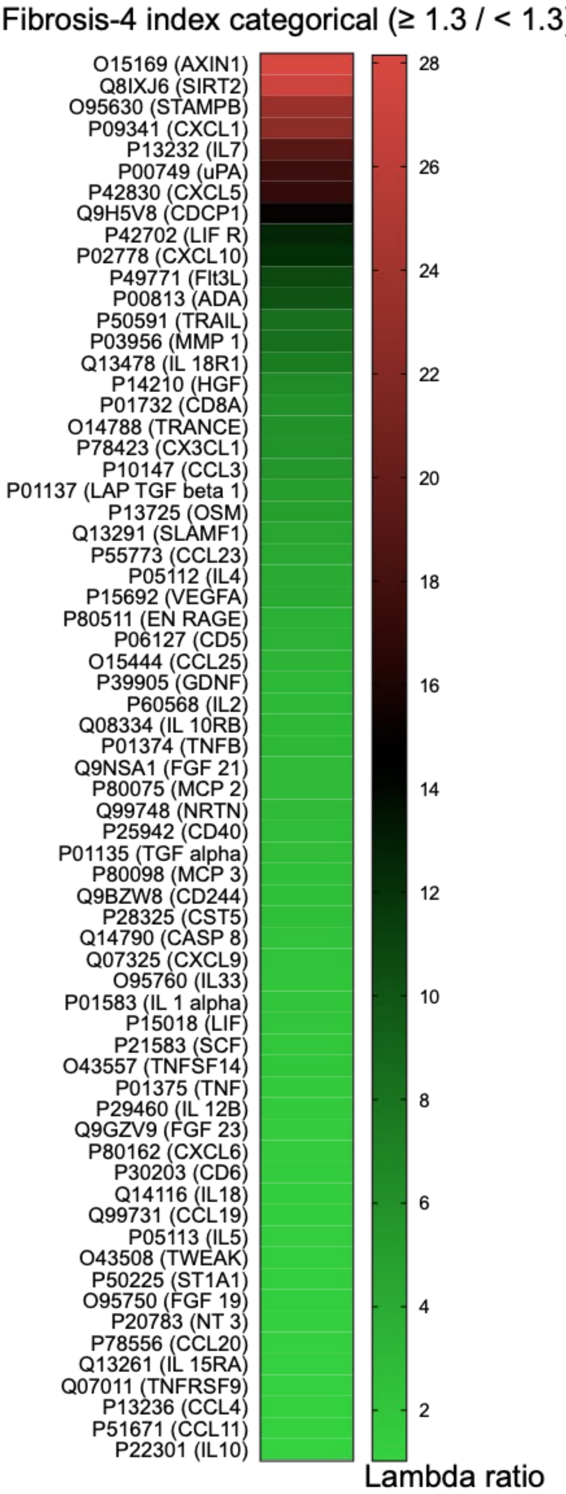

Fig. S7. Selection of the most important proteins related to liver fibrosis using the fibrosis-4 index as a categorical variable. Elastic net regularized regression for circulating proteins in liver fibrosis based on fibrosis-4 index categories (≥ 1.3 / < 1.3) (10 fold cross-validation AUC = 0.8870, (simple AUC = 0.8981), minimal lambda = 0.004 (10 fold-cross validation), N = 5741, events = 991, number of proteins = 92, adjusted for age [SD] and sex). Color scale indicates the lambda ratios within the spectrum of obtained results, green = lower, and red = higher lambda ratio [Abbreviations: AUC, area under the curve; SD, standard deviation]

A.

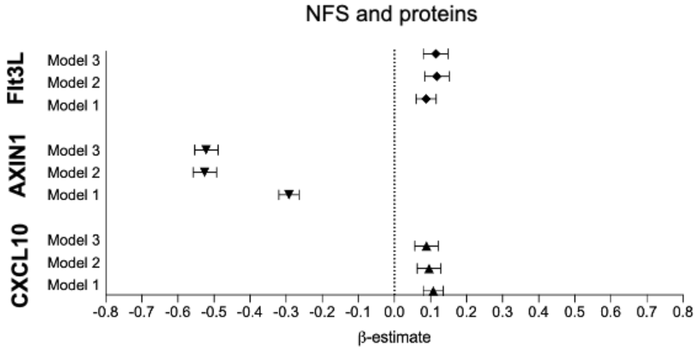

	β	L 95%CI	U 95%CI	p-value
Flt3L Model 3	0.115	0.080	0.149	**<0.0001**
Flt3L Model 2	0.118	0.084	0.152	**<0.0001**
Flt3L Model 1	0.088	0.060	0.116	**<0.0001**
AXIN1 Model 3	-0.522	-0.554	-0.489	**<0.0001**
AXIN1 Model 2	-0.526	-0.558	-0.493	**<0.0001**
AXIN1 Model 1	-0.292	-0.320	-0.264	**<0.0001**
CXCL10 Model 3	0.089	0.056	0.122	**<0.0001**
CXCL10 Model 2	0.096	0.063	0.129	**<0.0001**
CXCL10 Model 1	0.108	0.080	0.135	**<0.0001**

B.

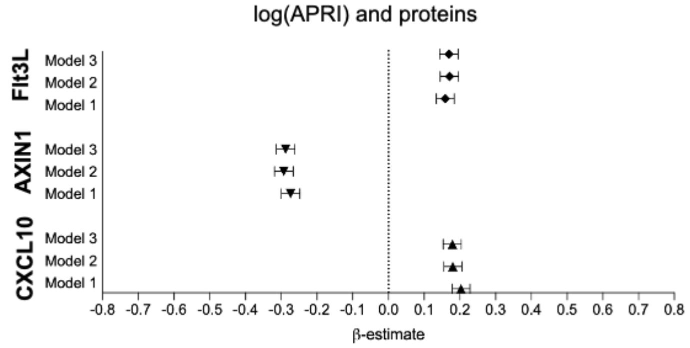

	β	L 95%CI	U 95%CI	p-value
Flt3L Model 3	0.169	0.143	0.195	**<0.0001**
Flt3L Model 2	0.171	0.145	0.196	**<0.0001**
Flt3L Model 1	0.159	0.134	0.184	**<0.0001**
AXIN1 Model 3	-0.288	-0.314	-0.262	**<0.0001**
AXIN1 Model 2	-0.293	-0.319	-0.267	**<0.0001**
AXIN1 Model 1	-0.274	-0.300	-0.248	**<0.0001**
CXCL10 Model 3	0.179	0.153	0.204	**<0.0001**
CXCL10 Model 2	0.180	0.155	0.205	**<0.0001**
CXCL10 Model 1	0.203	0.178	0.228	**<0.0001**

Fig. S8. Relationships between CXCL10, AXIN1, and Flt3L with the NFS and APRI. (A) Relationship between the NFS (per SD) and circulating proteins (per SD) (symbols represent β-estimates and bars represent 95% CIs). Levels of significance: exact p-values on the figure, $p < 0.05$ is considered as statistically significant, significant values in bold (multivariate linear regression, t-test; model 1: N = 5723; model 2: N = 5704; model 3: N = 5601). (B) Relationship between log(APRI) (per SD) and circulating proteins (per SD) (symbols represent β-estimates and bars represent 95% CIs). Levels of significance: exact p-values on the figure, $p < 0.05$ is considered as statistically significant, significant values in bold (multivariate linear regression, t-test; model 1: N = 5741; model 2: N = 5704; model 3: N = 5601). [model 1: adjusted for age and sex; model 2: additional adjustment for smoking, arterial hypertension, diabetes mellitus, obesity, and dyslipidemia; model 3: additional adjustment for coronary artery disease and congestive heart failure] [Abbreviations: APRI, AST to platelet index; CI, confidence inteval; L, lower; NFS, NAFLD fibrosis score; U, upper; SD, standard deviation]

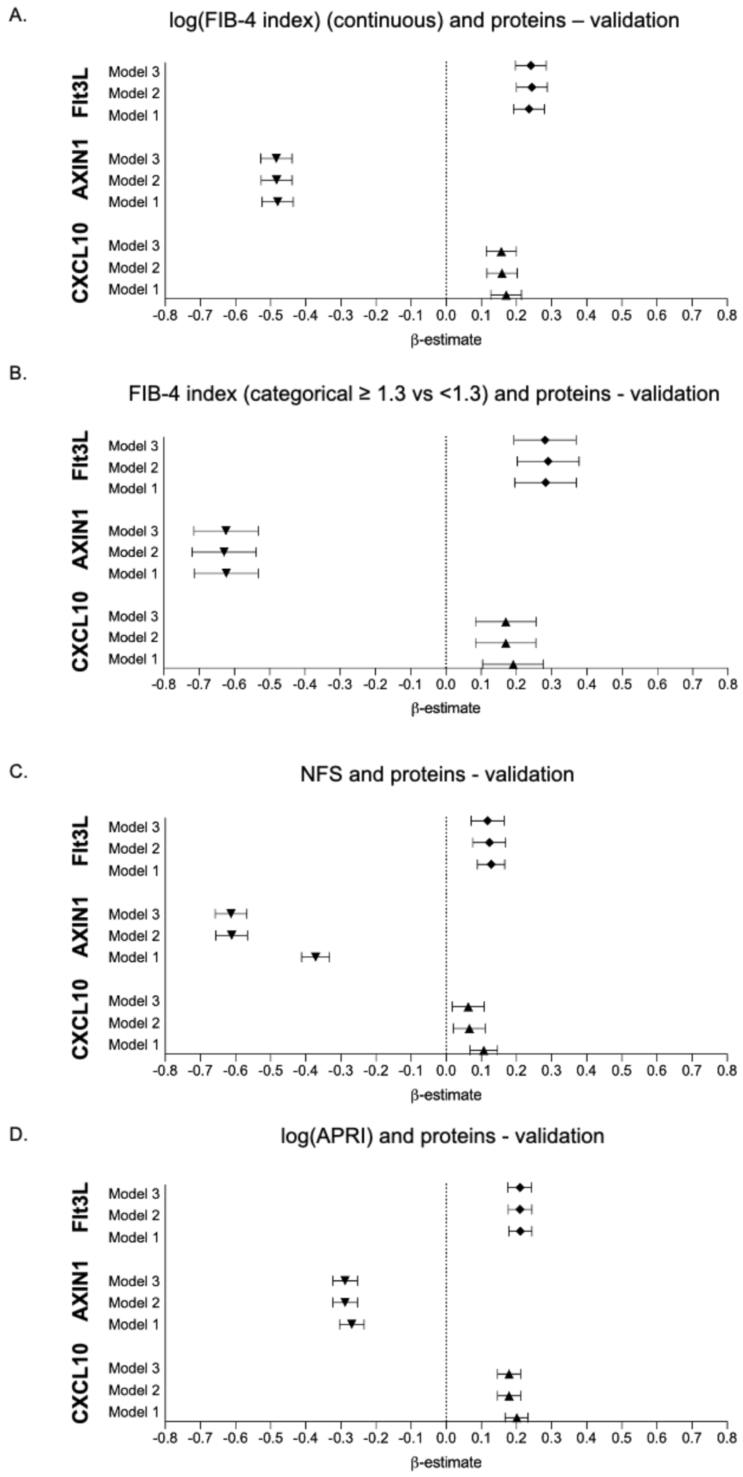

A. log(FIB-4 index) (continuous) and proteins – validation

		β	L 95%CI	U 95%CI	p-value
Flt3L	Model 3	0.241	0.197	0.285	**<0.0001**
	Model 2	0.244	0.200	0.288	**<0.0001**
	Model 1	0.236	0.192	0.280	**<0.0001**
AXIN1	Model 3	-0.484	-0.528	-0.439	**<0.0001**
	Model 2	-0.483	-0.527	-0.439	**<0.000**
	Model 1	-0.480	-0.524	-0.436	**<0.0001**
CXCL10	Model 3	0.157	0.114	0.200	**<0.0001**
	Model 2	0.159	0.116	0.202	**<0.0001**
	Model 1	0.171	0.127	0.214	**<0.0001**

B. FIB-4 index (categorical ≥ 1.3 vs <1.3) and proteins - validation

		β	L 95%CI	U 95%CI	p-value
Flt3L	Model 3	0.281	0.193	0.369	**<0.0001**
	Model 2	0.290	0.203	0.378	**<0.0001**
	Model 1	0.283	0.195	0.370	**<0.0001**
AXIN1	Model 3	-0.624	-0.715	-0.532	**<0.0001**
	Model 2	-0.629	-0.720	-0.538	**<0.0001**
	Model 1	-0.623	-0.714	-0.532	**<0.0001**
CXCL10	Model 3	0.170	0.084	0.256	**0.00010**
	Model 2	0.170	0.084	0.255	**<0.0001**
	Model 1	0.191	0.104	0.277	**<0.0001**

C. NFS and proteins - validation

		β	L 95%CI	U 95%CI	p-value
Flt3L	Model 3	0.118	0.071	0.165	**<0.0001**
	Model 2	0.123	0.076	0.169	**<0.0001**
	Model 1	0.128	0.089	0.167	**<0.0001**
AXIN1	Model 3	-0.613	-0.658	-0.568	**<0.0001**
	Model 2	-0.611	-0.656	-0.566	**<0.0001**
	Model 1	-0.372	-0.412	-0.332	**<0.0001**
CXCL10	Model 3	0.063	0.017	0.108	**0.0071**
	Model 2	0.066	0.020	0.111	**0.0046**
	Model 1	0.107	0.068	0.146	**<0.0001**

D. log(APRI) and proteins - validation

		β	L 95%CI	U 95%CI	p-value
Flt3L	Model 3	0.210	0.176	0.243	**<0.0001**
	Model 2	0.210	0.177	0.244	**<0.0001**
	Model 1	0.211	0.178	0.244	**<0.0001**
AXIN1	Model 3	-0.288	-0.323	-0.253	**<0.0001**
	Model 2	-0.288	-0.323	-0.253	**<0.0001**
	Model 1	-0.269	-0.304	-0.234	**<0.0001**
CXCL10	Model 3	0.179	0.146	0.212	**<0.0001**
	Model 2	0.179	0.146	0.212	**<0.0001**
	Model 1	0.201	0.168	0.233	**<0.0001**

Fig. S9. Validation of the relationship between circulating proteins and non-invasive tests for liver fibrosis at 5 years follow-up. (A) Relationship between log(FIB-4) (per SD) and circulating proteins (per SD) (symbols represent β-estimates and bars represent 95% CIs). Levels of significance: exact p-values on the figure, $p < 0.05$ is considered as statistically significant, significant values in bold (multivariate linear regression, t-test; model 1: N = 3103; model 2: N = 3093; model 3: N = 3075). (B) Relationship between the FIB-4 index (categorical ≥ 1.3 vs <1.3) and circulating proteins (per SD) (symbols represent β-estimates and bars represent 95% CIs). Levels of significance: exact p-values on

the figure, $p < 0.05$ is considered as statistically significant, significant values in bold (multivariate linear regression, t-test; model 1: N = 3103; model 2: N = 3093; model 3: N = 3075). (C) Relationship between the NFS (per SD) and circulating proteins (per SD) (symbols represent β-estimates and bars represent 95% CIs). Levels of significance: exact p-values on the figure, $p < 0.05$ is considered as statistically significant, significant values in bold (multivariate linear regression, t-test; model 1: N = 3095; model 2: N = 3093; model 3: N = 3075). (D) Relationship between log(APRI) (per SD) and circulating proteins (per SD) (symbols represent β-estimates and bars represent 95% CIs). Levels of significance: exact p-values on the figure, $p < 0.05$ is considered as statistically significant, significant values in bold (multivariate linear regression, t-test; model 1: N = 3103; model 2: N = 3093; model 3: N = 3075). [model 1: adjusted for age and sex; model 2: additional adjustment for smoking, arterial hypertension, diabetes mellitus, obesity, and dyslipidemia; model 3: additional adjustment for coronary artery disease and congestive heart failure] [Abbreviations: APRI, AST to platelet index; CI, confidence inteval; L, lower; NFS, NAFLD fibrosis score; U, upper; SD, standard deviation]

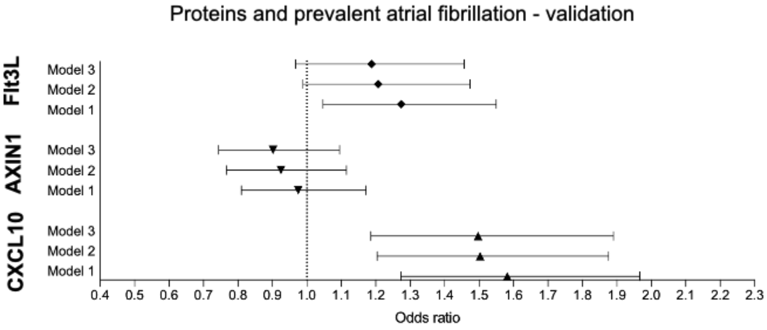

OR	L 95%CI	U 95%CI	p-value
1.188	0.968	1.457	0.099
1.207	0.989	1.474	0.065
1.274	1.047	1.550	**0.016**
0.902	0.744	1.095	0.30
0.925	0.767	1.115	0.41
0.975	0.810	1.172	0.79
1.497	1.186	1.890	**0.00068**
1.503	1.204	1.876	**0.00032**
1.582	1.273	1.967	**<0.0001**

Fig. S10. Validation of the relationship between circulating proteins and prevalent atrial fibrillation at 5 years follow-up. Relationship between circulating proteins (per SD) and atrial fibrillation (symbols represent odds ratios and bars represent 95% CIs). Levels of significance: exact p-values on the figure, $p < 0.05$ is considered as statistically significant, significant values in bold (multivariate logistic regression, z-test; model 1: N = 3100 (116 events); model 2: N = 3090 (116 events); model 3: N = 3073 (113 events)). [model 1: adjusted for age and sex; model 2: additional adjustment for smoking, arterial hypertension, diabetes mellitus, obesity, and dyslipidemia; model 3: additional adjustment for coronary artery disease and congestive heart failure] [Abbreviations: CI, confidence inteval; L, lower; OR, odds ratio; U, upper; SD, standard deviation]

4. Supplementary tables

Table S1: Proteins in the Olink inflammation panel (92 targets)

Protein	UniProt number
Adenosine Deaminase (ADA)	P00813
Artemin (ARTN)	Q5T4W7
Axis inhibition protein 1 (AXIN1)	O15169
Beta-nerve growth factor (Beta-NGF)	P01138
Caspase 8 (CASP-8)	Q14790
C-C motif chemokine 4 (CCL4)	P13236
C-C motif chemokine 19 (CCL19)	Q99731
C-C motif chemokine 20 (CCL20)	P78556
C-C motif chemokine 23 (CCL23)	P55773
C-C motif chemokine 25 (CCL25)	O15444
C-C motif chemokine 28 (CCL28)	Q9NRJ3
CD40L receptor (CD40)	P25942
CUB domain-containing protein 1 (CDCP1)	Q9H5V8
C-X-C motif chemokine 1 (CXCL1)	P09341
C-X-C motif chemokine 5 (CXCL5)	P42830
C-X-C motif chemokine 6 (CXCL6)	P80162
C-X-C motif chemokine 9 (CXCL9)	Q07325
C-X-C motif chemokine 10 (CXCL10)	P02778
C-X-C motif chemokine 11 (CXCL11)	O14625
Cystatin D (CST5)	P28325
Delta and Notch-like epidermal growth factor-related recep (DNER)	Q8NFT8
Eotaxin-1 (CCL11)	P51671
Eukaryotic translation initiation factor 4E-binding protein 1 (4E-BP1)	Q13541
Fibroblast growth factor 5 (FGF-5)	P12034
Fibroblast growth factor 19 (FGF-19)	O95750
Fibroblast growth factor 21 (FGF-21)	Q9NSA1
Fibroblast growth factor 23 (FGF-23)	Q9GZV9
Fms-related tyrosine kinase 3 ligand (Flt3L)	P49771
Fractalkine (CX3CL1)	P78423
Glial cell line-derived neurotrophic factor (GDNF)	P39905
Hepatocyte growth factor (HGF)	P14210
Interferon gamma (IFN-gamma)	P01579
Interleukin-1 alpha (IL-1 alpha)	P01583
Interleukin-2 (IL-2)	P60568
Interleukin-2 receptor subunit beta (IL-2RB)	P14784
Interleukin-4 (IL-4)	P05112
Interleukin-5 (IL-5)	P05113
Interleukin-6 (IL-6)	P05231
Interleukin-7 (IL-7)	P13232
Interleukin-8 (IL-8)	P10145
Interleukin-10 (IL-10)	P22301
Interleukin-10 receptor subunit alpha (IL-10RA)	Q13651
Interleukin-10 receptor subunit beta (IL-10RB)	Q08334
Interleukin-12 subunit beta (IL-12B)	P29460
Interleukin-13 (IL-13)	P35225
Interleukin-15 receptor subunit alpha (IL-15RA)	Q13261
Interleukin-17A (IL-17A)	Q16552
Interleukin-17C (IL-17C)	Q9P0M4
Interleukin-18 (IL-18)	Q14116
Interleukin-18 receptor 1 (IL-18R1)	Q13478
Interleukin-20 (IL-20)	Q9NYY1
Interleukin-20 receptor subunit alpha (IL-20RA)	Q9UHF4
Interleukin-22 receptor subunit alpha-1 (IL-22 RA1)	Q8N6P7
Interleukin-24 (IL-24)	Q13007
Interleukin-33 (IL-33)	O95760
Latency-associated peptide transforming growth factor beta 1 (LAP TGF-beta-1)	P01137
Leukemia inhibitory factor (LIF)	P15018

Leukemia inhibitory factor receptor (LIF-R)	P42702
Macrophage colony-stimulating factor 1 (CSF-1)	P09603
Macrophage inflammatory protein 1-alpha (CCL3)	P10147
Matrix metalloproteinase-1 (MMP-1)	P03956
Matrix metalloproteinase-10 (MMP-10)	P09238
Monocyte chemotactic protein 1 (MCP-1)	P13500
Monocyte chemotactic protein 2 (MCP-2)	P80075
Monocyte chemotactic protein 3 (MCP-3)	P80098
Monocyte chemotactic protein 4 (MCP-4)	Q99616
Natural killer cell receptor 2B4 (CD244)	Q9BZW8
Neurotrophin-3 (NT-3)	P20783
Neurturin (NRTN)	Q99748
Oncostatin-M (OSM)	P13725
Osteoprotegerin (OPG)	O00300
Programmed cell death 1 ligand 1 (PD-L1)	Q9NZQ7
Protein S100-A12 (EN-RAGE)	P80511
Signaling lymphocytic activation molecule (SLAMF1)	Q13291
SIR2-like protein 2 (SIRT2)	Q8IXJ6
STAM-binding protein (STAMPB)	O95630
Stem cell factor (SCF)	P21583
Sulfotransferase 1A1 (ST1A1)	P50225
T-cell surface glycoprotein CD5 (CD5)	P06127
T-cell surface glycoprotein CD6 isoform (CD6)	P30203
T-cell surface glycoprotein CD8 alpha chain (CD8A)	P01732
Thymic stromal lymphopoietin (TSLP)	Q969D9
TNF-beta (TNFB)	P01374
TNF-related activation-induced cytokine (TRANCE)	O14788
TNF-related apoptosis-inducing ligand (TRAIL)	P50591
Transforming growth factor alpha (TGF-alpha)	P01135
Tumor necrosis factor (Ligand) superfamily, member 12 (TWEAK)	O43508
Tumor necrosis factor (TNF)	P01375
Tumor necrosis factor ligand superfamily member 14 (TNFSF14)	O43557
Tumor necrosis factor receptor superfamily member 9 (TNFRSF9)	Q07011
Urokinase-type plasminogen activator (uPA)	P00749
Vascular endothelial growth factor A (VEGF-A)	P15692

Table S2 A. Multivariate logistic regression fibrosis-4 index (continuous per SD) – atrial fibrillation

(Level of significance: $p < 0.05$ is considered as statistically significant (z-test), in bold)

**Model 1**	AUC	N	Odds ratio	L 95%CI	U 95%CI	p-value
Atrial fibrillation	0.7734	11395 (275 events)				
FIB-4 [SD]			1.125	1.038	1.219	**0.0040**
Age [y]			1.094	1.078	1.109	**<0.0001**
Sex (Women)			0.524	0.406	0.675	**<0.0001**
**Model 2**	AUC	N	Odds ratio	L 95%CI	U 95%CI	p-value
Atrial fibrillation	0.7834	11335 (274 events)				
FIB-4 [SD]			1.126	1.039	1.221	**0.0038**
Age [y]			1.086	1.069	1.103	**<0.0001**
Sex (Women)			0.552	0.427	0.715	**<0.0001**
Smoking			0.931	0.636	1.362	0.71
Arterial hypertension			1.079	0.816	1.427	0.59
Diabetes mellitus			1.067	0.761	1.497	0.71
Obesity			1.212	0.926	1.588	0.16
Dyslipidemia			1.602	1.242	2.066	**0.00029**
**Model 3**	AUC	N	Odds ratio	L 95%CI	U 95%CI	p-value
Atrial fibrillation	0.8056	11186 (262 events)				
FIB-4 [SD]			1.100	1.011	1.196	**0.026**
Age [y]			1.077	1.060	1.094	**<0.0001**
Sex (Women)			0.551	0.420	0.724	**<0.0001**
Smoking			0.991	0.674	1.456	0.96
Arterial hypertension			0.998	0.747	1.334	0.99
Diabetes mellitus			1.040	0.727	1.488	0.83
Obesity			1.117	0.840	1.485	0.45
Dyslipidemia			1.414	1.075	1.861	**0.013**
Congestive heart failure			7.871	5.039	12.296	**<0.0001**
Coronary artery disease			1.935	1.330	2.814	**0.00056**

Table S2 B. Multivariate logistic regression fibrosis-4 index (categorical <1.3 / ≥ 1.3) – atrial fibrillation

(Level of significance: $p < 0.05$ is considered as statistically significant (z-test), in bold)

**Model 1**	AUC	N	Odds ratio	L 95%CI	U 95%CI	p-value
Atrial fibrillation	0.7735	11395 (275 events)				
FIB-4 ≥ 1.3			1.463	1.109	1.930	**0.0072**
Age [y]			1.092	1.076	1.108	**<0.0001**
Sex (Women)			0.534	0.413	0.689	**<0.0001**
**Model 2**	AUC	N	Odds ratio	L 95%CI	U 95%CI	p-value
Atrial fibrillation	0.7841	11335 (274 events)				
FIB-4 ≥ 1.3			1.489	1.127	1.966	**0.0050**
Age [y]			1.084	1.067	1.101	**<0.0001**
Sex (Women)			0.564	0.435	0.732	**<0.0001**
Smoking			0.929	0.635	1.361	0.71
Arterial hypertension			1.071	0.810	1.416	0.63
Diabetes mellitus			1.070	0.763	1.500	0.70
Obesity			1.227	0.937	1.606	0.14
Dyslipidemia			1.607	1.246	2.073	**0.00026**
**Model 3**	AUC	N	Odds ratio	L 95%CI	U 95%CI	p-value
Atrial fibrillation	0.8059	11186 (262 events)				
FIB-4 ≥ 1.3			1.363	1.017	1.826	**0.038**
Age [y]			1.076	1.058	1.093	**<0.0001**
Sex (Women)			0.560	0.426	0.737	**<0.0001**
Smoking			0.987	0.671	1.451	0.95
Arterial hypertension			0.990	0.741	1.323	0.95
Diabetes mellitus			1.043	0.729	1.492	0.82
Obesity			1.126	0.847	1.497	0.42
Dyslipidemia			1.411	1.072	1.857	**0.014**
Congestive heart failure			7.818	5.000	12.223	**<0.0001**
Coronary artery disease			1.952	1.343	2.837	**0.00045**

Table S3. Study participants characteristics stratified by the fibrosis-4 index at 5 years follow-up. Data presented as mean +/- standard deviation (Gaussian-distributed data), median with interquartile range (non-Gaussian distributed data), or as relative and absolute frequencies (categorical data); a two-sided t-test was used for comparing two Gaussian-distributed continuous variables, a Wilcoxon rank sum test for non-Gaussian distributed variables, and a chi-square for categorical data; Level of significance: a p-value < 0.05 is considered as statistically significant, in bold.

**Variable**	**Whole sample (9300)**	**Fibrosis-4 index < 1.3 (6041)**	**Fibrosis-4 index ≥ 1.3 (3259)**	**p-value**
		**Demographics**		
Sex (Women)	50.3% (4677)	53.7% (3243)	44.0% (1434)	**<0.0001**
Age [y]	58.2 +/- 10.8	54.3 +/- 9.4	65.5 +/- 9.3	**<0.0001**
BMI [kg/m^2]	26.7 (24.0/30.3)	26.6 (23.9/30.1)	27.0 (24.2/30.5)	**0.00028**
		**Cardiovascular risk factors**		
Dyslipidaemia	33.6% (3122)	30.5% (1843)	39.3% (1279)	**<0.0001**
Arterial hypertension	56.1% (5214)	49.4% (2984)	68.4% (2230)	**<0.0001**
Smoking	15.3% (1420)	18.0% (1087)	10.2% (333)	**<0.0001**
Obesity	26.6% (2474)	25.8% (1559)	28.1% (915)	**0.019**
Family history of myocardial infarction/stroke	23.8% (2215)	24.1% (1458)	23.2% (757)	0.33
Diabetes mellitus	11.0% (1019)	9.1% (548)	14.5% (471)	**<0.0001**
		**Comorbidities**		
Coronary artery disease	4.4% (406)	2.3% (139)	8.3% (267)	**<0.0001**
Myocardial infarction	1.1% (100)	0.6% (38)	1.9% (62)	**<0.0001**
Peripheral artery disease	1.7% (152)	1.1% (64)	2.8% (88)	**<0.0001**
Atrial fibrillation	3.5% (325)	1.7% (104)	6.8% (221)	**<0.0001**
Congestive heart failure	2.0% (189)	1.1% (66)	3.8% (123)	**<0.0001**
Chronic kidney disease	0.8% (78)	0.7% (42)	1.1% (36)	**0.043**
		**Liver parameters**		
Fatty liver index	48.30 +/- 29.96	46.50 +/- 29.94	52.73 +/- 29.57	**<0.0001**
Fatty liver index ≥ 60	38.2% (2395)	35.8% (1598)	43.9% (797)	**<0.0001**
Fibrosis-4 index	1.11 (0.86/1.47)	0.93 (0.75/1.10)	1.63 (1.44/1.98)	**<0.0001**
NAFLD fibrosis score	-1.83 +/- 1.29	-2.39 +/- 1.06	-0.78 +/- 0.97	**<0.0001**
AST to platelet index	0.32 (0.26/0.41)	0.29 (0.24/0.34)	0.42 (0.35/0.52)	**<0.0001**
Alanine aminotransferase [U/L]	30.00 (24.00/39.00)	31.00 (25.00/40.00)	29.00 (22.00/38.00)	**<0.0001**
Aspartate aminotransferase [U/L]	25.0 (22.0/30.0)	24.0 (21.0/28.0)	28.0 (24.0/33.0)	**<0.0001**
Gamma-glutamyl transferase [U/L]	24.00 (17.00/36.00)	24.00 (17.00/35.00)	25.00 (18.00/38.83)	**<0.0001**

Table S4 A. Cox competing risk analysis for log(fibrosis-4 index) (continuous per SD) and incident atrial fibrillation (event = atrial fibrillation, competing event = death)

(Level of significance: $p < 0.05$ is considered as statistically significant (z-test), in bold)

**Model 1**	Sample	HR	L95%CI	U95%CI	p-value
Atrial fibrillation ~	C-index: 0.7616, N:10796 (246 events, 166 competing events)				
log(FIB-4) [SD]		1.136	0.953	1.353	0.15
Age [y]		1.088	1.070	1.107	**<0.0001**
Sex (Women)		0.665	0.514	0.862	**0.0020**
**Model 2**	Sample	HR	L95%CI	U95%CI	p-value
Atrial fibrillation ~	C-index: 0.7739, N:10737 (246 events, 164 competing events)				
log(FIB-4) [SD]		1.157	0.977	1.370	0.091
Age [y]		1.080	1.061	1.100	**<0.0001**
Sex (Women)		0.708	0.545	0.919	**0.0096**
Smoking		1.236	0.860	1.777	0.25
Arterial hypertension		1.157	0.863	1.551	0.33
Diabetes mellitus		1.469	1.056	2.043	**0.022**
Obesity		1.391	1.060	1.824	**0.017**
Dyslipidemia		1.240	0.957	1.606	0.10
**Model 3**	Sample	HR	L95%CI	U95%CI	p-value
Atrial fibrillation ~	C-index: 0.7738, N:10591 (232 events, 159 competing events)				
log(FIB-4) [SD]		1.125	0.943	1.342	0.19
Age [y]		1.081	1.061	1.102	**<0.0001**
Sex (Women)		0.723	0.550	0.950	**0.020**
Smoking		1.260	0.871	1.825	0.22
Arterial hypertension		1.107	0.819	1.495	0.51
Diabetes mellitus		1.279	0.895	1.827	0.18
Obesity		1.303	0.980	1.733	0.069
Dyslipidemia		1.159	0.880	1.527	0.29
Congestive heart failure		2.537	1.369	4.701	**0.0031**
Coronary artery disease		1.306	0.844	2.021	0.23

Table S4 B. Cox competing risk analysis for fibrosis-4 index (categorical <1.3 / ≥ 1.3) and incident atrial fibrillation (event = atrial fibrillation, competing event = death)

(Level of significance: $p < 0.05$ is considered as statistically significant (z-test), in bold)

**Model 1**	Sample	HR	L95%CI	U95%CI	p-value
Atrial fibrillation ~	C-index: 0.7613, N:10796 (246 events, 166 competing events)				
FIB-4 (≥1.3)		1.155	0.863	1.545	0.33
Age [y]		1.093	1.078	1.109	**<0.0001**
Sex (Women)		0.652	0.505	0.841	**0.0010**
**Model 2**	Sample	HR	L95%CI	U95%CI	p-value
Atrial fibrillation ~	C-index: 0.7732, N:10737 (246 events, 164 competing events)				
FIB-4 (≥1.3)		1.179	0.880	1.578	0.27
Age [y]		1.08	1.069	1.103	**<0.0001**
Sex (Women)		0.69	0.534	0.895	**0.0051**
Smoking		1.214	0.845	1.743	0.29
Arterial hypertension		1.154	0.861	1.548	0.34
Diabetes mellitus		1.46	1.052	2.034	**0.024**
Obesity		1.393	1.063	1.826	**0.016**
Dyslipidemia		1.236	0.955	1.599	0.11
**Model 3**	Sample	HR	L95%CI	U95%CI	p-value
Atrial fibrillation ~	C-index: 0.7730, N:10591 (232 events, 159 competing events)				
FIB-4 (≥1.3)		1.098	0.809	1.490	0.55
Age [y]		1.087	1.069	1.105	**<0.0001**
Sex (Women)		0.705	0.539	0.923	**0.011**
Smoking		1.237	0.855	1.788	0.26
Arterial hypertension		1.104	0.817	1.491	0.52
Diabetes mellitus		1.270	0.889	1.815	0.19
Obesity		1.303	0.981	1.732	0.068
Dyslipidemia		1.153	0.877	1.516	0.31
Congestive heart failure		2.565	1.382	4.759	**0.0028**
Coronary artery disease		1.327	0.859	2.052	0.20

Table S5 A. Multivariate linear regression for log(fibrosis-4 index) (continuous per SD) and log(NT-proBNP) (continuous per SD)

(Level of significance: $p < 0.05$ is considered as statistically significant (t-test), in bold)

**Model 1**	R^2	N	Estimate	L 95% CI	U 95% CI	p-value
log(NT-proBNP) [SD]	0.2756	11114				
log(FIB-4) [SD]			0.128	0.106	0.149	**<0.0001**
Age [y]			0.031	0.029	0.033	**<0.0001**
Sex (Women)			0.621	0.588	0.653	**<0.0001**
**Model 2**	R^2	N	Estimate	L 95% CI	U 95% CI	p-value
log(NT-proBNP) [SD]	0.2296	11054				
log(FIB-4) [SD]			0.129	0.107	0.151	**<0.0001**
Age [y]			0.030	0.028	0.032	**<0.0001**
Sex (Women)			0.615	0.582	0.648	**<0.0001**
Smoking			0.049	0.008	0.090	**0.020**
Arterial hypertension			0.088	0.052	0.124	**<0.0001**
Diabetes mellitus			0.111	0.052	0.170	**0.00022**
Obesity			-0.053	-0.092	-0.015	**0.0064**
Dyslipidemia			-0.089	-0.125	-0.054	**<0.0001**
**Model 3**	R^2	N	Estimate	L 95% CI	U 95% CI	p-value
log(NT-proBNP) [SD]	0.3010	10891				
log(FIB-4) [SD]			0.117	0.095	0.138	**<0.0001**
Age [y]			0.029	0.027	0.031	**<0.0001**
Sex (Women)			0.633	0.600	0.665	**<0.0001**
Smoking			0.055	0.015	0.096	**0.0071**
Arterial hypertension			0.076	0.041	0.112	**<0.0001**
Diabetes mellitus			0.055	-0.005	0.114	0.071
Obesity			-0.074	-0.112	-0.036	**0.00013**
Dyslipidemia			-0.137	-0.173	-0.101	**<0.0001**
Congestive heart failure			0.727	0.583	0.871	**<0.0001**
Coronary artery disease			0.590	0.506	0.674	**<0.0001**

Table S5 B. Multivariate linear regression for fibrosis-4 index (categorical <1.3 / ≥ 1.3) and log(NT-proBNP) (continuous per SD)

(Level of significance: $p < 0.05$ is considered as statistically significant (t-test), in bold)

**Model 1**	R^2	N	Estimate	L 95% CI	U 95% CI	p-value
log(NT-proBNP) [SD]						
FIB-4 (≥1.3)	0.2790	11114	0.340	0.291	0.389	**<0.0001**
Age [y]			0.034	0.032	0.036	**<0.0001**
Sex (Women)			0.610	0.578	0.642	**<0.0001**
**Model 2**	R^2	N	Estimate	L 95% CI	U 95% CI	p-value
log(NT-proBNP) [SD]	0.2827	11054				
FIB-4 (≥1.3)			0.337	0.288	0.386	**<0.0001**
Age [y]			0.033	0.032	0.035	**<0.0001**
Sex (Women)			0.602	0.569	0.634	**<0.0001**
Smoking			0.039	-0.002	0.080	0.060
Arterial hypertension			0.084	0.048	0.120	**<0.0001**
Diabetes mellitus			0.102	0.043	0.161	**0.00067**
Obesity			-0.056	-0.094	-0.018	**0.0042**
Dyslipidemia			-0.093	-0.129	-0.058	**<0.0001**
**Model 3**	R^2	N	Estimate	L 95% CI	U 95% CI	p-value
log(NT-proBNP) [SD]	0.3035	10891				
FIB-4 (≥1.3)			0.305	0.256	0.354	**<0.0001**
Age [y]			0.032	0.030	0.033	**<0.0001**
Sex (Women)			0.621	0.588	0.653	**<0.0001**
Smoking			0.046	0.006	0.087	**0.023**
Arterial hypertension			0.072	0.037	0.108	**<0.0001**
Diabetes mellitus			0.049	-0.010	0.108	0.11
Obesity			-0.077	-0.115	-0.039	**<0.0001**
Dyslipidemia			-0.141	-0.176	-0.105	**<0.0001**
Congestive heart failure			0.721	0.578	0.865	**<0.0001**
Coronary artery disease			0.583	0.500	0.667	**<0.0001**

Table S6 A. Multivariate linear regression for log(fibrosis-4 index) (continuous per SD) and proteins (per SD)

(Level of significance: $p < 0.05$ is considered as statistically significant (t-test), in bold)

**Model 1**	R^2	N	Estimate	L 95% CI	U 95% CI	p-value
P78556 (CCL20) [SD]	0.0222	5741				
log(FIB-4) [SD]			0.041	0.006	0.075	**0.022**
Age [y]			0.011	0.007	0.014	**<0.0001**
Sex (Women)			-0.068	-0.120	-0.016	**0.011**
**Model 2**	R^2	N	Estimate	L 95% CI	U 95% CI	p-value
P78556 (CCL20) [SD]	0.0700	5704				
log(FIB-4) [SD]			0.068	0.034	0.103	**<0.0001**
Age [y]			0.005	0.001	0.008	**0.0054**
Sex (Women)			0.004	-0.048	0.057	0.87
Smoking			0.325	0.260	0.391	**<0.0001**
Arterial hypertension			0.114	0.058	0.171	**<0.0001**
Diabetes mellitus			0.274	0.202	0.346	**<0.0001**
Obesity			0.183	0.125	0.241	**<0.0001**
Dyslipidemia			0.115	0.060	0.169	**<0.0001**
**Model 3**	R^2	N	Estimate	L 95% CI	U 95% CI	p-value
P78556 (CCL20) [SD]	0.0709	5601				
log(FIB-4) [SD]			0.071	0.036	0.105	**<0.0001**
Age [y]			0.004	0.001	0.008	**0.012**
Sex (Women)			-0.002	-0.055	0.051	0.95
Smoking			0.325	0.259	0.390	**<0.0001**
Arterial hypertension			0.114	0.058	0.171	**<0.0001**
Diabetes mellitus			0.276	0.203	0.350	**<0.0001**
Obesity			0.175	0.117	0.234	**<0.0001**
Dyslipidemia			0.110	0.054	0.166	**0.00011**
Congestive heart failure			0.319	0.129	0.508	**0.00098**
Coronary artery disease			-0.049	-0.157	0.060	0.38
**Model 1**	R^2	N	Estimate	L 95% CI	U 95% CI	p-value
Q8NFT8 (DNER) [SD]	0.0373	5741				
log(FIB-4) [SD]			0.036	0.002	0.071	**0.041**
Age [y]			-0.018	-0.021	-0.015	**<0.0001**
Sex (Women)			-0.172	-0.223	-0.120	**<0.0001**
**Model 2**	R^2	N	Estimate	L 95% CI	U 95% CI	p-value
Q8NFT8 (DNER) [SD]	0.0750	5704				
log(FIB-4) [SD]			0.029	-0.005	0.064	0.091
Age [y]			-0.016	-0.019	-0.013	**<0.0001**
Sex (Women)			-0.190	-0.242	-0.137	**<0.0001**
Smoking			-0.024	-0.089	0.041	0.48
Arterial hypertension			0.019	-0.037	0.076	0.50
Diabetes mellitus			0.110	0.038	0.182	**0.0027**
Obesity			-0.429	-0.486	-0.371	**<0.0001**
Dyslipidemia			-0.056	-0.110	-0.001	**0.045**
**Model 3**	R^2	N	Estimate	L 95% CI	U 95% CI	p-value
Q8NFT8 (DNER) [SD]	0.0940	5601				
log(FIB-4) [SD]			0.040	0.006	0.074	**0.021**
Age [y]			-0.014	-0.017	-0.011	**<0.0001**

Sex (Women)			-0.215	-0.267	-0.163	**<0.0001**
Smoking			-0.029	-0.094	0.036	0.38
Arterial hypertension			0.035	-0.021	0.091	0.22
Diabetes mellitus			0.125	0.052	0.197	**0.00074**
Obesity			-0.409	-0.466	-0.351	**<0.0001**
Dyslipidemia			-0.003	-0.059	0.052	0.91
Congestive heart failure			-0.403	-0.591	-0.216	**<0.0001**
Coronary artery disease			-0.537	-0.645	-0.430	**<0.0001**
**Model 1**	R^2	N	Estimate	L 95% CI	U 95% CI	p-value
P02778 (CXCL10) [SD]	0.1217	5741				
log(FIB-4) [SD]			0.180	0.147	0.213	**<0.0001**
Age [y]			0.019	0.016	0.022	**<0.0001**
Sex (Women)			0.146	0.096	0.195	**<0.0001**
**Model 2**	R^2	N	Estimate	L 95% CI	U 95% CI	p-value
P02778 (CXCL10) [SD]	0.1488	5704				
log(FIB-4) [SD]			0.165	0.132	0.197	**<0.0001**
Age [y]			0.016	0.013	0.019	**<0.0001**
Sex (Women)			0.143	0.092	0.193	**<0.0001**
Smoking			-0.340	-0.402	-0.277	**<0.0001**
Arterial hypertension			0.035	-0.019	0.089	0.20
Diabetes mellitus			-0.059	-0.128	0.010	0.093
Obesity			0.196	0.140	0.251	**<0.0001**
Dyslipidemia			0.059	0.007	0.112	**0.026**
**Model 3**	R^2	N	Estimate	L 95% CI	U 95% CI	p-value
P02778 (CXCL10) [SD]	0.1468	5601				
log(FIB-4) [SD]			0.160	0.127	0.194	**<0.0001**
Age [y]			0.016	0.013	0.019	**<0.0001**
Sex (Women)			0.142	0.091	0.193	**<0.0001**
Smoking			-0.331	-0.394	-0.268	**<0.0001**
Arterial hypertension			0.034	-0.020	0.089	0.22
Diabetes mellitus			-0.062	-0.132	0.009	0.085
Obesity			0.188	0.132	0.244	**<0.0001**
Dyslipidemia			0.055	0.001	0.108	**0.047**
Congestive heart failure			0.158	-0.024	0.340	0.089
Coronary artery disease			0.052	-0.052	0.157	0.33
**Model 1**	R^2	N	Estimate	L 95% CI	U 95% CI	p-value
O15169 (AXIN1) [SD]	0.1158	5741				
log(FIB-4) [SD]			-0.457	-0.490	-0.424	**<0.0001**
Age [y]			0.022	0.019	0.025	**<0.0001**
Sex (Women)			-0.151	-0.201	-0.101	**<0.0001**
**Model 2**	R^2	N	Estimate	L 95% CI	U 95% CI	p-value
O15169 (AXIN1) [SD]	0.1305	5704				
log(FIB-4) [SD]			-0.457	-0.490	-0.424	**<0.0001**
Age [y]			0.016	0.013	0.020	**<0.0001**
Sex (Women)			-0.119	-0.170	-0.068	**<0.0001**
Smoking			-0.124	-0.187	-0.060	**0.00013**
Arterial hypertension			0.095	0.040	0.150	**0.00065**
Diabetes mellitus			0.184	0.115	0.254	**<0.0001**
Obesity			0.062	0.006	0.118	**0.030**
Dyslipidemia			0.088	0.035	0.141	**0.0012**

**Model 3**	R^2	N	Estimate	L 95% CI	U 95% CI	p-value
O15169 (AXIN1) [SD]	0.1337	5601				
log(FIB-4) [SD]			-0.451	-0.485	-0.418	**<0.0001**
Age [y]			0.017	0.014	0.020	**<0.0001**
Sex (Women)			-0.128	-0.179	-0.077	**<0.0001**
Smoking			-0.123	-0.187	-0.060	**0.00014**
Arterial hypertension			0.096	0.041	0.150	**0.00066**
Diabetes mellitus			0.191	0.120	0.262	**<0.0001**
Obesity			0.073	0.017	0.130	**0.011**
Dyslipidemia			0.112	0.057	0.166	**<0.0001**
Congestive heart failure			-0.015	-0.199	0.168	0.87
Coronary artery disease			-0.262	-0.368	-0.157	**<0.0001**
**Model 1**	R^2	N	Estimate	L 95% CI	U 95% CI	p-value
P49771 (Flt3L) [SD]	0.1105	5741				
log(FIB-4) [SD]			0.176	0.143	0.210	**<0.0001**
Age [y]			0.017	0.014	0.020	**<0.0001**
Sex (Women)			0.255	0.205	0.305	**<0.0001**
**Model 2**	R^2	N	Estimate	L 95% CI	U 95% CI	p-value
P49771 (Flt3L) [SD]	0.1193	5704				
log(FIB-4) [SD]			0.192	0.159	0.225	**<0.0001**
Age [y]			0.016	0.013	0.019	**<0.0001**
Sex (Women)			0.283	0.232	0.334	**<0.0001**
Smoking			0.216	0.153	0.280	**<0.0001**
Arterial hypertension			0.020	-0.035	0.075	0.48
Diabetes mellitus			0.041	-0.029	0.112	0.25
Obesity			0.014	-0.042	0.070	0.62
Dyslipidemia			0.048	-0.005	0.102	0.075
**Model 3**	R^2	N	Estimate	L 95% CI	U 95% CI	p-value
P49771 (Flt3L) [SD]	0.1190	5601				
log(FIB-4) [SD]			0.189	0.155	0.222	**<0.0001**
Age [y]			0.016	0.012	0.019	**<0.0001**
Sex (Women)			0.287	0.235	0.339	**<0.0001**
Smoking			0.223	0.159	0.287	**<0.0001**
Arterial hypertension			0.025	-0.030	0.081	0.37
Diabetes mellitus			0.029	-0.042	0.101	0.42
Obesity			0.003	-0.054	0.060	0.92
Dyslipidemia			0.041	-0.014	0.095	0.15
Congestive heart failure			0.171	-0.014	0.356	0.070
Coronary artery disease			0.030	-0.076	0.137	0.58

Table S6 B. Multivariate linear regression for fibrosis-4 index (categorical <1.3 / ≥ 1.3) and proteins (per SD)

(Level of significance: $p < 0.05$ is considered as statistically significant (t-test), in bold)

**Model 1**	R^2	N	Estimate	L 95% CI	U 95% CI	p-value
P78556 (CCL20) [SD]	0.0215	5741				
FIB-4 (≥1.3)			0.035	-0.040	0.109	0.36
Age [y]			0.012	0.010	0.015	**<0.0001**
Sex (Women)			-0.076	-0.128	-0.025	**0.0038**
**Model 2**	R^2	N	Estimate	L 95% CI	U 95% CI	p-value
P78556 (CCL20) [SD]	0.0679	5704				
FIB-4 (≥1.3)			0.055	-0.018	0.128	0.14
Age [y]			0.008	0.005	0.011	**<0.0001**
Sex (Women)			-0.012	-0.064	0.040	0.66
Smoking			0.314	0.249	0.379	**<0.0001**
Arterial hypertension			0.113	0.057	0.170	**<0.0001**
Diabetes mellitus			0.271	0.199	0.343	**<0.0001**
Obesity			0.182	0.124	0.240	**<0.0001**
Dyslipidemia			0.111	0.057	0.166	**<0.0001**
**Model 3**	R^2	N	Estimate	L 95% CI	U 95% CI	p-value
P78556 (CCL20) [SD]	0.0687	5601				
FIB-4 (≥1.3)			0.065	-0.009	0.139	0.087
Age [y]			0.007	0.005	0.010	**<0.0001**
Sex (Women)			-0.017	-0.070	0.035	0.51
Smoking			0.313	0.248	0.378	**<0.0001**
Arterial hypertension			0.113	0.056	0.170	**<0.0001**
Diabetes mellitus			0.273	0.200	0.347	**<0.0001**
Obesity			0.174	0.116	0.233	**<0.0001**
Dyslipidemia			0.106	0.050	0.162	**0.00020**
Congestive heart failure			0.327	0.137	0.517	**0.00074**
Coronary artery disease			-0.043	-0.151	0.066	0.44
**Model 1**	R^2	N	Estimate	L 95% CI	U 95% CI	p-value
Q8NFT8 (DNER) [SD]	0.0370	5741				
FIB-4 (≥1.3)			-0.055	-0.129	0.019	0.14
Age [y]			-0.015	-0.017	-0.012	**<0.0001**
Sex (Women)			-0.187	-0.238	-0.136	**<0.0001**
**Model 2**	R^2	N	Estimate	L 95% CI	U 95% CI	p-value
Q8NFT8 (DNER) [SD]	0.0750	5704				
FIB-4 (≥1.3)			-0.066	-0.139	0.007	0.075
Age [y]			-0.013	-0.016	-0.011	**<0.0001**
Sex (Women)			-0.205	-0.257	-0.153	**<0.0001**
Smoking			-0.032	-0.097	0.033	0.33
Arterial hypertension			0.019	-0.037	0.075	0.51
Diabetes mellitus			0.110	0.038	0.182	**0.0028**
Obesity			-0.430	-0.488	-0.372	**<0.0001**
Dyslipidemia			-0.058	-0.112	-0.003	**0.038**
**Model 3**	R^2	N	Estimate	L 95% CI	U 95% CI	p-value
Q8NFT8 (DNER) [SD]	0.0934	5601				
FIB-4 (≥1.3)			-0.049	-0.122	0.025	0.20
Age [y]			-0.011	-0.014	-0.008	**<0.0001**

Sex (Women)			-0.231	-0.283	-0.180	**<0.0001**
Smoking			-0.039	-0.104	0.026	0.24
Arterial hypertension			0.034	-0.022	0.090	0.23
Diabetes mellitus			0.123	0.051	0.196	**0.00087**
Obesity			-0.410	-0.468	-0.353	**<0.0001**
Dyslipidemia			-0.007	-0.062	0.048	0.81
Congestive heart failure			-0.389	-0.576	-0.202	**<0.0001**
Coronary artery disease			-0.527	-0.635	-0.420	**<0.0001**
**Model 1**	R^2	N	Estimate	L 95% CI	U 95% CI	p-value
P02778 (CXCL10) [SD]	0.1114	5741				
FIB-4 (≥1.3)			0.251	0.179	0.322	**<0.0001**
Age [y]			0.026	0.024	0.028	**<0.0001**
Sex (Women)			0.116	0.067	0.165	**<0.0001**
**Model 2**	R^2	N	Estimate	L 95% CI	U 95% CI	p-value
P02778 (CXCL10) [SD]	0.1406	5704				
FIB-4 (≥1.3)			0.233	0.162	0.303	**<0.0001**
Age [y]			0.022	0.019	0.025	**<0.0001**
Sex (Women)			0.113	0.063	0.163	**<0.0001**
Smoking			-0.362	-0.425	-0.300	**<0.0001**
Arterial hypertension			0.033	-0.021	0.088	0.23
Diabetes mellitus			-0.067	-0.136	0.002	0.058
Obesity			0.195	0.139	0.250	**<0.0001**
Dyslipidemia			0.052	0.000	0.105	0.051
**Model 3**	R^2	N	Estimate	L 95% CI	U 95% CI	p-value
P02778 (CXCL10) [SD]	0.1387	5601				
FIB-4 (≥1.3)			0.221	0.149	0.293	**<0.0001**
Age [y]			0.022	0.019	0.025	**<0.0001**
Sex (Women)			0.113	0.062	0.163	**<0.0001**
Smoking			-0.354	-0.417	-0.291	**<0.0001**
Arterial hypertension			0.032	-0.023	0.086	0.26
Diabetes mellitus			-0.069	-0.140	0.002	0.057
Obesity			0.186	0.130	0.243	**<0.0001**
Dyslipidemia			0.046	-0.008	0.100	0.092
Congestive heart failure			0.168	-0.015	0.351	0.072
Coronary artery disease			0.061	-0.044	0.166	0.26
**Model 1**	R^2	N	Estimate	L 95% CI	U 95% CI	p-value
O15169 (AXIN1) [SD]	0.0414	5741				
FIB-4 (≥1.3)			-0.576	-0.650	-0.502	**<0.0001**
Age [y]			0.004	0.001	0.006	**0.0049**
Sex (Women)			-0.071	-0.122	-0.020	**0.0066**
**Model 2**	R^2	N	Estimate	L 95% CI	U 95% CI	p-value
O15169 (AXIN1) [SD]	0.0581	5704				
FIB-4 (≥1.3)			-0.580	-0.653	-0.506	**<0.0001**
Age [y]			-0.002	-0.004	0.001	0.24
Sex (Women)			-0.030	-0.082	0.022	0.26
Smoking			-0.057	-0.123	0.008	0.085
Arterial hypertension			0.101	0.044	0.158	**0.00049**
Diabetes mellitus			0.206	0.133	0.278	**<0.0001**
Obesity			0.066	0.008	0.124	**0.027**
Dyslipidemia			0.108	0.053	0.163	**0.00012**

**Model 3**	R^2	N	Estimate	L 95% CI	U 95% CI	p-value
O15169 (AXIN1) [SD]	0.0611	5601				
FIB-4 (≥1.3)			-0.558	-0.633	-0.484	**<0.0001**
Age [y]			-0.001	-0.004	0.002	0.58
Sex (Women)			-0.040	-0.093	0.013	0.14
Smoking			-0.056	-0.122	0.010	0.096
Arterial hypertension			0.103	0.046	0.160	**0.00042**
Diabetes mellitus			0.210	0.136	0.284	**<0.0001**
Obesity			0.079	0.020	0.138	**0.0084**
Dyslipidemia			0.136	0.079	0.192	**<0.0001**
Congestive heart failure			-0.050	-0.241	0.141	0.61
Coronary artery disease			-0.290	-0.400	-0.180	**<0.0001**
**Model 1**	R^2	N	Estimate	L 95% CI	U 95% CI	p-value
P49771 (Flt3L) [SD]	0.0994	5741				
FIB-4 (≥1.3)			0.221	0.150	0.293	**<0.0001**
Age [y]			0.024	0.021	0.026	**<0.0001**
Sex (Women)			0.224	0.174	0.273	**<0.0001**
**Model 2**	R^2	N	Estimate	L 95% CI	U 95% CI	p-value
P49771 (Flt3L) [SD]	0.1058	5704				
FIB-4 (≥1.3)			0.232	0.160	0.303	**<0.0001**
Age [y]			0.024	0.021	0.026	**<0.0001**
Sex (Women)			0.245	0.194	0.295	**<0.0001**
Smoking			0.188	0.124	0.252	**<0.0001**
Arterial hypertension			0.017	-0.038	0.073	0.54
Diabetes mellitus			0.032	-0.038	0.103	0.37
Obesity			0.012	-0.044	0.069	0.67
Dyslipidemia			0.040	-0.014	0.093	0.15
**Model 3**	R^2	N	Estimate	L 95% CI	U 95% CI	p-value
P49771 (Flt3L) [SD]	0.1059	5601				
FIB-4 (≥1.3)			0.225	0.152	0.298	**<0.0001**
Age [y]			0.023	0.021	0.026	**<0.0001**
Sex (Women)			0.250	0.198	0.301	**<0.0001**
Smoking			0.195	0.130	0.259	**<0.0001**
Arterial hypertension			0.022	-0.034	0.078	0.44
Diabetes mellitus			0.021	-0.051	0.093	0.57
Obesity			0.000	-0.057	0.058	0.99
Dyslipidemia			0.030	-0.025	0.085	0.28
Congestive heart failure			0.187	0.000	0.373	0.050
Coronary artery disease			0.043	-0.064	0.150	0.44

Table S7. Multivariate logistic regression for proteins (per SD) and atrial fibrillation

(Level of significance: $p < 0.05$ is considered as statistically significant (z-test), in bold)

**Model 1**	AUC	N	Odds ratio	L 95%CI	U 95%CI	p-value
Atrial fibrillation	0.7698	5672 (172 events)				
P78556 (CCL20) [SD]			1.520	1.326	1.743	**<0.0001**
Age [y]			1.090	1.070	1.110	**<0.0001**
Sex (Women)			0.522	0.378	0.721	**<0.0001**
**Model 2**	AUC	N	Odds ratio	L 95%CI	U 95%CI	p-value
Atrial fibrillation	0.7779	5635 (171 events)				
P78556 (CCL20) [SD]			1.506	1.307	1.735	**<0.0001**
Age [y]			1.082	1.061	1.104	**<0.0001**
Sex (Women)			0.543	0.390	0.756	**0.00030**
Smoking			0.824	0.507	1.340	0.43
Arterial hypertension			1.157	0.800	1.673	0.44
Diabetes mellitus			0.939	0.648	1.361	0.74
Obesity			1.099	0.786	1.536	0.58
Dyslipidemia			1.631	1.173	2.268	**0.0036**
**Model 3**	AUC	N	Odds ratio	L 95%CI	U 95%CI	p-value
Atrial fibrillation	0.8028	5543 (162 events)				
P78556 (CCL20) [SD]			1.435	1.234	1.668	**<0.0001**
Age [y]			1.071	1.049	1.093	**<0.0001**
Sex (Women)			0.538	0.378	0.766	**0.00059**
Smoking			0.912	0.557	1.494	0.71
Arterial hypertension			1.073	0.732	1.573	0.72
Diabetes mellitus			1.004	0.679	1.485	0.98
Obesity			0.985	0.691	1.405	0.93
Dyslipidemia			1.488	1.038	2.133	**0.031**
Congestive heart failure			6.239	3.691	10.545	**<0.0001**
Coronary artery disease			1.818	1.178	2.806	**0.0069**
**Model 1**	AUC	N	Odds ratio	L 95%CI	U 95%CI	p-value
Atrial fibrillation	0.7675	5672 (172 events)				
Q8NFT8 (DNER) [SD]			0.690	0.592	0.803	**<0.0001**
Age [y]			1.083	1.063	1.103	**<0.0001**
Sex (Women)			0.540	0.392	0.746	**0.00018**
**Model 2**	AUC	N	Odds ratio	L 95%CI	U 95%CI	p-value
Atrial fibrillation	0.7746	5635 (171 events)				
Q8NFT8 (DNER) [SD]			0.706	0.603	0.825	**<0.0001**
Age [y]			1.075	1.053	1.096	**<0.0001**
Sex (Women)			0.578	0.416	0.804	**0.0011**
Smoking			0.910	0.561	1.477	0.70
Arterial hypertension			1.196	0.826	1.733	0.34
Diabetes mellitus			1.152	0.797	1.663	0.45
Obesity			1.008	0.717	1.418	0.96
Dyslipidemia			1.600	1.149	2.228	**0.0054**
**Model 3**	AUC	N	Odds ratio	L 95%CI	U 95%CI	p-value

Atrial fibrillation	0.8018	5543 (162 events)				
Q8NFT8 (DNER) [SD]			0.766	0.649	0.904	**0.0017**
Age [y]			1.067	1.045	1.089	**<0.0001**
Sex (Women)			0.555	0.390	0.790	**0.0011**
Smoking			1.006	0.617	1.641	0.98
Arterial hypertension			1.100	0.750	1.615	0.63
Diabetes mellitus			1.218	0.828	1.790	0.32
Obesity			0.943	0.659	1.350	0.75
Dyslipidemia			1.468	1.023	2.107	**0.037**
Congestive heart failure			6.432	3.808	10.863	**<0.0001**
Coronary artery disease			1.599	1.032	2.479	**0.036**
**Model 1**	AUC	N	Odds ratio	L 95%CI	U 95%CI	p-value
Atrial fibrillation	0.7656	5672 (172 events)				
P02778 (CXCL10) [SD]			1.556	1.316	1.841	**<0.0001**
Age [y]			1.079	1.060	1.100	**<0.0001**
Sex (Women)			0.501	0.363	0.693	**<0.0001**
**Model 2**	AUC	N	Odds ratio	L 95%CI	U 95%CI	p-value
Atrial fibrillation	0.7747	5635 (171 events)				
P02778 (CXCL10) [SD]			1.529	1.290	1.813	**<0.0001**
Age [y]			1.072	1.051	1.094	**<0.0001**
Sex (Women)			0.536	0.386	0.747	**0.00022**
Smoking			1.035	0.637	1.682	0.89
Arterial hypertension			1.204	0.833	1.739	0.32
Diabetes mellitus			1.101	0.763	1.588	0.61
Obesity			1.101	0.788	1.538	0.57
Dyslipidemia			1.620	1.164	2.254	**0.0042**
**Model 3**	AUC	N	Odds ratio	L 95%CI	U 95%CI	p-value
Atrial fibrillation	0.8003	5543 (162 events)				
P02778 (CXCL10) [SD]			1.455	1.217	1.741	**<0.0001**
Age [y]			1.062	1.041	1.084	**<0.0001**
Sex (Women)			0.523	0.367	0.745	**0.00034**
Smoking			1.119	0.685	1.830	0.65
Arterial hypertension			1.112	0.759	1.630	0.59
Diabetes mellitus			1.171	0.797	1.721	0.42
Obesity			0.986	0.692	1.406	0.94
Dyslipidemia			1.470	1.024	2.110	**0.037**
Congestive heart failure			6.609	3.918	11.150	**<0.0001**
Coronary artery disease			1.735	1.122	2.682	**0.013**
**Model 1**	AUC	N	Odds ratio	L 95%CI	U 95%CI	p-value
Atrial fibrillation	0.7517	5672 (172 events)				
O15169 (AXIN1) [SD]			0.812	0.698	0.943	**0.0065**
Age [y]			1.092	1.072	1.112	**<0.0001**
Sex (Women)			0.536	0.389	0.740	**0.00015**
**Model 2**	AUC	N	Odds ratio	L 95%CI	U 95%CI	p-value

Atrial fibrillation	0.7662	5635 (171 events)				
O15169 (AXIN1) [SD]			0.789	0.677	0.919	**0.0023**
Age [y]			1.081	1.060	1.103	**<0.0001**
Sex (Women)			0.580	0.418	0.806	**0.0012**
Smoking			0.926	0.571	1.501	0.76
Arterial hypertension			1.231	0.852	1.778	0.27
Diabetes mellitus			1.138	0.789	1.641	0.49
Obesity			1.187	0.851	1.656	0.31
Dyslipidemia			1.704	1.226	2.369	**0.0015**
**Model 3**	AUC	N	Odds ratio	L 95%CI	U 95%CI	p-value
Atrial fibrillation	0.7944	5543 (162 events)				
O15169 (AXIN1) [SD]			0.816	0.696	0.958	**0.013**
Age [y]			1.071	1.049	1.093	**<0.0001**
Sex (Women)			0.556	0.391	0.791	**0.0011**
Smoking			1.020	0.626	1.663	0.94
Arterial hypertension			1.123	0.766	1.645	0.55
Diabetes mellitus			1.224	0.833	1.799	0.30
Obesity			1.060	0.744	1.509	0.75
Dyslipidemia			1.538	1.073	2.206	**0.019**
Congestive heart failure			6.880	4.089	11.576	**<0.0001**
Coronary artery disease			1.672	1.081	2.584	**0.021**
**Model 1**	AUC	N	Odds ratio	L 95%CI	U 95%CI	p-value
Atrial fibrillation	0.7534	5672 (172 events)				
P49771 (Flt3L) [SD]			1.226	1.044	1.440	**0.013**
Age [y]			1.088	1.067	1.108	**<0.0001**
Sex (Women)			0.517	0.375	0.714	**<0.0001**
**Model 2**	AUC	N	Odds ratio	L 95%CI	U 95%CI	p-value
Atrial fibrillation	0.7657	5635 (171 events)				
P49771 (Flt3L) [SD]			1.222	1.040	1.437	**0.015**
Age [y]			1.078	1.057	1.100	**<0.0001**
Sex (Women)			0.554	0.398	0.770	**0.00043**
Smoking			0.893	0.551	1.447	0.65
Arterial hypertension			1.212	0.840	1.750	0.30
Diabetes mellitus			1.063	0.738	1.532	0.74
Obesity			1.164	0.834	1.624	0.37
Dyslipidemia			1.658	1.193	2.305	**0.0026**
**Model 3**	AUC	N	Odds ratio	L 95%CI	U 95%CI	p-value
Atrial fibrillation	0.7963	5543 (162 events)				
P49771 (Flt3L) [SD]			1.186	1.004	1.401	**0.044**
Age [y]			1.068	1.046	1.090	**<0.0001**
Sex (Women)			0.540	0.380	0.769	**0.00062**
Smoking			0.984	0.602	1.607	0.95
Arterial hypertension			1.109	0.757	1.625	0.59
Diabetes mellitus			1.137	0.774	1.670	0.51
Obesity			1.028	0.722	1.465	0.88

Dyslipidemia	1.504	1.050	2.156	**0.026**
Congestive heart failure	6.780	4.023	11.424	**<0.0001**
Coronary artery disease	1.759	1.139	2.714	**0.011**

Table S8. Cox competing risk analysis for the proteins (by tertiles) identified in the cross-sectional analysis (event = atrial fibrillation, competing event = death)

(Level of significance: p < 0.05 is considered as statistically significant (z-test), in bold)

**Model 1**	Sample	HR	L95%CI	U95%CI	p-value
Atrial fibrillation ~	C-index: 0.7375, N:5396 (155 events, 107 competing events)				
P78556 (CCL20) [SD]		1.134	0.979	1.314	0.094
Age [y]		1.089	1.071	1.107	**<0.0001**
Sex (Women)		0.700	0.509	0.964	**0.029**
**Model 2**	Sample	HR	L95%CI	U95%CI	p-value
Atrial fibrillation ~	C-index: 0.7548, N:5361 (155 events, 105 competing events)				
P78556 (CCL20) [SD]		1.069	0.921	1.240	0.38
Age [y]		1.085	1.065	1.105	**<0.0001**
Sex (Women)		0.755	0.547	1.040	0.086
Smoking		1.323	0.855	2.046	0.21
Arterial hypertension		0.961	0.668	1.381	0.83
Diabetes mellitus		1.504	1.047	2.160	**0.027**
Obesity		1.358	0.971	1.900	0.074
Dyslipidemia		1.275	0.923	1.761	0.14
**Model 3**	Sample	HR	L95%CI	U95%CI	p-value
Atrial fibrillation ~	C-index: 0.7537, N:5273 (146 events, 100 competing events)				
P78556 (CCL20) [SD]		1.053	0.899	1.235	0.52
Age [y]		1.082	1.061	1.103	**<0.0001**
Sex (Women)		0.778	0.555	1.091	0.15
Smoking		1.398	0.901	2.170	0.13
Arterial hypertension		0.947	0.650	1.380	0.78
Diabetes mellitus		1.277	0.863	1.888	0.22
Obesity		1.336	0.940	1.898	0.11
Dyslipidemia		1.141	0.806	1.615	0.46
Congestive heart failure		1.860	0.892	3.876	0.098
Coronary artery disease		1.572	0.979	2.525	0.061
**Model 1**	Sample	HR	L95%CI	U95%CI	p-value
Atrial fibrillation ~	C-index: 0.7436, N:5396 (155 events, 107 competing events)				
Q8NFT8 (DNER) [SD]		0.834	0.716	0.972	**0.020**
Age [y]		1.085	1.067	1.104	**<0.0001**
Sex (Women)		0.707	0.514	0.974	**0.034**
**Model 2**	Sample	HR	L95%CI	U95%CI	p-value
Atrial fibrillation ~	C-index: 0.7589, N:5361 (155 events, 105 competing events)				
Q8NFT8 (DNER) [SD]		0.857	0.730	1.007	0.060
Age [y]		1.082	1.062	1.102	**<0.0001**

Sex (Women)		0.761	0.551	1.051	0.097
Smoking		1.336	0.865	2.064	0.19
Arterial hypertension		0.960	0.666	1.384	0.83
Diabetes mellitus		1.577	1.092	2.277	**0.015**
Obesity		1.283	0.914	1.801	0.15
Dyslipidemia		1.265	0.913	1.753	0.16
**Model 3**	Sample	HR	L95%CI	U95%CI	p-value
Atrial fibrillation ~	C-index: 0.7565, N:5273 (146 events, 100 competing events)				
Q8NFT8 (DNER) [SD]		0.899	0.761	1.063	0.21
Age [y]		1.080	1.059	1.101	**<0.0001**
Sex (Women)		0.780	0.556	1.096	0.15
Smoking		1.411	0.911	2.186	0.12
Arterial hypertension		0.949	0.650	1.386	0.79
Diabetes mellitus		1.326	0.894	1.966	0.16
Obesity		1.288	0.906	1.833	0.16
Dyslipidemia		1.140	0.803	1.618	0.46
Congestive heart failure		1.829	0.872	3.833	0.11
Coronary artery disease		1.504	0.935	2.420	0.092
**Model 1**	Sample	HR	L95%CI	U95%CI	p-value
Atrial fibrillation ~	C-index: 0.7387, N:5396 (155 events, 107 competing events)				
P02778 (CXCL10) [SD]		1.154	0.976	1.364	0.094
Age [y]		1.085	1.066	1.104	**<0.0001**
Sex (Women)		0.687	0.498	0.947	**0.022**
**Model 2**	Sample	HR	L95%CI	U95%CI	p-value
Atrial fibrillation ~	C-index: 0.7581, N:5361 (155 events, 105 competing events)				
P02778 (CXCL10) [SD]		1.158	0.983	1.365	0.079
Age [y]		1.081	1.061	1.102	**<0.0001**
Sex (Women)		0.739	0.535	1.023	0.068
Smoking		1.416	0.915	2.193	0.12
Arterial hypertension		0.967	0.672	1.393	0.86
Diabetes mellitus		1.554	1.080	2.237	**0.018**
Obesity		1.340	0.957	1.877	0.089
Dyslipidemia		1.271	0.920	1.756	0.15
**Model 3**	Sample	HR	L95%CI	U95%CI	p-value
Atrial fibrillation ~	C-index: 0.7560, N:5273 (146 events, 100 competing events)				
P02778 (CXCL10) [SD]		1.120	0.944	1.328	0.19
Age [y]		1.079	1.057	1.101	**<0.0001**
Sex (Women)		0.766	0.545	1.078	0.13
Smoking		1.474	0.949	2.290	0.084
Arterial hypertension		0.955	0.655	1.394	0.81
Diabetes mellitus		1.312	0.886	1.943	0.18
Obesity		1.322	0.929	1.882	0.12

Dyslipidemia		1.139	0.805	1.613	0.46
Congestive heart failure		1.861	0.895	3.871	0.096
Coronary artery disease		1.551	0.965	2.494	0.070
**Model 1**	Sample	HR	L95%CI	U95%CI	p-value
Atrial fibrillation ~	C-index: 0.7376, N:5396 (155 events, 107 competing events)				
O15169 (AXIN1) [SD]		0.919	0.797	1.059	0.24
Age [y]		1.089	1.071	1.108	**<0.0001**
Sex (Women)		0.703	0.511	0.968	**0.031**
**Model 2**	Sample	HR	L95%CI	U95%CI	p-value
Atrial fibrillation ~	C-index: 0.7571, N:5361 (155 events, 105 competing events)				
O15169 (AXIN1) [SD]		0.885	0.766	1.022	0.095
Age [y]		1.084	1.064	1.104	**<0.0001**
Sex (Women)		0.764	0.554	1.054	0.10
Smoking		1.348	0.871	2.085	0.18
Arterial hypertension		0.972	0.676	1.398	0.88
Diabetes mellitus		1.574	1.089	2.275	**0.016**
Obesity		1.380	0.989	1.925	0.058
Dyslipidemia		1.301	0.940	1.801	0.11
**Model 3**	Sample	HR	L95%CI	U95%CI	p-value
Atrial fibrillation ~	C-index: 0.7561, N:5273 (146 events, 100 competing events)				
O15169 (AXIN1) [SD]		0.916	0.789	1.064	0.25
Age [y]		1.081	1.061	1.103	**<0.0001**
Sex (Women)		0.784	0.559	1.101	0.16
Smoking		1.422	0.916	2.207	0.12
Arterial hypertension		0.955	0.655	1.392	0.81
Diabetes mellitus		1.326	0.891	1.972	0.16
Obesity		1.353	0.954	1.917	0.090
Dyslipidemia		1.161	0.818	1.647	0.40
Congestive heart failure		1.882	0.903	3.920	0.091
Coronary artery disease		1.533	0.946	2.485	0.083
**Model 1**	Sample	HR	L95%CI	U95%CI	p-value
Atrial fibrillation ~	C-index: 0.7360, N:5396 (155 events, 107 competing events)				
P49771 (Flt3L) [SD]		1.027	0.863	1.223	0.76
Age [y]		1.089	1.071	1.108	**<0.0001**
Sex (Women)		0.700	0.506	0.966	**0.030**
**Model 2**	Sample	HR	L95%CI	U95%CI	p-value
Atrial fibrillation ~	C-index: 0.7551, N:5361 (155 events, 105 competing events)				
P49771 (Flt3L) [SD]		1.009	0.849	1.199	0.92
Age [y]		1.085	1.065	1.105	**<0.0001**
Sex (Women)		0.758	0.547	1.050	0.095

Smoking		1.348	0.868	2.092	0.18
Arterial hypertension		0.966	0.672	1.390	0.85
Diabetes mellitus		1.531	1.064	2.203	**0.022**
Obesity		1.373	0.983	1.918	0.063
Dyslipidemia		1.282	0.927	1.773	0.13
**Model 3**	Sample	HR	L95%CI	U95%CI	p-value
Atrial fibrillation ~	C-index: 0.7545, N:5273 (146 events, 100 competing events)				
P49771 (Flt3L) [SD]		0.968	0.815	1.149	0.71
Age [y]		1.083	1.062	1.104	**<0.0001**
Sex (Women)		0.787	0.559	1.110	0.17
Smoking		1.430	0.918	2.227	0.11
Arterial hypertension		0.951	0.652	1.387	0.79
Diabetes mellitus		1.298	0.877	1.922	0.19
Obesity		1.346	0.949	1.910	0.095
Dyslipidemia		1.146	0.809	1.623	0.44
Congestive heart failure		1.895	0.907	3.960	0.089
Coronary artery disease		1.580	0.982	2.542	0.059
